# Record‐High Performance Hyperfluorescent OLEDs Achieved via Electronic Structure Control of Chlorine‐Diversified MR‐TADF Emitters

**DOI:** 10.1002/advs.202522814

**Published:** 2026-01-29

**Authors:** Taehwan Lee, Junki Ochi, Shigetada Uemura, Feiran Liu, Jiping Hao, Yasuhiro Kondo, Masahiro Hayakawa, Takuji Hatakeyama

**Affiliations:** ^1^ Department of Chemistry Graduate School of Science Kyoto University Kyoto Japan; ^2^ SK JNC Japan Co., Ltd., Chiba Ichihara Japan

**Keywords:** hyperfluorescence, late‐stage diversification, multi‐resonance effect, organic light‐emitting diode, thermally activated delayed fluorescence

## Abstract

Multiple‐resonance (MR)‐type thermally activated delayed fluorescence (TADF) emitters enable highly efficient and narrowband organic light‐emitting diodes (OLEDs), yet their electronic tunability has remained limited. Here, we demonstrate that chlorine‐enabled late‐stage diversification of the MR scaffold ω‐DABNA allows systematic control of ionization potential and electron affinity, leading to record‐high performance in hyperfluorescent (HF) OLEDs. Three derivatives, ω‐DABNA‐4TBP, ω‐DABNA‐4CzP, and ω‐DABNA‐4CNP, were synthesized via a single‐step Suzuki–Miyaura coupling, exhibiting narrowband green emission (FWHM = 23–26 nm) and remarkably high horizontal orientation (*Θ*_h_ = 85%–87%). Among them, ω‐DABNA‐4TBP achieved an external quantum efficiency (EQE_max_) of 36.1% and maintained 33.2% at 1000 cd m^−2^, together with outstanding operational stability (LT_95_ ≈ 385 h). This performance represents one of the highest ever reported for HF‐OLEDs. The other derivatives exhibited comparable efficiencies but much shorter lifetimes, revealing that subtle modulation of electronic structure critically governs exciton dynamics and device durability. This study establishes chlorine‐guided electronic structure control as a versatile platform for developing next‐generation MR‐TADF emitters combining narrowband emission, record‐high efficiency, and long‐term operational stability.

## Graphical abstract

A late‐stage diversification strategy for the benchmark MR‐TADF emitter ω‐DABNA enables precise electronic structure control via chlorine‐activated functionalization. The resulting derivatives achieve tunable emission from 523 to 533 nm with record‐high EQE_1000_ up to 34.0% and excellent color purity (CIEy = 0.70–0.73), establishing a versatile platform for high‐performance hyperfluorescent OLEDs.

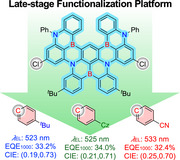

## 1 Introduction

Organic light‐emitting diodes (OLEDs) have achieved remarkable progress through the evolution of diverse emitter systems, from fluorescence and heavy‐metal phosphorescence to purely organic thermally activated delayed fluorescence (TADF) [1, 2, 3]. Among them, multiple‐resonance (MR)‐type TADF materials have emerged over the past decade as a particularly promising class of emitters for next‐generation displays [4, 5, 6, 7, 8]. The concept of MR‐TADF was first reported by our group in 2016 through the introduction of boron–nitrogen (BN)‐embedded π‐conjugated frameworks [9, 10]. These rigid heteroatom‐doped skeletons localize the highest occupied molecular orbital (HOMO) and lowest unoccupied molecular orbital (LUMO) on distinct atomic sites, inducing short‐range charge transfer (SR‐CT) [11]. This electronic structure simultaneously minimizes the singlet–triplet energy gap (Δ*E*_ST_) to enable efficient TADF [12] and suppresses vibronic coupling in the S_1_→S_0_ transition, leading to narrow full width at half maximum (FWHM) emission and excellent color purity. Furthermore, their intrinsically rigid backbones restrict structural relaxation in the excited state, reinforcing the narrowband nature of their emission. As a result, MR‐TADF emitters have been successfully extended across the entire visible spectrum, from blue to red, while maintaining high efficiency and color purity.

**FIGURE 1:**
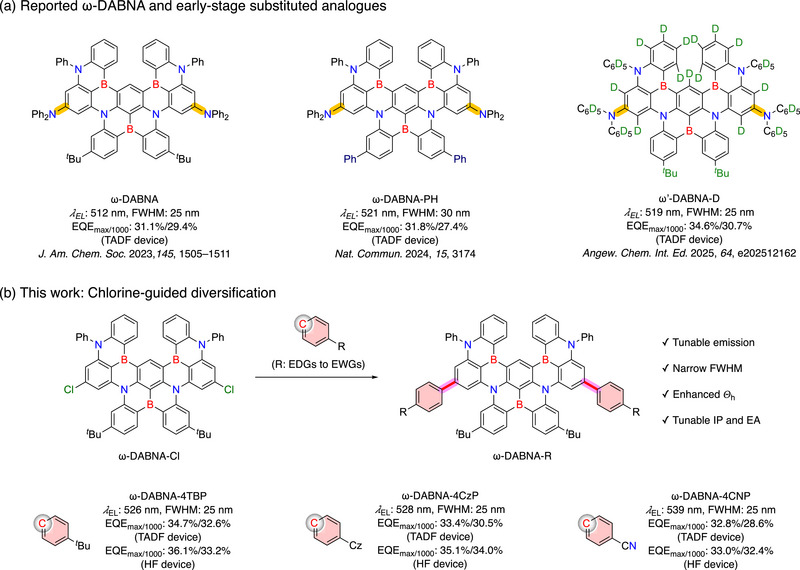
(a) ω‐DABNA and early‐stage substituted analogues. (b) Chlorine‐activated ω‐DABNA‐Cl as a platform for diverse functionalization leading to tunable derivatives.

To overcome the relatively large Δ*E*_ST_ and slow reverse intersystem crossing (RISC) rate constant that limit device operational lifetime, the hyperfluorescence (HF) strategy has been introduced [13, 14, 15, 16]. In this architecture, a TADF sensitizer with a fast RISC process first converts triplet excitons into singlets, which are subsequently transferred to an MR‐TADF terminal emitter through Förster resonance energy transfer (FRET). This sensitizer–emitter pairing combines efficient exciton harvesting with the narrowband emission of MR‐TADF molecules, thereby achieving simultaneously high efficiency, color purity, and improved stability. Recently, ω‐DABNA and its derivatives have become benchmark MR‐TADF emitters for ultrapure green emission (**Figure** 1) [17, 18, 19]. ω‐DABNA exhibits outstanding performance with a FWHM of 25 nm, a high maximum external quantum efficiency (EQE_max_) of 31.1%, and minimal efficiency roll‐off of 5.5% at 1000 cd m^−2^ [17]. Nevertheless, its emission peak at 512 nm remains slightly blue‐shifted from the ideal green region required for ultrahigh‐definition (UHD) displays. Attempts to redshift emission have focused on modifying substituents within the π‐framework (Figure 1a). However, these approaches often rely on labor‐intensive stepwise syntheses that introduce substituents in early synthetic stages, restricting structural diversification and fine‐tuning of electronic properties. Such limitations hinder the optimization of ionization potential (IP) and electron affinity (EA), which are key parameters governing energy transfer efficiency and device stability in HF‐OLED systems [20, 21, 22, 23].

To address this challenge, we developed a modular diversification strategy for ω‐DABNA by introducing a chlorine substituent to afford ω‐DABNA‐Cl (Figure 1b), which serves as a versatile platform for late‐stage functionalization. From ω‐DABNA‐Cl, we synthesized a family of derivatives, ω‐DABNA‐4TBP, ω‐DABNA‐4CzP, and ω‐DABNA‐4CNP, via a single‐step Suzuki–Miyaura cross‐coupling reaction. ω‐DABNA‐4TBP exhibited the highest overall performance, achieving an EQE_max_ of 34.7% and excellent color purity (CIE_y_ = 0.75). ω‐DABNA‐4CNP showed the most red‐shifted emission (537 nm) while maintaining a narrow FWHM of 25 nm and a high EQE_max_ of 32.8%. All derivatives displayed remarkably high horizontal molecular orientation (*Θ*_h_ = 85%–87%), significantly exceeding that of the parent ω‐DABNA (70.0%). These results demonstrate that substituent tuning through our late‐stage diversification strategy enables simultaneous control of emission color, molecular orientation, and device efficiency. In HF devices, the three derivatives exhibited narrowband green emission at 523–533 nm (FWHM = 25–26 nm) and outstanding efficiencies. ω‐DABNA‐4TBP achieved the highest EQE_max_ of 36.1% and maintained 33.2% at 1000 cd m^−2^, representing one of the best performances reported for HF‐OLEDs. The other derivatives displayed comparable efficiencies, confirming the generality of our molecular design strategy. Notably, only ω‐DABNA‐4TBP exhibited exceptional operational stability, with an LT_95_ of approximately 385 h at 1000 cd m^−2^, whereas ω‐DABNA‐4CzP and ω‐DABNA‐4CNP showed significantly shorter lifetimes (3.9 and 1.4 h). This pronounced contrast reveals that introducing a reactive chlorine site in ω‐DABNA‐Cl enables the synthesis of derivatives with finely tunable ionization potentials and electron affinities, resulting in distinct excited‐state behaviors and device stabilities. These results underscore that precise control of the electronic structure through our late‐stage diversification strategy is essential not only for maximizing efficiency but also for achieving long‐term durability in HF‐OLED systems.

## 2 Results and Discussion

**FIGURE 2:**
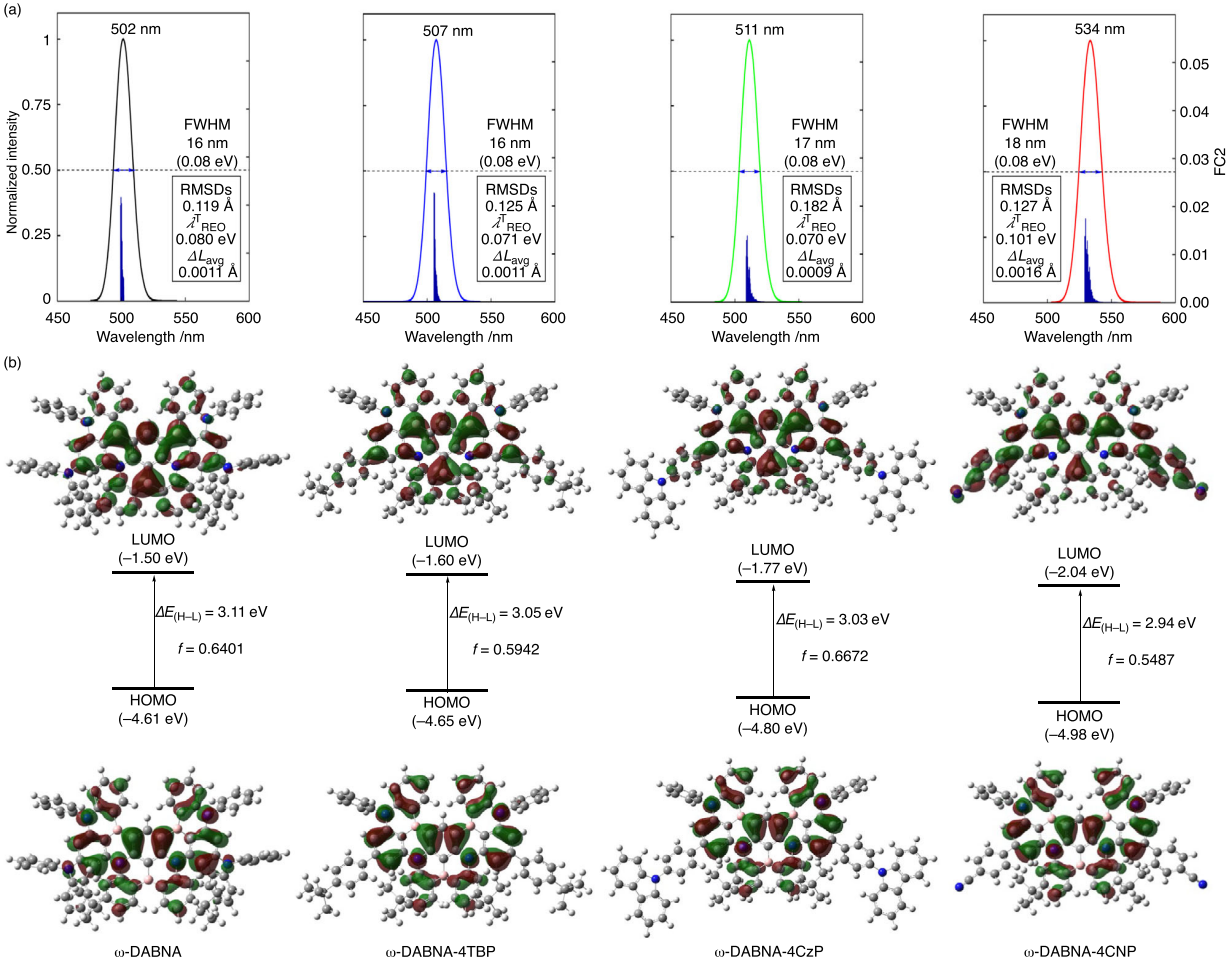
(a) Simulated emission spectra based on Franck–Condon analysis for ω‐DABNA (black), ω‐DABNA‐4TBP (blue), ω‐DABNA‐4CzP (green), and ω‐DABNA‐4CNP (red). The right y‐axis (FC2) represents the square of the Franck–Condon factors. Upon S_1_→S_0_ transition, root‐mean‐square displacements (RMSDs), the total reorganization energy ($\lambda_{\mathrm{REO}}^{T}$), and average bond length change (Δ*L*_avg_) were evaluated; the corresponding data are highlighted within the black frames in (a). (b) Kohn–Sham frontier molecular orbitals (HOMO and LUMO) of ω‐DABNA and its derivatives calculated at the B3LYP/6–31G(d) level (isovalue = 0.02). The corresponding HOMO–LUMO energy gaps (Δ*E*_(H–L)_) and oscillator strengths (*f*) are also provided.

Modifications to the ω‐DABNA framework must be carefully designed to preserve the desirable features of the original ω‐DABNA. To maintain these essential photophysical characteristics, we first performed a comprehensive series of time‐dependent (TD)‐DFT calculations on ω‐DABNA and its three derivatives. The Franck–Condon simulations, performed with the Molecular Materials Property Prediction Package (MOMAP) [24, 25], revealed that the FC2 intensity is predominantly localized in the principal emission band at 502, 507, 511, and 534 nm for ω‐DABNA, ω‐DABNA‐4TBP, ω‐DABNA‐4CzP, and ω‐DABNA‐4CNP, respectively. This localization results in exceptionally narrow simulated FWHM values (16–18 nm), as the vibronic shoulder contributions, previously identified as a major source of spectral broadening, are effectively suppressed [10, 26, 27]. The Huang–Rhys factors (HR*_k_*) and reorganization energies (*λ*_ROE_ *_k_*) indicate that the dominant vibrational modes are associated with phenyl‐ring motions (Figures S5–S9), confirming the suppression of low‐frequency modes that induce emission broadening. In addition, the ω‐DABNA derivatives exhibit high structural rigidity, as reflected by the substantial overlap between S_0_ and S_1_ geometries (Figure S4). To quantify this, we evaluated root‐mean‐square displacements (RMSDs = 0.125–0.182 Å), total reorganization energies ($\lambda_{\mathrm{REO}}^{T}$ = 0.070–0.101 eV), and average bond length changes (Δ*L*_avg_ = 0.0009–0.0016 Å), which are widely recognized descriptors of molecular rigidity in MR‐TADF compounds [26, 27, 28, 29]. The close similarity of these values to those of the parent ω‐DABNA confirms that the structural integrity of the MR framework is preserved. These results demonstrate that the derivatives retain the same rigid architecture, thereby maintaining both narrow FWHM emission and small Stokes shifts (**Figure** 2).

Next, the excited state properties of ω‐DABNA and its three derivatives were investigated. Because conventional TD‐DFT tends to overestimate S_1_ energies [11, 30, 31], we adopted a more reliable double‐hybrid (DH)‐TDDFT approach using TDA‐B2PLYP (*c_x_* = 0.40, *c_c_* = 0.25)/cc–pVDZ//M06–2X/6–31G(d) [32]. The Δ*E*_ST_ values of ω‐DABNA, ω‐DABNA‐4TBP, ω‐DABNA‐4CzP, and ω‐DABNA‐4CNP were calculated to be 75, 97, 93, and 97 meV, respectively, with corresponding spin–orbit coupling matrix elements (SOCMEs) for the S_1_–T_1_ transition of 0.016, 0.013, 0.016, and 0.017 cm^−1^. The S_1_–T_2_ energy gaps were relatively larger (289, 214, 210, and 177 meV) with higher SOCMEs (0.538, 0.354, 0.327, and 0.238 cm^−1^. The natural transition orbital (NTO) analysis revealed that all three derivatives maintain SR‐CT character in both S_1_ and T_1_ states (Figure S3). The similarity of these calculated parameters suggests that the ω‐DABNA derivatives are likely to exhibit excellent TADF characteristics consistent with their SR‐CT nature and expected triplet harvesting efficiency. On the other hand, (DH)‐TDDFT calculations of S_1_ excitation energies, 2.357 eV (ω‐DABNA, 526 nm), 2.331 eV (ω‐DABNA‐4TBP, 532 nm), 2.314 eV (ω‐DABNA‐4CzP, 536 nm), and 2.280 eV (ω‐DABNA‐4CNP, 544 nm), predict a systematic bathochromic shift in emission upon substitution. Frontier molecular orbital (FMO) analyses further revealed substantial modulation of the HOMO and LUMO levels relative to the parent molecule, with calculated energies of –4.61/–1.50 eV for ω‐DABNA, –4.65/–1.60 eV for ω‐DABNA‐4TBP, –4.80/–1.77 eV for ω‐DABNA‐4CzP, and –4.98/–2.04 eV for ω‐DABNA‐4CNP (Figure 2). These results underscore that the chlorine‐activated ω‐DABNA framework serves as a versatile platform for concurrent tuning of optical and electronic properties through substituent modification.

**SCHEME 1:**
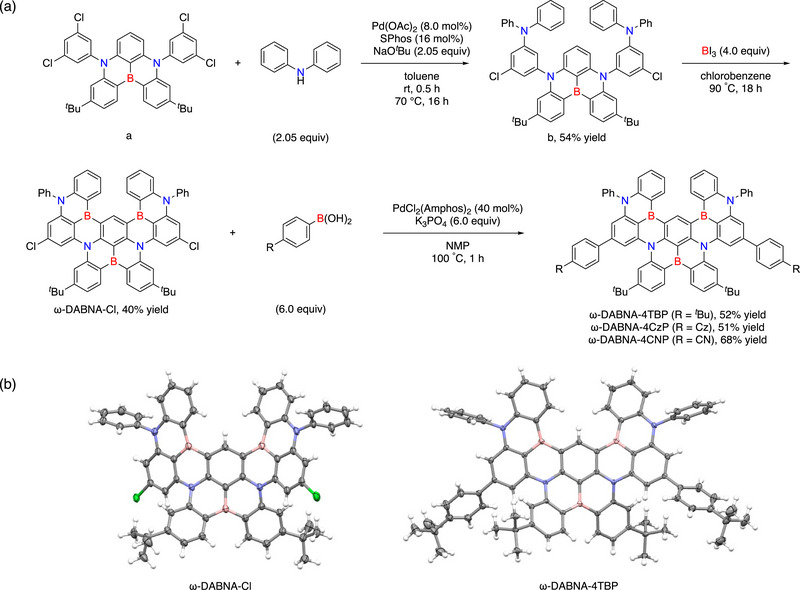
(a) Synthesis of ω‐DABNA‐4TBP, ω‐DABNA‐4CzP, and ω‐DABNA‐4CNP. (b) X‐ray crystal structure of ω‐DABNA‐Cl and ω‐DABNA‐4TBP. Molecular structures are drawn in thermal ellipsoid plots (50% probability for thermal ellipsoids; gray, carbon; white, hydrogen; pink, boron; blue, nitrogen; green, chlorine).

The synthetic routes for ω‐DABNA‐4TBP, ω‐DABNA‐4CzP, and ω‐DABNA‐4CNP are summarized in **Scheme** 1. Compound b was synthesized via additional Buchwald–Hartwig amination following the reported sequential multiple borylation procedure [17]. Compound ω‐DABNA‐Cl was synthesized in 40% yield through one‐shot borylation [33], serving as a versatile intermediate for late‐stage functionalization. Suzuki–Miyaura cross‐coupling of compounds ω‐DABNA‐Cl with 4‐R‐PhB(OH)_2_ (6 equiv, R = *^t^* Bu, Cz, and CN) in the presence of PdCl_2_(Amphos)_2_ (40 mol%) and a base at 100°C afforded ω‐DABNA‐4TBP, ω‐DABNA‐4CzP, and ω‐DABNA‐4CNP in 51%–68% yield. All products were characterized by ^1^H, ^13^C, and ^11^B NMR spectroscopy and high‐resolution mass spectrometry (HRMS), as detailed in the Supporting Information. Single crystals suitable for X‐ray diffraction were obtained for ω‐DABNA‐Cl and ω‐DABNA‐4TBP (Scheme 1) by the vapor diffusion method (Figures S13 and S14), confirming the retention of the rigid 3D ω‐DABNA geometry.

**FIGURE 3:**
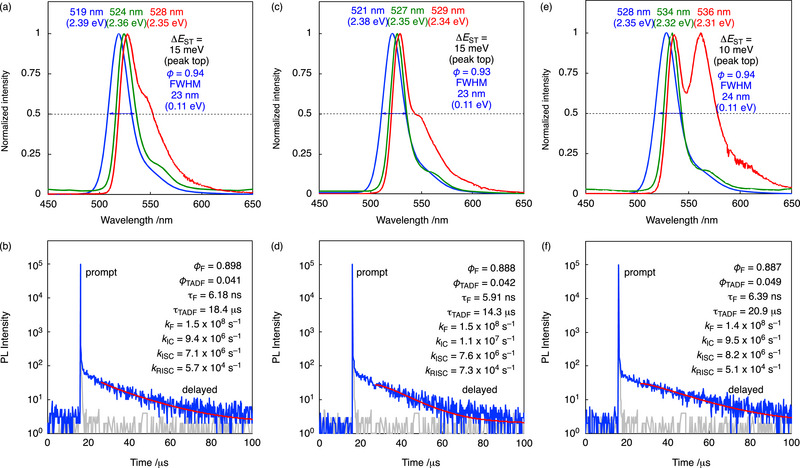
Photophysical properties of 1 wt.%‐doped PMMA films of (a,b) ω‐DABNA‐4TBP, (c,d) ω‐DABNA‐4CzP, and (e,f) ω‐DABNA‐4CNP (excited at 365 nm). (a,c,e) Steady‐state photoluminescence (PL) spectra at 300 K (blue) and 77 K (green), and phosphorescence spectra at 77 K (red) with a 25 ms delay. (b,d,f) Transient PL decay curves at 300 K, along with their relevant parameters. The gray curves indicate the instrument response function (IRF). The red curves represent single‐exponential fitting results. (background = 2).

The photophysical properties of 1 wt.%‐ ω‐DABNA‐4TBP, ω‐DABNA‐4CzP, and ω‐DABNA‐4CNP‐doped films of poly(methyl methacrylate) (PMMA) are shown in **Figure** 3 and **Table** 1. The parent compound ω‐DABNA exhibits an emission maximum at 509 nm with a narrow FWHM of 22 nm and a high photoluminescence quantum yield (PLQY) of 87% and a small Δ*E*_ST_ of 13 meV (Figure S11b) [17]. The three derivatives show systematically bathochromic‐shifted emission peaks at 519, 521, and 528 nm, respectively, while maintaining narrow emission bandwidths (FWHM = 23–24 nm). This bathochromic shift is consistent with the slight redshift observed in the absorption spectra relative to ω‐DABNA (Figure S11a,d,e,f), which originates from a net reduction of the HOMO−LUMO gap caused by more pronounced stabilization of the LUMO upon substitution (Figure 1a; Table S1). Notably, all derivatives exhibit improved PLQYs, reaching up to 94%. These results suggest that peripheral substitution effectively tunes the emission wavelength while preserving spectral purity and even improving radiative efficiency. From the fluorescence and phosphorescence emission maxima measured at 77 K (Figure 3a,c,e), Δ*E*_ST_ was estimated from the peak positions to be 10–15 meV, comparable to that of ω‐DABNA (13 meV) and markedly smaller than those previously reported for pure‐green MR‐TADF emitters [17, 18, 19, 34, 35, 36, 37, 38, 39, 40, 41, 42, 43, 44, 45]. Such small Δ*E*_ST_ values are indicative of efficient RISC and favorable TADF characteristics. Transient photoluminescence measurements revealed prompt and delayed lifetimes of 5.9–6.4 ns and 14–21 µs, respectively, which are longer than that of ω‐DABNA. The experimentally determined rate constant of RISC (*k*_RISC_) values, calculated by Adachi's method [46, 47, 48], were 5.7 × 10^4^, 7.3 × 10^4^, and 5.1 × 10^4^ s^−1^ for three compounds, lower than that of ω‐DABNA (1.2 × 10^5^ s^−1^) (Figure 3b,d,e; Figure S11c). According to the expression for *k*_RISC_∝ <$S_{n}|{\hat{H}}_{SOC}$|*T_n_* >^2^/Δ*E*_ST_ [49, 50, 51, 52], the relatively smaller SOCMEs of the derivatives account for their smaller *k*_RISC_ values, despite similar Δ*E*_ST_ values. Nevertheless, these derivatives exhibit improved horizontal molecular orientation (*Θ*_h_) from 70.0% to 84.8–87.0% (Figure S16), together with high PLQYs and narrow emission bandwidths. The improved *Θ*_h_ can be explained by the extended and rigid molecular framework of ω‐DABNA‐4TBP, ω‐DABNA‐4CzP, and ω‐DABNA‐4CNP, which leads to an increased effective molecular aspect ratio and favors horizontal alignment during vacuum deposition [53, 54]. Although their intrinsic TADF rates are slightly reduced, this combination of enhanced molecular orientation, efficient radiative emission, and spectral sharpness is expected to be highly advantageous for HF‐OLED applications.

**TABLE 1:** Photophysical properties of ω‐DABNA, ω‐DABNA‐4TBP, ω‐DABNA‐4CzP, and ω‐DABNA‐4CNP in 1‐wt.% PMMA films.

compound	*λ*_em_ ^a^ [nm]	FWHM^b^ [nm]	Δ*E*_ST_ ^c^ [meV]	*Φ* ^d^ [%]	*Φ*_F_ ^e^ [%]	*Φ*_TADF_ ^e^ [%]	*τ*_F_ ^f^ [ns]	*τ*_TADF_ ^f^ [µs]	*k*_F_ ^g^ [10^8^s^−1^]	*k*_IC_ ^g^ [10^6^s^−1^]	*k*_ISC_ ^g^ [10^6^s^−1^]	*k*_RISC_ ^g^ [10^4^s^−1^]
ω‐DABNA [17]	509	22	13	87	82	5.1	5.9	9.0	1.4	21	10	12
ω‐DABNA −4TBP	519	23	15	94	90	4.1	6.2	18	1.5	9.4	7.1	5.7
ω‐DABNA −4CzP	521	23	15	93	89	4.2	5.9	14	1.5	11	7.6	7.3
ω‐DABNA −4CNP	528	24	10	94	89	4.9	6.4	21	1.4	9.5	8.2	5.1

^a^ Maximum fluorescence wavelength;

^b^ Full width at half maximum;

^c^ Energy gap between singlet and triplet states;

^d^ Absolute photoluminescence quantum yield;

^e^ Fluorescence and TADF components determined from the total *Φ* and contribution of the integrated area of each component in the transient spectra to the total integrated area;

^f^ Lifetimes calculated from fluorescence decay;

^g^ Rate constants from fluorescence (*k*_F_), internal conversion from S_1_ to S_0_ (*k*_IC_), intersystem crossing from S_1_ to T_1_ (*k*_ISC_), and reverse intersystem crossing from T_1_ to S_1_ (*k*_RISC_) calculated from *Φ*, *Φ*_F_, *Φ*_TADF_, *τ*_F_, and *τ*_TADF_, according to the reported methods [46, 47, 48].

**FIGURE 4:**
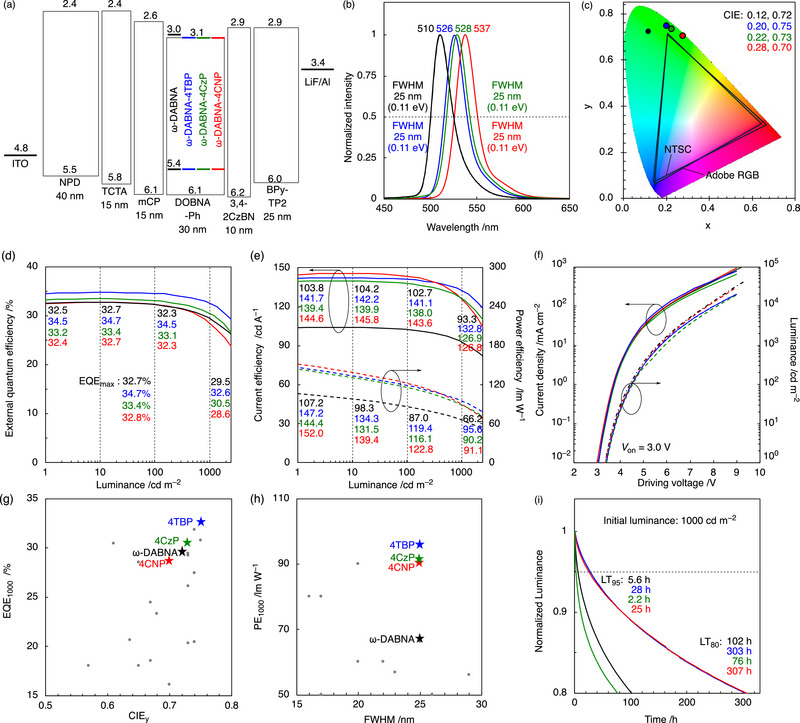
OLED characteristics of devices using ω‐DABNA, ω‐DABNA‐4TBP, ω‐DABNA‐4CzP, and ω‐DABNA‐4CNP. (a) Device structure with the estimated energy levels of each component in eV. (b) Normalized EL spectra. (c) Commission Internationale de l’Éclairage (CIE) (x,y) coordinates. (d) External quantum efficiency (EQE)–luminance (*L*) characteristics. (e) Current efficiency (CE, solid lines) and power efficiency (PE, dashed lines) as a function of luminance. (f) Current density–voltage (*J*–*V*, solid lines) and luminance–voltage (*L*–*V*, dashed lines) characteristics. (g,h) Comparison of EQE and PE values of reported green TADF‐OLEDs (CIE_y_ ≤ 0.8, FWHM ≤ 30 nm) at 1000 cd m^−2^. Highlighted in the plots are our compounds. (i) Operational stability represented by luminance decay (*L*/*L*_0_) over time at an initial luminance of 1000 cd m^−2^.

Given these favorable photophysical properties, OLED devices were fabricated to evaluate their electroluminescent performance. Device A–D were constructed with the following architecture: indium tin oxide (ITO, 50 nm), *N*,*N*′‐di(1‐naphthyl)‐*N*,*N*′‐diphenyl‐(1,1′‐biphenyl)‐4,4′‐diamine (NPD, 40 nm), tris(4‐carbazolyl‐9‐ylphenyl)amine (TCTA, 15 nm), 1,3‐bis(*N*‐carbazolyl)‐ benzene (mCP, 15 nm), 0.5 wt.% emitters: 99.5 wt.% DOBNA‐Ph (30 nm) [17, 55], 3,4‐di(9*H*‐carbazol‐9‐yl)benzonitrile (3,4‐2CzBN [56], 10 nm), 2,7‐bis(2,2′‐bipyridine‐5‐yl)triphenylene (BPy‐TP2 [57], 25 nm), LiF (1 nm), and Al (100 nm). The EL characteristics are summarized in **Figure** 4 and **Table** 2. Compared with the reference device A (ω‐DABNA, *λ*_EL_ = 510 nm, FWHM = 25 nm), the three derivative devices B−D (ω‐DABNA‐4TBP, ω‐DABNA‐4CzP, and ω‐DABNA‐4CNP) exhibited distinctly bathochromic‐shifted electroluminescence (EL) peaks at 526, 528, and 537 nm, respectively, while maintaining the same narrow emission bandwidth of 25 nm. In terms of device performance, device A showed an EQE_max_ of 32.7% and an EQE_1000_ of 29.5% (defined as the external quantum efficiency measured at a luminance of 1000 cd m^−2^). In contrast, the *^t^* Bu, Cz, and CN ‐substituted analogues exhibited higher EQE_max_ of 34.7%, 33.4%, and 32.8%, with corresponding EQE_1000_ of 32.6%, 30.5%, and 28.6%, respectively. These results clearly demonstrate that the new emitters deliver enhanced efficiency both at peak output and under high‐brightness operating conditions. This improvement is primarily attributed to the enhanced horizontal molecular orientation (*Θ*_h_ = 84.8%–87.0%), compared to 70% for ω‐DABNA (Figure S16), which facilitates more efficient light outcoupling. The orientation data of ω‐DABNA‐4CNP could not be achieved due to crystallization of the doped film, but similar behavior is anticipated based on structural similarity. A comparable enhancement was also observed in current efficiency (CE) and power efficiency (PE). While device A exhibited CE_max_/CE_1000_ values of 104.2/93.3 cd A^−1^ and PE_max_/PE_1000_ of 110.3/66.2 lm W^−1^, devices B–D achieved substantially higher values: 142.2/132.8 (B), 140.0/126.9 (C), and 145.8/126.8 cd A^−1^ (D) for CE, and 150.0/95.6 (B), 147.0/90.2 (C), and 153.8/91.1 lm W^−1^ (D) for PE. These enhancements mainly originate from the improved molecular orientation, while the slight redshift contributes to higher CE and PE through better visual sensitivity. Moreover, the present devices outperform previously reported green TADF‐OLEDs in both EQE and PE (Figure 4g,h; Table S4). At a luminance of 1000 cd m^−2^, the device lifetimes (LT_80_) were measured to be 102 h (A), 303 h (B), 76 h (C), and 307 h (D) (Figure 4i). In addition to energy‐level considerations, the relatively shorter operational lifetime of ω‐DABNA‐4CzP can be attributed to its lower intrinsic chemical robustness, as indicated by its reduced C–R bond dissociation energy compared to the other derivatives (Table S8). Although the absolute values remain limited, the extended lifetimes highlight the potential of optimized molecular design in improving both efficiency and device durability.

**TABLE 2:** Summary of device characteristics for TADF‐type devices (A–D) and HF‐type devices (E–H).

TADF‐type Devices									
Device	Dopant Host	*V*_on_ ^a^ [V]	CE_max_ ^b^ [cd A^−1^]	PE_max_ ^c^ [lm W^−1^]	EQE^d^ [%]	*λ*_em_ ^e^ [nm]	FWHM^f^ [nm]	CIE^g^ [x, y]	LT_95_[hour] @1000 cd ^h^ m^−2^
A	0.5 wt.% ω‐DABNA 99.5 wt.% DOBNA‐Ph	3.0	104.2	110.3	32.7/32.3/29.5	510	25	0.12, 0.72	5.6
B	0.5 wt.% ω‐DABNA‐4TBP 99.5 wt.% DOBNA‐Ph	3.0	142.2	150.0	34.7/34.5/32.6	526	25	0.20, 0.75	28
C	0.5 wt.% ω‐DABNA‐4CzP 99.5 wt.% DOBNA‐Ph	3.0	140.0	147.0	33.4/33.1/30.5	528	25	0.22, 0.73	2.2
D	0.5 wt.% ω‐DABNA‐4CNP 99.5 wt.% DOBNA‐Ph	3.0	145.8	153.8	32.8/32.3/28.6	537	25	0.28, 0.70	25

HF‐type Devices									
Device	Dopant Sensitizer Host	*V*_on_ ^a^ [V]	CE_max_ ^b^ [cd A^−1^]	PE_max_ ^c^ [lm W^−1^]	EQE^d^ [%]	*λ*_em_ ^e^ [nm]	FWHM^f^ [nm]	CIE^g^ [x, y]	LT_95_[hour] @1000 cd ^h^ m^−2^
E	1 wt.% ω‐DABNA 20 wt.% 4tCzBN‐PhCN 79 wt.% SiCzCz	2.5	108.7	134.0	34.8/34.5/32.4	510	25	0.15, 0.69	383
F	1 wt.% ω‐DABNA‐4TBP 20 wt.% 4tCzBN‐PhCN 79 wt.% SiCzCz	2.5	139.7	172.2	36.1/35.7/33.2	523	25	0.19, 0.73	384
G	1 wt.% ω‐DABNA‐4CzP 20 wt.% 4tCzBN‐PhCN 79 wt.% SiCzCz	2.5	135.0	155.3	35.1/35.1/34.0	525	26	0.21, 0.71	3.9
H	1 wt.% ω‐DABNA‐4CNP 20 wt.% 4tCzBN‐PhCN 79 wt.% SiCzCz	2.5	137.7	148.6	33.0/32.9/32.4	533	26	0.25, 0.70	1.4

^a^ Turn‐on voltage at 1 cd m^−2^;

^b^ Maximum current efficiency;

^c^ Maximum power efficiency;

^d^ Values at maximum, Max, 100 and 1000 cd m^−2^;

^e^ Electroluminescence maximum at 1000 cd m^−2^;

^f^ Full‐width at half‐maximum;

^g^ Commission Internationalede l’Éclairage (CIE) (x, y) coordinates;

^h^ Devices lifetime at an initial luminance of 1000 cd m^−2^.

**FIGURE 5:**
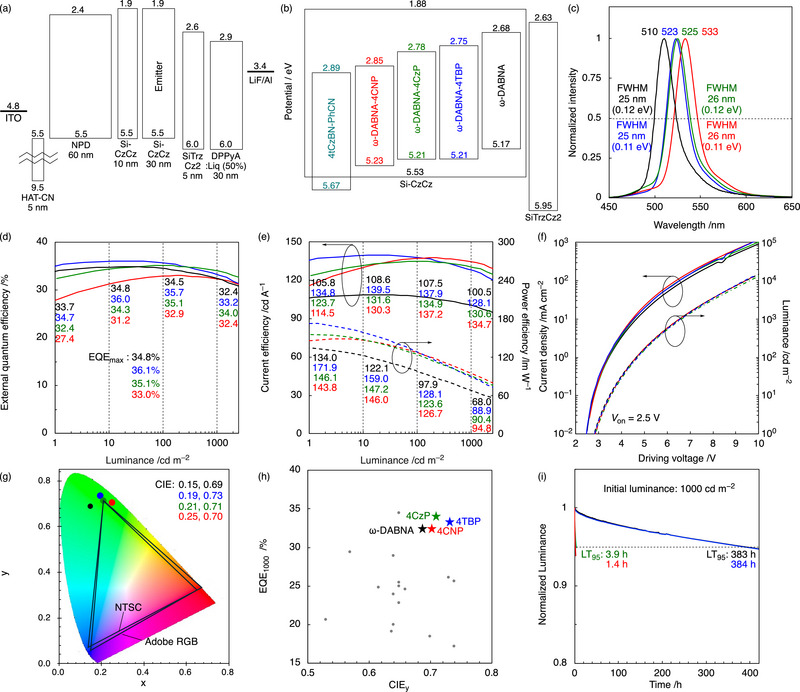
OLED characteristics of hyperfluorescent (HF) devices using ω‐DABNA, ω‐DABNA‐4TBP, ω‐DABNA‐4CzP, and ω‐DABNA‐4CNP. (a) Device structure with the estimated energy levels of each component in eV. (b) Schematic diagram of the emitting layer (EML). (c) Normalized EL spectra. (d) EQE–*L* characteristics. (e) CE (solid lines) and PE (dashed lines) as a function of luminance. (f) *J*–*V* (solid lines) and *L*–*V* (dashed line) characteristics. (g) CIE (x,y) coordinates. (h) Comparison of EQE values of reported green HF‐OLEDs (CIE_y_ ≤ 0.8) at 1000 cd m^−2^. Highlighted in the plots are our compounds. (i) Operational stability represented by luminance decay (*L*/*L*_0_) over time at an initial luminance of 1000 cd m^−2^.

Despite the improved performance of TADF‐OLED devices, the intrinsically short operational lifetime remains a critical limitation, and further improvement in efficiency roll‐off is also necessary. To address this drawback, we introduced HF system employing 4tCzBN‐PhCN as a TADF sensitizer [58], which has been reported to enhance device stability. Device E–H were constructed with following architecture: indium tin oxide (ITO, 50 nm); 1,4,5,8,9,11‐hexaazatriphenylenehexacarbonitrile (HAT‐CN, 5 nm);*N*,*N*’‐di(1‐naphthyl)‐*N*,*N*’‐diphenyl‐(1,1’‐biphenyl)‐4,4’‐diamine (NPD, 60 nm); 9‐(3‐(triphenylsilyl)phenyl)‐9*H*‐3,9'‐bicarbazole (Si‐CzCz, 10 nm) [59]; Si‐CzCz: 4tCzBN‐PhCN: emitter (30 nm, 79:20:1 wt.%); 9,9'‐(6‐(3‐(triphenylsilyl)phenyl)‐1,3,5‐triazine‐2,4‐diyl)bis(9*H*‐carbazole) (SiTrzCz2 [59], 5 nm); 9,10‐bis(6‐phenylpyridin‐3‐yl)anthracene/8‐hydroxyquinolinolato‐lithium (50%) (DPPyA/Liq, 30 nm); LiF (1 nm); Al (100 nm). The EL characteristics are shown in **Figure** 5 and Table 2. Compared with the reference device E (ω‐DABNA) (*λ*_EL_ = 510 nm, FWHM = 25 nm), the three derivative devices F−H (ω‐DABNA‐4TBP, ω‐DABNA‐4CzP, and ω‐DABNA‐4CNP) exhibited distinctly bathochromic‐shifted EL peaks at 523, 525, and 533 nm, respectively, while maintaining narrowband emission with FWHM of 25–26 nm and remarkably high EQE_max_ values of 36.1%, 35.1%, and 33.0%, respectively. Even at a luminance of 1000 cd m^−2^, the devices maintained outstanding efficiencies of 33.2%, 34.0%, and 32.4%, corresponding to suppressed efficiency roll‐offs of 8.0%, 3.1%, and 1.8%, respectively (Table 2). In addition, the corresponding CE_max_ values reached 139.7, 135.0, and 137.7 cd A^−1^, while the PE_max_ values were 172.2, 155.3, and 148.6 lm W^−1^. Compared with devices A−D, devices E−H exhibited substantially improved performance, as evidenced by their higher EQE, CE, and PE, reduced efficiency roll‐off, and lower turn‐on voltage (*V*_on_). Notably, the EQE_max_, when plotted as a function of CIE coordination, remained close to the target coordinates of standard color gamuts such as Adobe RGB and NTSC (Figure 5f). When benchmarked against previously reported HF systems, these devices exhibited outstanding performance. Particularly they achieved the highest EQE values at 1000 cd m^−2^ (Figure 5g). Moreover, analysis of CE and PE values as a function of FWHM revealed that the present devices not only rank among the highest‐performing systems but also maintain color purity (Figure S18; Table S5). Regarding operational stability, devices E and F exhibited remarkably long lifetimes (LT_95_) of 383 and 384 h, respectively. Additionally, by adopting a degradation acceleration factor that relates luminance to lifetime, namely, with 80% of the initial luminance, LT_80_ (1000 cd m^−2^) = LT_80_ (5000 cd m^−2^) × (5000 cd m^−2^/1000 cd m^−2^)*^n^* with *n* = 1.8, the extrapolated lifetime of devices E and F were estimated to exceed 3000 h at an initial luminance of 1000 cd m^−2^ (Table S6). In contrast, devices G and H showed significantly shorter lifetimes of only 3.9 and 1.4 h, respectively. Although the electrochemical measurements revealed only minor changes in the redox potentials (Figure S12 and Table S3), these differences induce a mismatch in the IP/EA alignment between the TADF sensitizer and the terminal emitter, which alters charge trapping behavior and the dominant recombination pathway (Figure 5b). Under efficient hyperfluorescent operation, electrons are preferentially trapped on the EA level of the TADF sensitizer, where charge recombination occurs, followed by exciton transfer to the terminal emitter via FRET. In devices G (**ω‐DABNA‐4CzP**) and H (**ω‐DABNA‐4CNP**), however, their EA levels of the terminal emitters are deeper than those in devices E and F and closely aligned with that of the sensitizer. This energy‐level configuration facilitates electron transfer from the sensitizer to the terminal emitter, such that electrons are no longer stably trapped on the sensitizer but instead become trapped on the terminal emitter. As a result, both electrons and holes accumulate on the terminal emitter molecules. Consequently, charge recombination predominantly occurs directly on the terminal emitter. This recombination pathway disrupts the intended hyperfluorescent operation, leads to device characteristics resembling TADF operation, and ultimately gives rise to the markedly reduced operational lifetimes observed for devices G and H. By contrast, devices E (**ω‐DABNA**) and F (**ω‐DABNA‐4TBP**) benefit from favorable IP/EA offsets between the sensitizer and the terminal emitter, which stabilize charge trapping on the sensitizer and preserve the intended hyperfluorescent recombination pathway, resulting in substantially extended operational lifetimes. These findings underscore that controlling the charge recombination pathway through appropriate energy‐level alignment is essential for simultaneously achieving high efficiency and long operational lifetimes in HF‐OLEDs.

## 3 Conclusion

In conclusion, we developed a late‐stage diversification strategy for ω‐DABNA by introducing a reactive chlorine site (ω‐DABNA‐Cl), enabling the facile synthesis of a series of electronically diverse derivatives while preserving the rigid MR‐TADF framework. This approach allows precise post‐modification through Suzuki–Miyaura cross‐coupling, providing a versatile platform for tuning both emission color and frontier orbital energies in a controlled manner. The obtained derivatives exhibit narrowband green emission (FWHM = 23–26 nm), small Δ*E*_ST_ (10–15 meV), and high photoluminescence quantum yields, together with improved horizontal molecular orientation. These photophysical features translate into excellent device performances, achieving EQE_max_ values up to 36% with minimal efficiency roll‐off and high color purity in both TADF and hyperfluorescent OLEDs. Importantly, we found that subtle differences in ionization potential and electron affinity critically determine operational stability. Devices with well‐balanced energy offsets between the sensitizer and terminal emitter achieved extended lifetimes exceeding 300 h, whereas poor energetic matching led to rapid degradation. This correlation reveals that the tunability of MR emitters extends beyond color control to directly govern energy‐transfer dynamics and device durability. Overall, this study establishes a practical molecular design concept that integrates color purity, efficiency, and stability through electronic structure control, offering new guidelines for developing next‐generation MR‐TADF emitters and high‐performance OLED technologies.

[CCDC 2494154 and 2494155 contains the supplementary crystallographic data for this paper. These data can be obtained free of charge from The Cambridge Crystallographic Data Centre via www.ccdc.cam.ac.uk/data_request/cif.]

## Conflicts of Interest

The authors declare no conflicts of interest.

## Supporting information




**Supporting File**: advs74133‐sup‐0001‐Supp‐Info.pdf.

## Data Availability

The data that support the findings of this study are available in the supplementary material of this article.
