# Supplementary material for: Record‐High Performance Hyperfluorescent OLEDs Achieved via Electronic Structure Control of Chlorine‐Diversified MR‐TADF Emitters
Source: Adv Sci (Weinh). 2026 Jan 29;13(20):e22814. doi: 10.1002/advs.202522814 (PMC13067835; doi:10.1002/advs.202522814)
Supplement: Supplementary file 1 — Supporting File: advs74133‐sup‐0001‐Supp‐Info.pdf. [file ADVS-13-e22814-s001.pdf]

Record-High Performance Hyperfluorescent OLEDs Achieved via Electronic Structure Control of Chlorine-Diversified MR-TADF Emitters

*Taehwan Lee, Junki Ochi, Shigetada Uemura, Feiran Liu, Jiping Hao, Yasuhiro Kondo, Masahiro Hayakawa, and Takuji Hatakeyama**

T. Lee, J. Ochi, S. Uemura, F. Liu, J. Hao, M. Hayakawa, and T. Hatakeyama
Department of Chemistry, Graduate School of Science, Kyoto University, Sakyo-ku, Kyoto, 606-8502, Japan
E-mail: hatake@kuchem.kyoto-u.ac.jp
Y. Kondo
SK JNC Japan Co., Ltd. 5-1 Goikaigan, Ichihara, Chiba 290-8551, Japan

Experimental Section

General Procedure. All the reactions dealing with air- or moisture-sensitive compounds were carried out in a dry reaction vessel (small scale, a Schlenk flask, and a two-necked round bottomed flask; large scale, a three-necked round bottomed flask) under a positive pressure of nitrogen. Air- and moisture-sensitive liquids and solutions were transferred *via* a syringe or a Teflon cannula. Analytical thin-layer chromatography (TLC) was performed on glass plates coated with 0.25 mm 230–400 mesh silica gel containing a fluorescent indicator (Merck, #1.05715.0009). TLC plates were visualized by exposure to ultraviolet light (254 nm or 365 nm). Organic solutions were concentrated by rotary evaporation at *ca.* 10–50 mmHg. Flash column chromatography was performed on Merck silica gel 60 (spherical, neutral, 140–325 mesh) and Kanto Chemical silica gel 60N (spherical, neutral, 40–50 μm) as described by Still et al.[1] Proton nuclear magnetic resonance (^{1}H NMR), carbon nuclear magnetic resonance (^{13}C NMR) and boron nuclear magnetic resonance (^{11}B NMR) spectra were recorded on JEOL ECX500 (495 MHz) NMR spectrometers. Proton chemical shift values are reported in parts per million (ppm, δ scale) downfield from tetramethylsilane and are referenced to the tetramethylsilane (δ 0) or $CDCl_3$ (δ 7.26). ^{13}C NMR spectra were recorded at 125 MHz: carbon chemical shift values are reported in parts per million (ppm, δ scale) downfield from tetramethylsilane, and are referenced to the carbon resonance of tetramethylsilane (δ 0) and $CDCl_3$ (δ 77.0). ^{11}B NMR spectra were recorded at 159 MHz: boron chemical shift values are reported in parts per million (ppm, δ scale) and are referenced to the external standard boron signal of $BF_3{\cdot}Et_2O$ (δ 0). Data are presented as: chemical shift, multiplicity (s = singlet, d = doublet, t = triplet, m = multiplet and/or multiplet resonances), coupling constant in hertz (Hz), signal area integration in natural numbers, and assignment (*italic*). IR spectra were recorded on an ATR-FTIR spectrometer (IRAffinity-1S, Shimadzu and Cary 630 FTIR, Agilent Technologies). Characteristic IR absorptions are reported in cm^{-1}. Melting points were recorded on a BUCHI Melting Point M-565 (according to the limitations of the apparatus, the compounds which did not melt up to 300 °C are presented as ">300 °C"). High-resolution mass spectra (HRMS) were obtained by the matrix-assisted laser desorption/ionization (MALDI) method with a JEOL SpiralTOF instrument. UV-visible absorption spectra were measured by a Shimadzu UV-2600 instrument. Fluorescence spectra were measured by a HORIBA Scientific FluoroMax-4 instrument. Purity of isolated compounds was determined by ^{1}H NMR analyses or HPLC analysis on a JASCO UV-2070 Plus instrument equipped with a reversed-phase C18 column (Mightysil RP-18 GP, Kanto Chemical Co., Inc., 4.6 mm I.D. × 100 mm).

(1) W. C. Still, M. Kahn, A. Mitra, *J. Org. Chem.* **1978**, *43*, 2923

Materials. Materials were purchased from Wako Pure Chemical Industries, Ltd. (Wako), Tokyo Chemical Industry Co., Ltd., Aldrich Inc., and other commercial suppliers, and were used after appropriate purification, unless otherwise noted.

Solvent. Anhydrous solvents were purchased from above-described suppliers and/or dried over Molecular Sieves 4A and degassed before use. Water content of the solvent was determined with a Karl Fischer moisture titrators (AQ-2200, Hiranuma Sangyo Co., Ltd. and MKC-710B, Kyoto Electronics Manufacturing Co., Ltd.) to be less than 20 ppm.

Computational Method. All calculations were performed with Gaussian 16 (Revision C.01 or C.02)[2], PySCF[3–6] packages unless otherwise noted. The DFT method was employed using the B3LYP[7] or M06–2X[8] hybrid functional. Structures were optimized with the 6–31G(d)[9] basis set. The time-dependent density functional theory (TD-DFT) was carried out[10] with B3LYP or M06–2X/6–31G(d) level.

---

(2) Gaussian 16, Revision C.01 (C.02), M. J. Frisch, G. W. Trucks, H. B. Schlegel, G. E. Scuseria, M. A. Robb, J. R. Cheeseman, G. Scalmani, V. Barone, G. A. Petersson, H. Nakatsuji, X. Li, M. Caricato, A. V. Marenich, J. Bloino, B. G. Janesko, R. Gomperts, B. Mennucci, H. P. Hratchian, J. V. Ortiz, A. F. Izmaylov, J. L. Sonnenberg, D. Williams-Young, F. Ding, F. Lipparini, F. Egidi, J. Goings, B. Peng, A. Petrone, T. Henderson, D. Ranasinghe, V. G. Zakrzewski, J. Gao, N. Rega, G. Zheng, W. Liang, M. Hada, M. Ehara, K. Toyota, R. Fukuda, J. Hasegawa, M. Ishida, T. Nakajima, Y. Honda, O. Kitao, H. Nakai, T. Vreven, K. Throssell, J. A. Montgomery, Jr., J. E. Peralta, F. Ogliaro, M. J. Bearpark, J. J. Heyd, E. N. Brothers, K. N. Kudin, V. N. Staroverov, T. A. Keith, R. Kobayashi, J. Normand, K. Raghavachari, A. P. Rendell, J. C. Burant, S. S. Iyengar, J. Tomasi, M. Cossi, J. M. Millam, M. Klene, C. Adamo, R. Cammi, J. W. Ochterski, R. L. Martin, K. Morokuma, O. Farkas, J. B. Foresman, and D. J. Fox, Gaussian, Inc., Wallingford CT, **2016** (**2019**).

(3) Q. Sun, T. C. Berkelbach, N. S. Blunt, G. H. Booth, S. Guo, Z. Li, J. Liu, J. D. McClain, E. R. Sayfutyarova, S. Sharma, S. Wouters, G. K.-L. Chan, *WIREs Comput. Mol. Sci.* **2018**, *8*, e1340.

(4) Q. Sun, X. Zhang, S. Banerjee, P. Bao, M. Barbry, N. S. Blunt, N. A. Bogdanov, G. H. Booth, J. Chen, Z.-H. Cui, J. J. Eriksen, Y. Gao, S. Guo, J. Hermann, M. R. Hermes, K. Koh, P. Koval, S. Lehtola, Z. Li, J. Liu, N. Mardirossian, J. D. McClain, M. Motta, B. Mussard, H. Q. Pham, A. Pulkin, W. Purwanto, P. J. Robinson, E. Ronca, E. R. Sayfutyarova, M. Scheurer, H. F. Schurkus, J. E. T. Smith, C. Sun, S.-N. Sun, S. Upadhyay, L. K. Wagner, X. Wang, A. White, J. D. Whitfield, M. J. Williamson, S. Wouters, J. Yang, J. M. Yu, T. Zhu, T. C. Berkelbach, S. Sharma, A. Y. Sokolov, G. K.-L. *Chan*, *J. Chem. Phys.* **2020**, *153*, 024109.

(5) (a) https://github.com/pyscf/pyscf (b) https://jan.hermann.name/pyberny/

(6) D. Kim, Int. *J. Quantum Chem.* **2016**, *116*, 651.

(7) (a) C. Lee, W. Yang, R. G. Parr, *Phys. Rev. B* **1988**, *37*, 785; (b) A. D. Becke, *J. Chem. Phys.* **1993**, *98*, 5648.

(8) Y. Zhao, D. Truhlar, *Theor. Chem. Acc.* **2008**, *120*, 215.

(9) W. J. Hehre, L. Radom, P. v. R. Schleyer, J. A. Pople, *Ab Initio Molecular Orbital Theory*, John Wiley & Sons: New York, 1986 and references cited therein.

(10) (a) M. E. Casida, C. Jamorski, K. C. Casida, D. R. Salahub, *J. Chem. Phys.* **1998**, *108*, 4439; (b) R. E. Stratmann, G. E. Scuseria, M. J. Frisch, *J. Chem. Phys.* **1998**, *109*, 8218.

(DH-TDDFT)

Grimme's B2PLYP-type double-hybrid exchange-correlation[11,12] functional has the following energy form: $E_{xc}(c_x, c_c) = (1 - c_x)E_x^{\mathrm{B88}} + c_x E_x^{\mathrm{HF}} + (1 - c_c)E_c^{\mathrm{LYP}} + c_c E_c^{\mathrm{PT2}}$, where E_x^{B88} and E_c^{LYP} are the Becke88 exchange[13] and Lee–Yang–Parr correlation energies[14], respectively; E_x^{HF} is the Hartree–Fock (HF) exchange energy; E_c^{PT2} is the second-order perturbation correlation energy; and coefficients c_x and c_x are exchange and correlation mixing parameters. We here denote B2LYP/B2PLYP variants by B2LYP/B2PLYP (c_x = 0.40, c_c = 0.25).

Transition energies and ΔE_{ST} values were estimated for optimized S_0 geometries within double-hybrid time-dependent density functional theory (DH-TDDFT), at the TDA-B2PLYP (c_x = 0.40, c_c = 0.25)/cc–pVDZ//M062X/6–31G(d) level of theory. Due to the limit of our computation resource, we omitted virtual orbitals with energies higher than 0.6 Hartree and computed two-electron integrals in the resolution-of-the-identity approximation[15–17] for CIS(D)[18] calculations. All the DH-TDDFT computations were conducted using PySCF[4–7] with in-house CIS(D) submodules[19]. Spin-orbit coupling matrix elements (SOCME) between singlet and triplet states were evaluated in the framework of the effective one-electron formalism of Gao and coworkers[20], in which we adopted CI (configuration interaction) amplitudes at the TDA-B2LYP (c_x = 0.40, c_c = 0.25)/cc–pVDZ level in combination with effective nuclear charges developed by Koseki and coworkers[21]. We employed the Libcint library[22], which is part of PySCF, for the calculation of one-electron integrals.

Root-mean-square displacements (RMSDs) of the optimized structures at the S_0 and S_1 states were analyzed using VMD software[23]. Additionally, electron-vibration coupling and Franck-Condon spectral analysis were performed through a sum-over-states approach[24] as implemented in the Molecular Materials Property Prediction Package (MOMAP 2022B)[25]. The reorganization energy ($\lambda_{\mathrm{ROE}k}$) of vibrational mode, k, elucidated based on harmonic oscillator approximation, has a relation with its dimensionless Huang-Rhys factor (HR_k) as follows:

$$\lambda_{\mathrm{ROE}k} = \mathrm{HR}_k \hbar v_k$$

---

(11) S. Grimme, *J. Chem. Phys.* **2006**, *124*, 034108.

(12) S. Grimme, F. Neese, *J. Chem. Phys.* **2007**, *127*, 154116.

(13) A. D. Becke, *Phys. Rev. A* **1988**, *38*, 3098.

(14) C. Lee, W. Yang, E. G. Parr, *Phys. Rev. B* **1988**, *37*, 785.

(15) M. Feyereisen, G. Fitzgerald, A. Komornicki, *Chem. Phys. Lett.* **1993**, *208*, 359.

(16) O. Vahtras, J. Almlöf, M. W. Feyereisen, *Chem. Phys. Lett.* **1993**, *213*, 514.

(17) F. Weigend, M. Häser, Theor. *Chem. Acc.* **1997**, *97*, 331.

(18) M. Head-Gordon, R. J. Rico, M. Oumi, T. J. Lee, *Chem. Phys. Lett.* **1994**, *219*, 21.

(19) M. Kondo, *Chem. Phys. Lett.* **2022**, *804*, 139895.

(20) X. Gao, S. Bai, D. Fazzi, T. Niehaus, M. Barbatti, W. Thiel, *J. Chem. Theory Comput.* **2017**, *13* 515.

(21) S. Koseki, M. W. Schmidt, M. S. Gordon, *J. Phys. Chem.* **1992**, *96*, 10768.

(22) Q. Sun, *J. Comput. Chem.* **2015**, *36*, 1664.

(23) W. Humphrey, A. Dalke and K. Schulten, *J. Molec. Graphics*, 1996, **14**, 33–38.

(24) Y. Niu, W. Li, Q. Peng, H. Geng, Y. Yi, L. Wang, G. Nan, D. Wang and Z. Shuai, *Mol. Phys.* 2018, **116**, 1078–1090.

(25) a) Z. Shuai and Q. Peng, *Phys. Rep.* 2014, **537**, 123–156; b) Z. Shuai, *Chin. J. Chem.* 2020, **38**, 1223–1232.

$$\mathrm{HR}_k = \frac{v_k D_k^2}{2\hbar}$$

where v_k is the vibration frequency, and D_k denotes the normal coordinate displacement of mode k. The Huang-Rhys factor (HR_k) indicates the strength of electron-phonon coupling.

The following equation was used to calculate the average bond length change (ΔL_{avg}):

$$\Delta L_{\mathrm{avg}} = \frac{1}{n}\sum_{n=1}^{n} |E_n^{\mathrm{GS}} - E_n^{\mathrm{ES}}|$$

where E_n^{GS} is the bond length of the n-th bond in the ground states, E_n^{ES} is the bond length of the n-th bond in the excited states, and n is the total number of bonds in the molecule.

The following equation was used to calculate the total reorganization energy ($\lambda_{\mathrm{REO}}^{\mathrm{T}}$):

$$\lambda_{\mathrm{REO}}^{\mathrm{T}} = \lambda_1 + \lambda_2$$

$$= (E(\mathrm{S}^*_1) - E(\mathrm{S}_0)) + (E(\mathrm{S}^*_0) - E(\mathrm{S}_1))$$

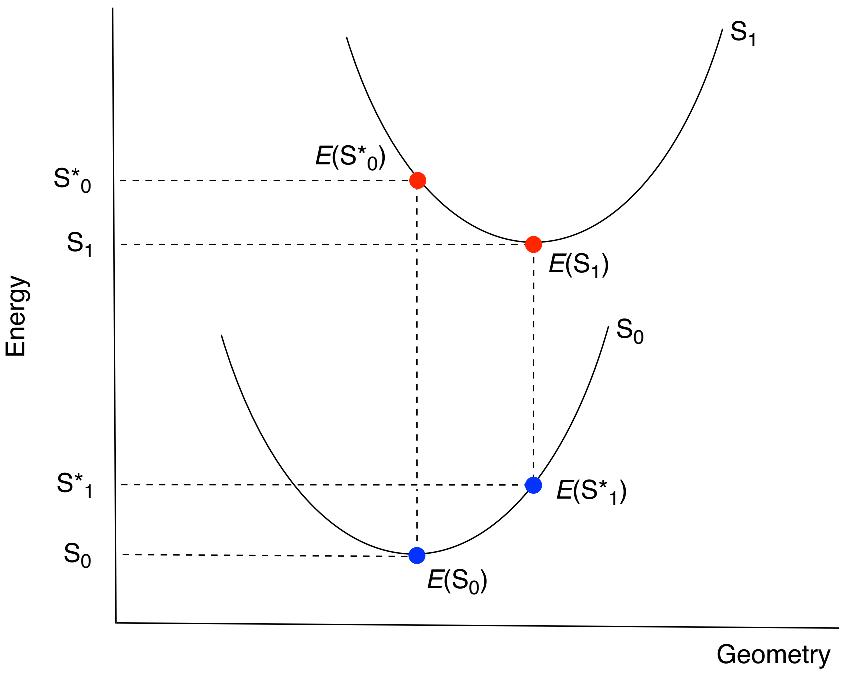

Figure S1. Potential energy surface of S_0 and S_1.

Where $E(\mathrm{S}^*_1)$, $E(\mathrm{S}_0)$, $E(\mathrm{S}^*_0)$, and $E(\mathrm{S}_1)$ are energy depending on the geometry and states.

Table S1. Summary of TD-DFT Calculation for final compounds (S_0 geometry) at the B3LYP/6–31G(d) Level of Theory.

compound	HOMO−1 [eV]	HOMO [eV]	LUMO [eV]	LUMO+1 [eV]	$\lambda_{(S0-S1)}$ [nm]	E_S[a] [eV]	f[b] [ns]	$\lambda_{(S0-T1)}$ [nm]	E_T[c] [eV]	E_{ST}[d] [eV]
**ω-DABNA**[e]	−4.93	−4.61	−1.50	−1.07	473	2.62	0.6401	530	2.34	0.28
**ω-DABNA -4TBP**	−5.03	−4.65	−1.60	−1.18	478	2.59	0.5942	543	2.28	0.31
**ω-DABNA -4CzP**	−5.18	−4.80	−1.77	−1.37	482	2.57	0.6672	546	2.27	0.30
**ω-DABNA -4CNP**	−5.36	−4.98	−2.04	−1.76	495	2.51	0.5487	561	2.21	0.30

[a] Singlet–singlet excitation energy. [b] Oscillator strength. [c] Singlet–triplet excitation energy. [d] Energy gap between S_1 and T_1 states. [e] ref. [26].

compound	S_1/T_1/T_2 energy	Coefficient of orbital			
**ω-DABNA**[a]	S_1: 2.26 eV (473 nm)	HOMO → LUMO (0.69598)			
	T_1: 2.34 eV (530 nm)	HOMO → LUMO (0.68649)			
	T_2: 2.62 eV (472 nm)	HOMO–3 → LUMO (0.32428)	HOMO–2 → LUMO+1 (0.11840)	HOMO–1 → LUMO (0.28966)	HOMO → LUMO+1 (0.50279)
**ω-DABNA -4TBP**	S_1: 2.59 eV (478 nm)	HOMO → LUMO (0.69600)			
	T_1: 2.28 eV (543 nm)	HOMO → LUMO (0.68511)	HOMO–1 → LUMO+1 (–0.12701)		
	T_2: 2.54 eV (489 nm)	HOMO–1 → LUMO (–0.45802)	HOMO–1 → LUMO+2 (0.11178)	HOMO → LUMO+1 (0.50176)	
**ω-DABNA -4CzP**	S_1: 2.57 eV (482 nm)	HOMO → LUMO (0.69568)			
	T_1: 2.27 eV (546 nm)	HOMO → LUMO (0.68324)	HOMO–1 → LUMO+1 (–0.13457)		
	T_2: 2.51 eV (493 nm)	HOMO–1 → LUMO (0.32428)	HOMO–1 → LUMO (–0.45590)	HOMO–1 → LUMO+2 (0.10060)	HOMO → LUMO+1 (0.50156)
**ω-DABNA -4CNP**	S_1: 2.51 eV (495 nm)	HOMO → LUMO (0.69442)			
	T_1: 2.21 eV (561 nm)	HOMO → LUMO (0.67314)	HOMO–1 → LUMO+1 (–0.15521)		
	T_2: 2.42 eV (513 nm)	HOMO–1 → LUMO (–0.43739)	HOMO–1 → LUMO+3 (–0.14182)	HOMO → LUMO+1 (0.51030)	

[a] ref. [26].

(26) S. Uemura, S. Oda, M. Hayakawa, R. Kawasumi, N. Ikeda, Y.-T. Lee, C.-Y, Chan, Y. Tsuchiya, C. Adachi, T. Hatakeyama. *J. Am. Chem. Soc.* **2023**, *145*, 1505–1511.

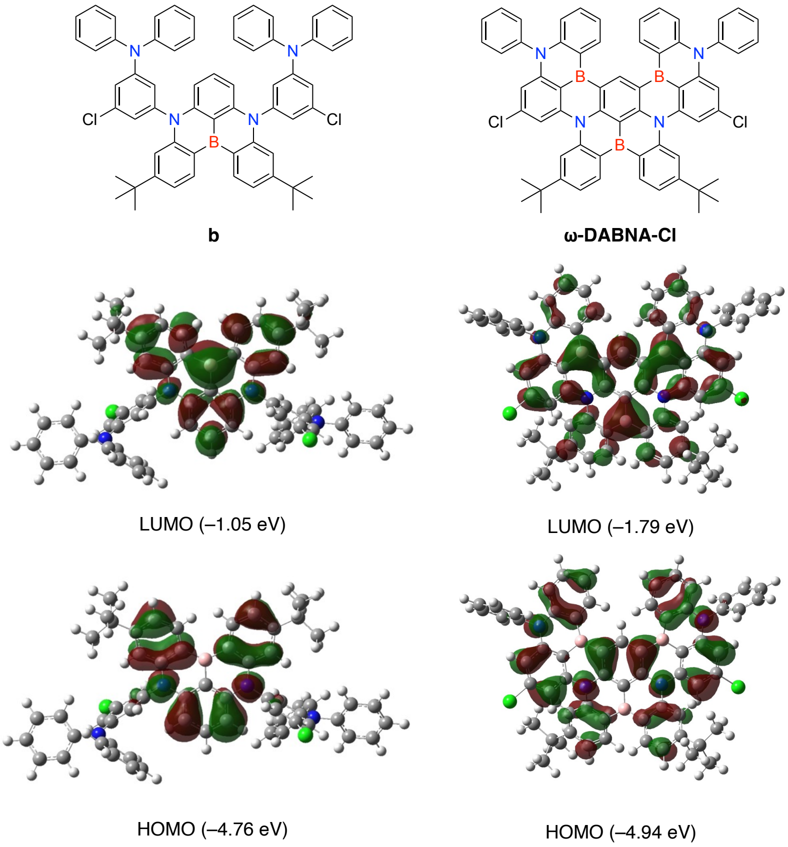

Figure S2. Kohn–Sham frontier orbitals of **b** and **ω-DABNA-Cl** calculated at the B3LYP/6–31G(d) level of theory (isovalue = 0.02).

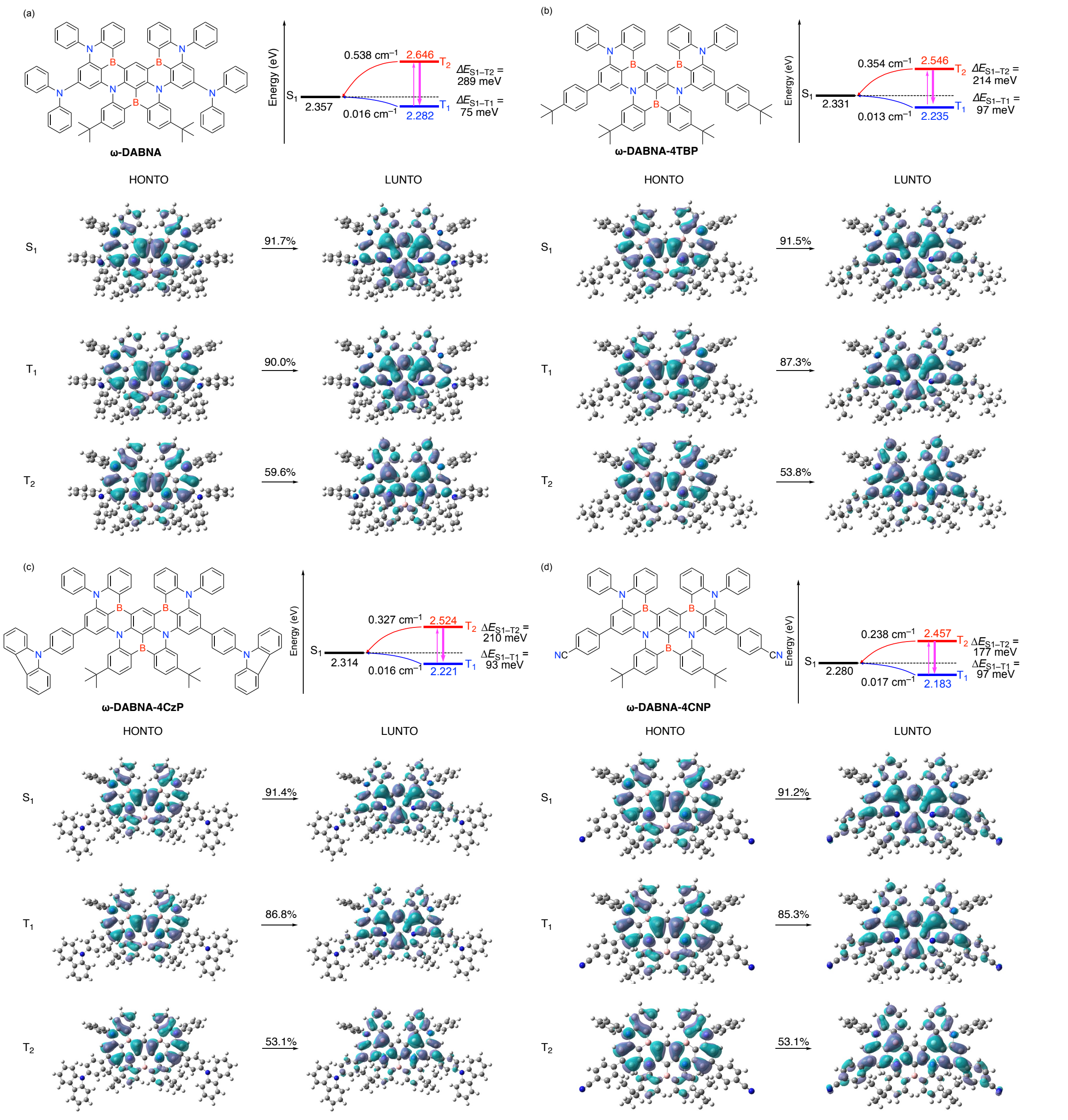

Figure S3. Energy-level diagrams and related natural transition orbitals (NTOs) for the singlet and triplet excited states of **ω-DABNA** (a), **ω-DABNA-4TBP** (b), **ω-DABNA-4CzP** (c), and **ω-DABNA-4CNP** (d) at the S_0 geometry. Transition energies for S_1, T_1, and T_2 were calculated at the TDA-B2PLYP (c_x = 0.40, c_c = 0.25)/cc–pVDZ//M06–2X/6–31G(d) levels of theory. NTOs (isovalue = 0.02) and SOC matrix elements were calculated at the TDA-B2LYP (c_x = 0.40, c_c = 0.25)/cc–pVDZ//M06–2X/6–31G(d) level of theory.

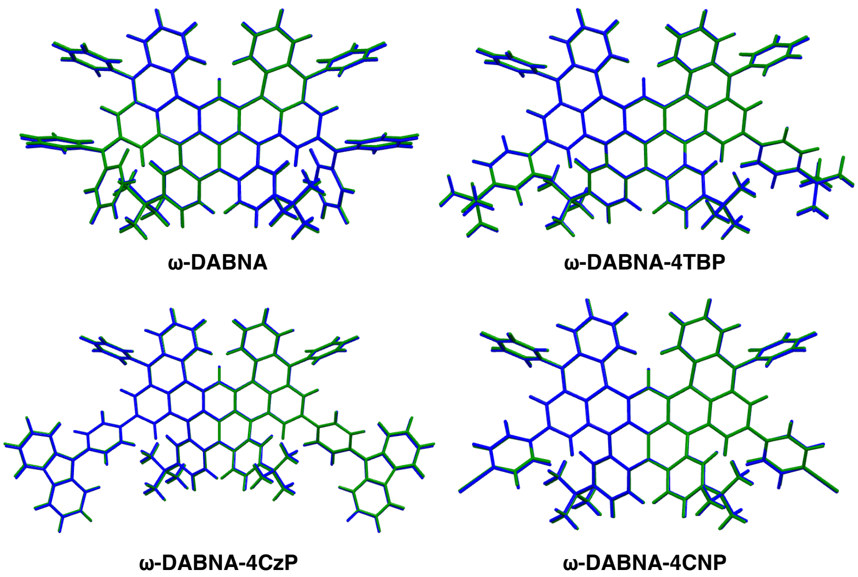

Figure S4. The geometric overlap between the optimized ground state (S_0, green) and the lowest singlet excited state (S_1, blue) structures of **ω-DABNA**, **ω-DABNA-4TBP**, **ω-DABNA-4CzP**, and **ω-DABNA-4CNP** was investigated at B3LYP/6–31G(d) level of theory.

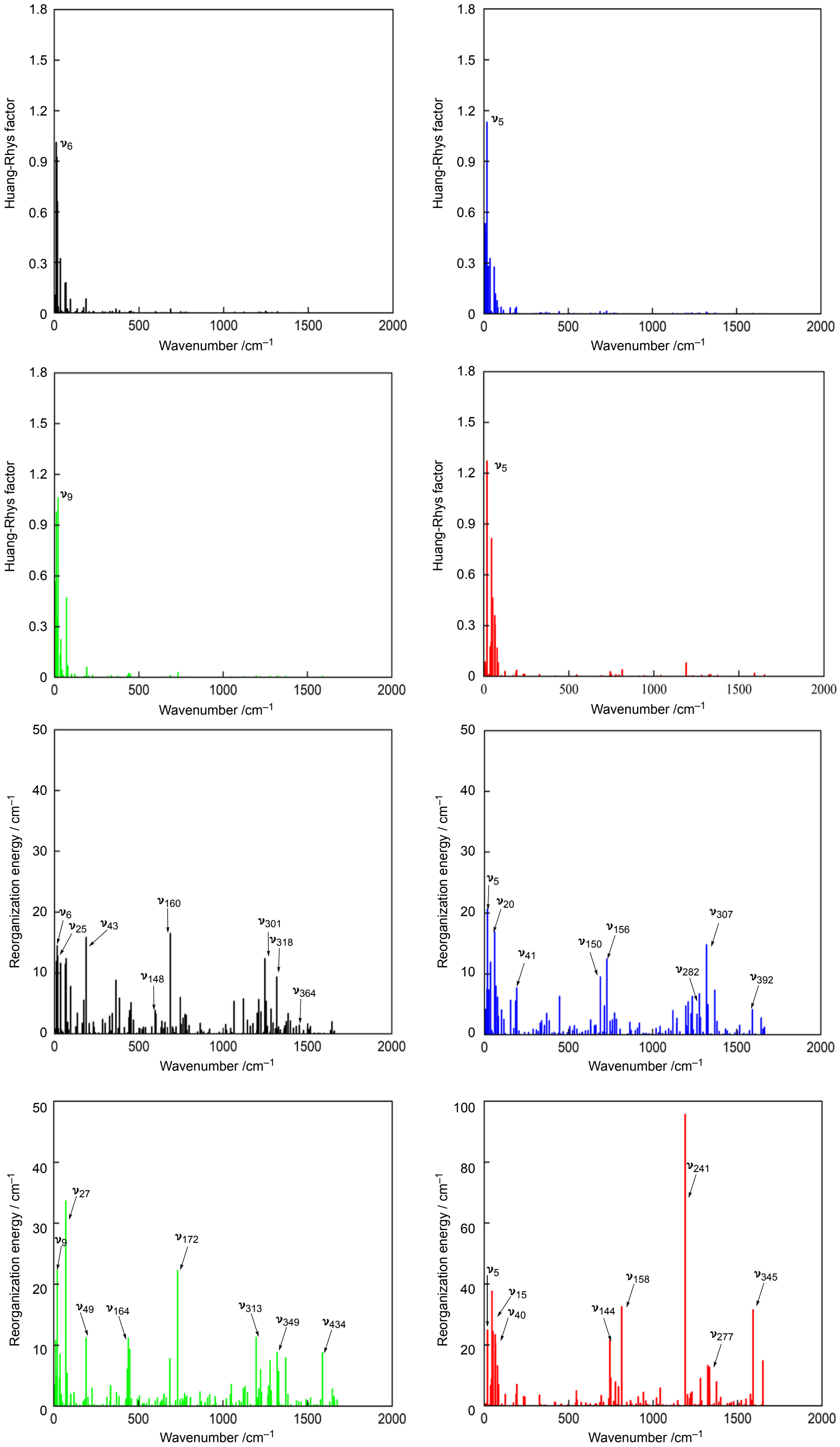

Figure S5. Huang-Rhys factor (HR$_k$), and reorganization energy ($\lambda_{\mathrm{REO}k}$) for **ω-DABNA** (black), **ω-DABNA-4TBP** (blue), **ω-DABNA-4CzP** (green), and **ω-DABNA-4CNP** (red). The Huang-Rhys factor (HR$_f$) indicates the strength of electron-phonon coupling.

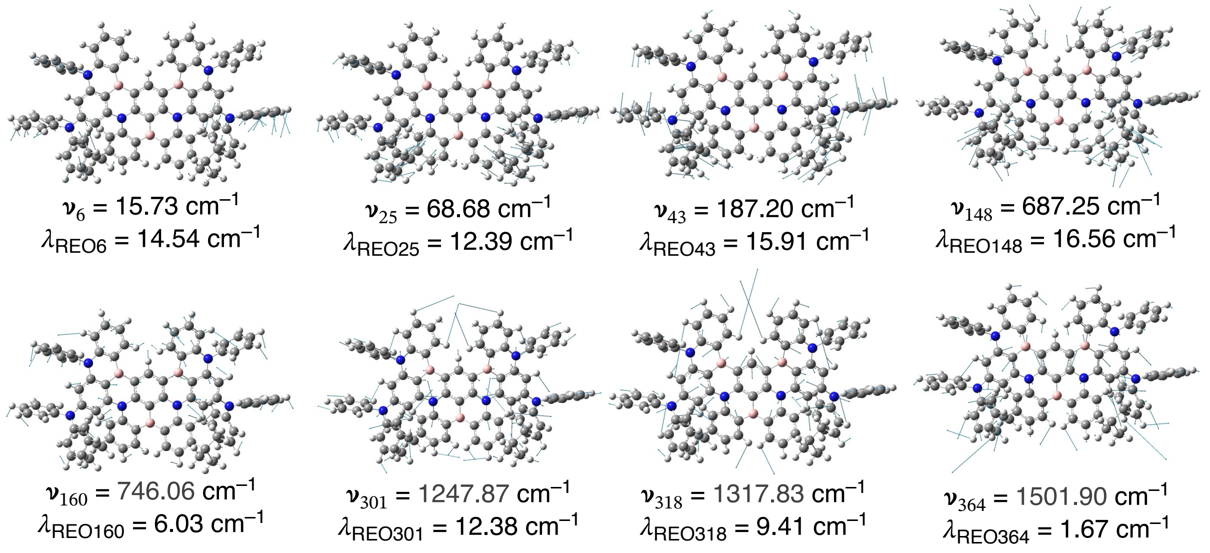

Figure S6. Vibrational modes according to the frequency and the numerical value of the reorganization energy (λ_{REOk}) of **ω-DABNA**.

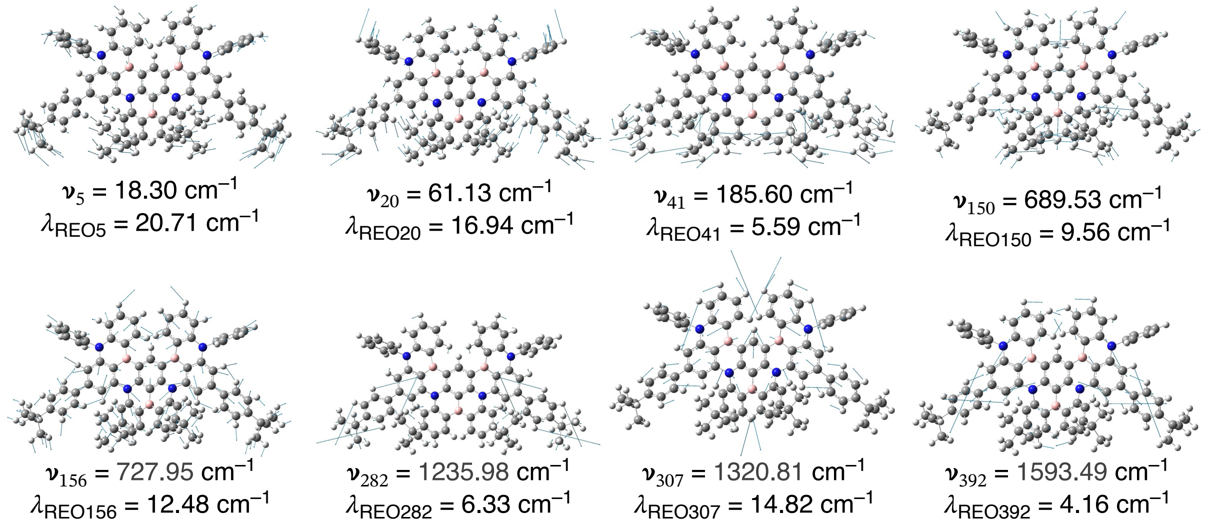

Figure S7. Vibrational modes according to the frequency and the numerical value of the reorganization energy (λ_{REOk}) of **ω-DABNA-4TBP**.

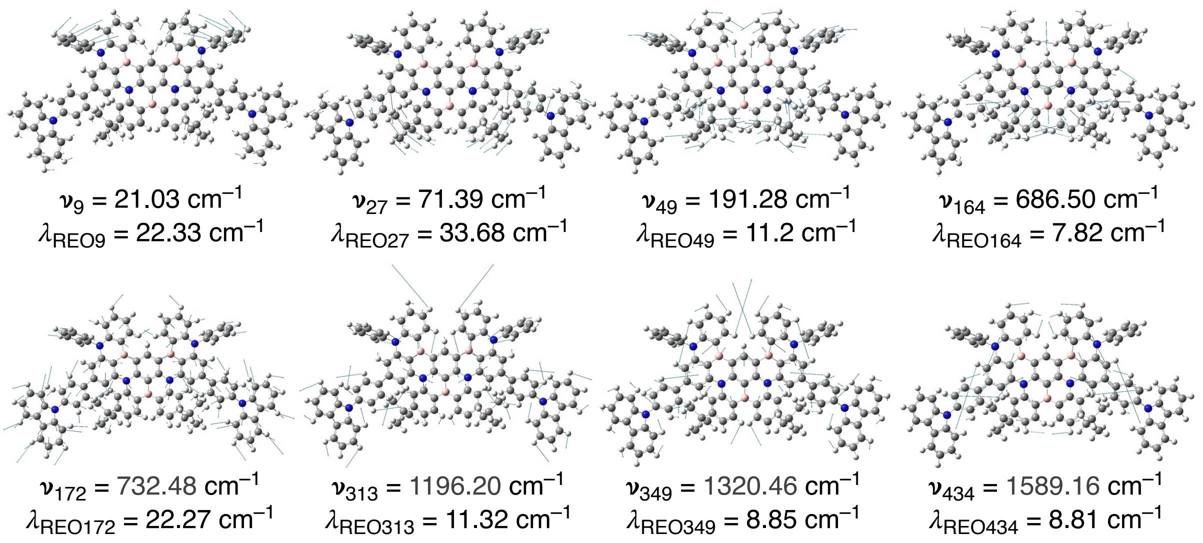

Figure S8. Vibrational modes according to the frequency and the numerical value of the reorganization energy ($\lambda_{REO\mathit{k}}$) of **ω-DABNA-4CzP**.

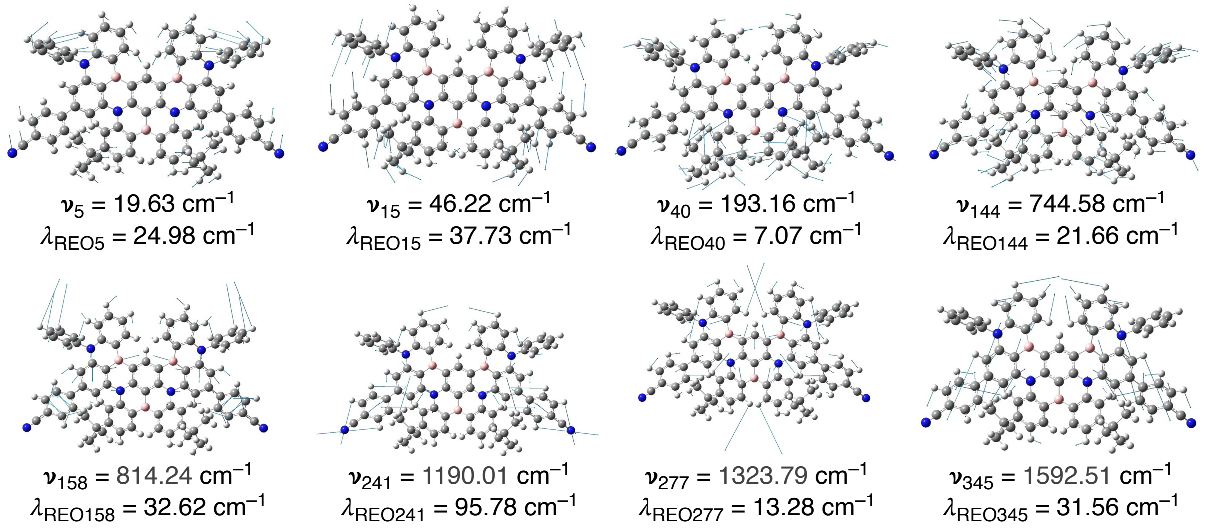

Figure S9. Vibrational modes according to the frequency and the numerical value of the reorganization energy ($\lambda_{REO\mathit{k}}$) of **ω-DABNA-4CNP**.

Synthesis of 3,11-di-*tert*-butyl-5,9-bis(3,5-dichlorophenyl)-5,9-dihydro-5,9-diaza-13b-boranaphtho[3,2,1-*de*]anthracene (a)

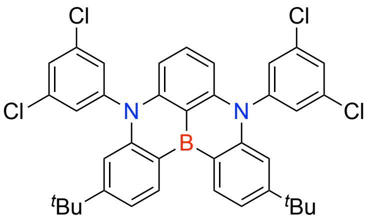

The title compound was prepared according to the reported procedure[26].

Synthesis of 5,5'-(3,11-di-tert-butyl-5,9-diaza-13b-boranaphtho[3,2,1-de]anthracene-5,9-diyl)bis(3-chloro-*N*,*N*-diphenylaniline) (b)

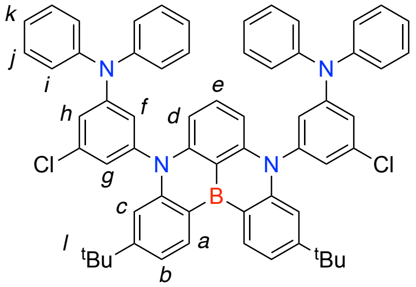

Palladium(II) acetate (71.8 mg, 0.32 mmol), dicyclohexyl(2',6'-dimethoxy-[1,1'-biphenyl]-2-yl)phosphine (0.263 g, 0.64 mmol), **a** (2.68 g, 4.0 mol), diphenylamine (1.39 g, 8.2 mmol) and sodium *tert*-butoxide (0.788 g, 8.2 mmol) were dissolved in toluene (40 mL) under a nitrogen atmosphere. After stirring at 70 °C for 16 h, the reaction mixture was allowed to cool to room temperature. After the reaction mixture was filtered with a pad of silica gel (eluent: toluene), the solvent was removed *in vacuo*. The crude product was purified by silica gel column chromatography (eluent: hexane/toluene = 4/1) to obtain the title compound (2.04 g, 54% yield, 96% pure on ^{1}H NMR analysis) as a yellow solid. IR (neat): cm^{-1} 3066, 2960, 2865, 1605, 1565, 1492, 1436, 1415, 1362, 1344, 1287, 1271, 1228, 1185, 1068, 1028, 992, 965, 940, 926, 897, 860, 818, 787, 752, 728, 692, 672, 658, 633, 599, 502, 466, 400; mp > 185.6 °C, ^{1}H NMR (495 MHz, $CDCl_3$) δ 1.36 (s, 18H, *l*), 6.33 (d, *J* = 8.2 Hz, 2H, *d*), 6.89 (d, *J* = 2.0 Hz, 2H, *c*), 6.95 (s, 2H, *h*), 6.98 (s, 2H, *g*), 7.06 (t, *J* = 7.4 Hz, 4H, *k*), 7.17 (d, *J* = 7.7 Hz, 8H, *i*), 7.25–7.29 (m, 10H, *f*, *j*), 7.32 (dd, *J* = 7.9, 1.7 Hz, 2H, *b*), 7.43 (t, *J* = 8.4 Hz, 1H, *e*), 8.81 (d, *J* = 8.2 Hz, 2H, *a*); ^{13}C NMR (125 MHz, $CDCl_3$) δ 31.3 (6C), 35.2 (2C), 105.1 (2C), 113.4 (2C), 117.2 (1C) 118.1 (2C), 121.6 (2C), 122.3 (2C), 122.9 (2C), 123.6 (2C), 124.3 (4C), 125.2 (8C), 129.8 (8C), 131.9 (1C), 134.9 (2C), 136.9 (2C), 144.1(2C), 146.5 (2C), 146.8 (4C), 147.2 (2C), 151.6 (2C), 154.2(2C); ^{11}B NMR (159 MHz, $CDCl_3$) δ 38.8; HRMS (MALDI-TOF/MS) *m/z* $[M]^+$ calcd. for $C_{62}H_{53}B_1Cl_2N_4$ 934.3740; observed 934.3753.

Synthesis of 3,23-di-*tert*-butyl-6,20-dichloro-8,18-di-phenyl-8*H*,18*H*-4b,8,18,21b-tetraaza-12b,13b,25b-triborabenzo[3,4]phenanthro[2,1,10,9-*qrst*]dinaphtho[3,2,1-*de*:1',2',3'-*jk*]pentacene (ω-DABNA-Cl)

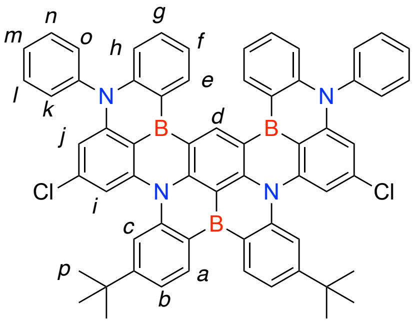

Compound **b** (93.5 mg, 0.10 mmol) and boron triiodide (157 mg, 0.40 mmol) were dissolved in chlorobenzene (1.0 mL) under a nitrogen atmosphere. After stirring at 90 °C for 18 h, the reaction mixture was diluted with dichloromethane (2.0 mL) and quenched by phosphate buffer solution (pH 6.8, 50 mL) at 0 °C. The aqueous layer was separated and extracted with dichloromethane (30 mL, three times). After the combined organic layer was condensed *in vacuo*, the crude product was purified by silica gel column chromatography (eluent: hexane/toluene = 3/1) to obtain the title compound (38 mg, 40% yield, 92% pure on HPLC analysis) as an orange solid. IR (neat): cm^{-1} 2958, 2866, 1724, 1607, 1579, 1564, 1546, 1491, 1476, 1453, 1436, 1400, 1383, 1362, 1340, 1320, 1257, 1220, 1182, 1164, 1128, 1101, 1071, 1033, 965, 945, 936, 916, 848, 824, 801, 755, 748, 740, 730, 701, 665, 633, 626, 608, 592, 535; mp > 300 °C, ^{1}H NMR (495 MHz, $CDCl_3$) δ 1.40 (s, 18H, *p*), 6.42 (d, J = 1.4 Hz, 2H, *j*), 6.87 (d, J = 8.5 Hz, 2H, *h*), 7.30 (t, J = 7.2 Hz, 2H, *f*), 7.40 (d, J = 6.8 Hz, 2H, *k*), 7.54 (t, J = 8.5 Hz, 2H, *g*), 7.57–7.61 (m, 4H, *b*, *o*), 7.69 (t, J = 7.5 Hz, 2H, *m*), 7.75–7.80 (m, 6H, *i*, *l*, *n*), 8.40 (s, 2H, *c*), 8.68 (d, J = 7.9 Hz, 2H, *a*), 8.83 (d, J = 7.7 Hz, 2H, *e*), 10.28 (s, 1H, *d*); ^{13}C NMR (125 MHz, $CDCl_3$) δ 31.2 (6C), 35.5 (2C), 109.1 (2C), 112.9 (2C), 117.5 (2C), 119.7 (2C), 119.8 (2C), 121.0 (2C), 121.2 (2C), 121.7 (2C), 124.2 (2C), 127.6 (2C), 129.3 (2C), 130.2 (2C), 130.9 (2C), 131.1 (2C), 131.7 (2C), 131.8 (2C), 134.4 (2C), 135.6 (2C), 136.8 (2C), 141.6 (2C), 146.7 (2C), 147.1 (2C), 147.2 (2C), 147.9 (2C), 149.1 (2C), 149.3 (2C), 153.5 (2C);^{11}B NMR (159 MHz, $CDCl_3$) δ 41.7; HRMS (MALDI-TOF/MS) *m/z* $[M]^+$ calcd. for $C_{62}H_{47}B_3Cl_2N_4$ 950.3457; observed 950.3488.

Synthesis of 3,23-di-*tert*-butyl-bis[4-(*tert*-butyl)phenyl]-8,18-di-phenyl-8*H*,18*H*-4b,8,18,21b-tetraaza-12b,13b,25b-triborabenzo[3,4]phenanthro[2,1,10,9-*qrst*]dinaphtho[3,2,1-*de*:1',2',3'-*jk*]pentacene (ω-DABNA-4TBP)

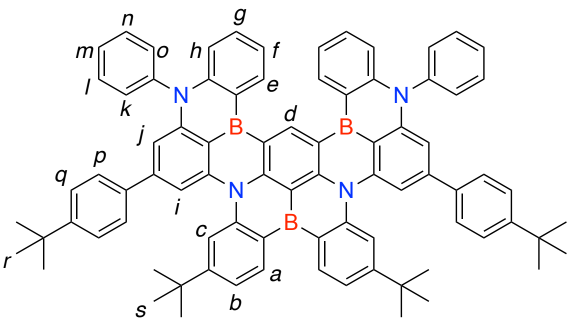

Dichlorobis(di-*tert*-butyl[*p*-dimethylaminophenyl]phosphino)palladium(II) (14.5 mg, 0.020 mmol), **ω-DABNA-Cl** (45.6 mg, 0.05 mmol), 4-*tert*-butylphenylboronic acid (54.1 mg, 0.30 mmol), and tripotassium phosphate (64.0 mg, 0.30 mmol) were dissolved in *N*–methylpyrrolidone (0.70 mL) under a nitrogen atmosphere. After stirring at

100 °C for 1 h, the reaction mixture was diluted with toluene (10 mL) and quenched by adding water (200 mL) at 0 °C. The aqueous layer was separated and extracted with toluene (30 mL, three times). After the combined organic layer was removed *in vacuo*. The crude product was purified by silica gel column chromatography (eluent: hexane/dichloromethane = 1/2) to obtain the title compound (30 mg, 52% yield, 95% pure on HPLC analysis) as an orange solid. Further purification for device fabrication is performed by washing with toluene and acetonitrile. IR (neat): cm^{-1} 2961, 2864, 1726, 1608, 1597, 1567, 1558, 1542, 1495, 1436, 1386, 1362, 1323, 1310, 1261, 1237, 1217, 1182, 1161, 1112, 1098, 1082, 1069, 1016, 1003, 985, 965, 867, 824, 801, 755, 750, 730, 717, 701, 694, 665, 651, 635, 628, 601, 593, 572, 533, 515; mp > 300 °C, ^{1}H NMR (495 MHz, $CDCl_3$), 1.32 (s, 18H, *r*), 1.39 (s, 18H, *s*), 6.68 (s, 2H, *j*), 6.90 (d, J = 8.5 Hz, 2H, *h*), 7.29 (t, J = 7.2 Hz, 2H, *f*), 7.36 (d, J = 8.2 Hz, 4H, *q*), 7.42 (d, J = 8.2 Hz, 4H, *p*), 7.48–7.54 (m, 4H, *g*, *o*), 7.58 (d, J = 7.9 Hz, 2H, *b*), 7.66 (m, 4H, *m*, *k*), 7.77 (t, J = 7.7 Hz, 4H, *l*, *n*), 7.99 (s, 2H, *i*), 8.52 (s, 2H, *c*), 8.70 (d, J = 7.9 Hz, 2H, *a*), 8.86 (d, J = 7.7 Hz, 2H, *e*), 10.28 (s, 1H, *d*); ^{13}C NMR (125 MHz, $CDCl_3$) 31.4 (6C), 31.4 (6C), 34.6 (2C), 35.5 (2C), 107.9 (2C), 112.0 (2C), 117.4 (2C), 117.6 (1C), 119.5 (2C), 119.9 (2C), 120.7 (2C), 120.8 (2C), 121.5 (2C), 124.4 (2C), 125.6 (2C), 127.3 (4C), 127.7 (2C), 128.9 (2C), 130.4 (2C), 131.0 (2C), 131.2 (2C), 131.5 (2C+2C), 134.3 (2C), 135.7 (2C), 138.6 (2C), 142.2 (2C), 143.1 (2C), 146.3 (2C), 146.7 (2C), 147.7 (2C), 148.2 (2C), 149.0 (1C), 149.6 (2C), 150.8 (2C), 153.0 (2C); ^{11}B NMR (159 MHz, $CDCl_3$) δ 42.3; HRMS (MALDI-TOF/MS) *m/z* $[M]^+$ calcd. for $C_{82}H_{73}B_3N_4$ 1146.6114; observed 1146.6169.

Synthesis of 3,23-di-*tert*-butyl-bis[9H-carbazol-9-yl]phenyl-8,18-di-phenyl-8*H*,18*H*-4b,8,18,21b-tetraaza-12b,13b,25b-triborabenzo[3,4]phenanthro[2,1,10,9-*qrst*]dinaphtho[3,2,1-*de*:1',2',3'-*jk*]pentacene (ω-DABNA-4CzP)

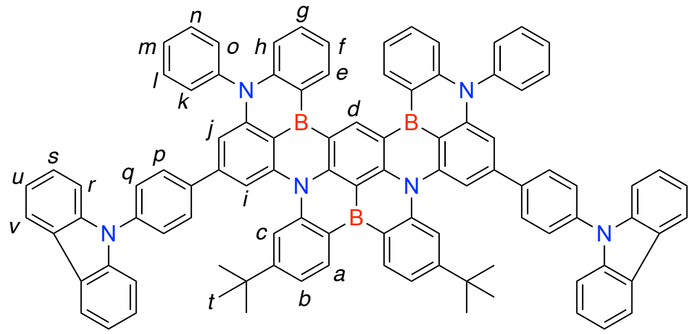

Dichlorobis(di-*tert*-butyl[*p*-dimethylaminophenyl]phosphino)palladium(II) (14.5 mg, 0.020 mmol), **ω-DABNA-Cl** (45.6 mg, 0.05 mmol), (4-(9*H*-carbazol-9-yl)phenyl)boronic acid (87.0 mg, 0.30 mmol), and tripotassium phosphate (63.5 mg, 0.30 mmol) were dissolved in *N*–methylpyrrolidone (0.70 mL) under a nitrogen atmosphere. After stirring at 100 °C for 1 h, the reaction mixture was diluted with toluene (10 mL) and quenched by adding water (200 mL) at 0 °C. The aqueous layer was separated and extracted with toluene (30 mL, three times). After the combined organic layer was removed *in vacuo*. The crude product was purified by silica gel column chromatography (eluent: hexane/dichloromethane = 4/1) to obtain the title compound (35 mg, 51% yield, 99% pure on HPLC analysis) as an orange solid. IR (neat): cm^{-1} 3046, 2957, 2858, 1724, 1597, 1561, 1546, 1519, 1491, 1479, 1450, 1438, 1386, 1359, 1333, 1314, 1231, 1214, 1181, 1165, 1118, 1096, 1071, 1016, 1003, 827, 800, 745, 721, 698, 652, 623, 612, 598, 588, 535, 434, 410; mp > 300 °C ^{1}H NMR (495 MHz, $CDCl_3$) δ 1.41 (s, 18H, *t*),

6.80 (s, 2H, *j*), 6.96 (d, *J* = 8.5 Hz, 2H, *h*), 7.29–7.32 (m, 4H, *u*), 7.35 (t, *J* = 7.2 Hz, 2H, *f*), 7.41–7.44 (m, 8H, *s*, *r*), 7.54–7.60 (m, 8H, *g*, *q*, *m*), 7.63 (dd, *J* = 8.1, 1.3 Hz, 2H, *b*), 7.71–7.74 (m, 8H, *k*, *o*, *p*), 7.79–7.84 (m, 4H, *l*, *n*), 8.13 (s, 2H, *i*), 8.16 (d, *J* = 7.9 Hz, 4H, *v*), 8.60 (s, 2H, *c*), 8.76 (d, *J* = 7.9 Hz, 2H, *a*), 8.93 (d, *J* = 6.5 Hz, 2H, *e*) 10.37 (s, 1H, *d*); ^{13}C NMR (125 MHz, CDCl$_3$) 31.4 (6C), 35.5 (2C), 107.8 (2C), 109.9 (4C), 112.1 (2C), 117.5 (2C), 117.7 (1C), 119.6 (2C), 120.1 (4C+2C), 120.4 (4C), 121.0 (2C+2C), 121.7 (2C), 123.6 (4C), 124.4 (2C), 126.1 (4C), 127.3 (2C+2C), 127.8 (2C), 128.9 (2C), 129.2 (2C), 129.3 (2C+2C), 131.2 (4C), 131.8 (4C), 134.5 (2C), 135.8 (2C), 137.3 (2C), 140.6 (2C), 140.9 (4C), 142.2 (2C), 142.3 (2C), 146.6 (2C), 147.0 (2C), 147.7 (2C), 148.3 (2C), 149.2 (1C), 149.7 (2C), 153.2 (2C); ^{11}B NMR (159 MHz, CDCl$_3$) δ 40.7; HRMS (MALDI-TOF/MS) *m/z* [M]$^+$ calcd. for $C_{98}H_{71}B_3N_6$ 1364.6019; observed 1364.6034.

Synthesis of 3,23-di-*tert*-butyl-bis[4-cyanophenyl]-8,18-di-phenyl-8*H*,18*H*-4b,8,18,21b-tetraaza-12b,13b,25b-triborabenzo[3,4]phenanthro[2,1,10,9-*qrst*]dinaphtho[3,2,1-*de*:1',2',3'-*jk*]pentacene (ω-DABNA-4CNP)

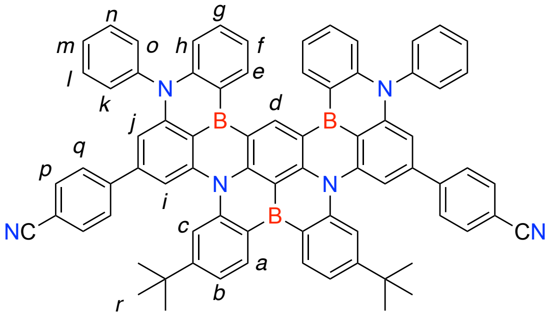

Dichlorobis(di-*tert*-butyl[*p*-dimethylaminophenyl]phosphino)palladium(II) (14.0 mg, 0.020 mmol), **ω-DABNA-Cl** (45.6 mg, 0.05 mmol), (4-cyanophenyl)boronic acid (45.0 mg, 0.30 mmol), and tripotassium phosphate (63.7 mg, 0.30 mmol) were dissolved in *N*–methylpyrrolidone (0.70 mL) under a nitrogen atmosphere. After stirring at 100 °C for 1 h, the reaction mixture was diluted with toluene (10 mL) and quenched by adding water (200 mL) at 0 °C. The aqueous layer was separated and extracted with toluene (30 mL, three times). After the combined organic layer was removed *in vacuo*. The crude product was purified by silica gel column chromatography (eluent: hexane/dichloromethane = 1/2) to obtain the title compound (37 mg, 68% yield, 95% pure on HPLC analysis) as an orange solid. Further purification for device fabrication is performed by washing with acetonitrile. IR (neat): cm^{-1} 2961, 2227, 1595, 1565, 1545, 1509, 1491, 1476, 1460, 1438, 1410, 1386, 1362, 1317, 1258, 1234, 1215, 1181, 1162, 1084, 1016, 963, 934, 866, 797, 755, 748, 727, 699, 665, 651, 628, 598, 591, 533, 512, 484, 470, 451, 431, 401; mp > 300 °C ^{1}H NMR (495 MHz, CDCl$_3$) δ 1.34 (s, 18H, *r*), 6.63 (s, 2H, *j*), 6.93 (d, *J* = 8.8 Hz, 2H, *h*), 7.33 (t, *J* = 7.2 Hz, 2H, *f*), 7.47 (d, *J* = 5.1 Hz, 2H, *k*), 7.54–7.57 (m, 6H, *g*, *p*), 7.60 (d, *J* = 7.9, 2H, *b*), 7.64 (d, *J* = 8.2, 4H, *q*), 7.68–7.71 (m, 4H, *m*, *o*), 7.76–7.86 (m, 4H, *l*, *n*), 7.98 (s, 2H, *i*), 8.45 (s, 2H, *c*), 8.72 (d, *J* = 7.9 Hz, 2H, *a*), 8.87 (d, *J* = 7.4 Hz, 2H, *e*) 10.32 (s, 1H, *d*); ^{13}C NMR (125 MHz, CDCl$_3$) 31.3 (6C), 35.5 (2C), 108.0 (2C), 111.3 (2C), 111.7 (2C), 117.5 (2C+1C), 118.9 (2C), 119.4 (2C), 120.1 (2C), 121.2 (2C), 121.4 (2C), 121.8 (2C), 124.3 (2C), 127.7 (2C), 128.1 (4C), 129.2 (2C), 130.3 (2C), 131.0 (2C), 131.1 (2C), 131.7 (2C), 131.9 (2C), 132.6 (4C), 134.5 (2C), 135.7 (2C), 141.1 (2C), 141.9 (2C), 146.0 (2C), 146.5 (2C), 147.0 (2C), 147.5 (2C), 148.2 (2C), 149.2 (1C), 149.6 (2C), 153.3 (2C); ^{11}B NMR (159 MHz, CDCl$_3$) δ 44.0; HRMS (MALDI-TOF/MS) *m/z* [M]$^+$ calcd. for $C_{76}H_{55}B_3N_6$ 1084.4767; observed 1084.4766.

Estimation of the rate constant for reverse intersystem crossing. Rate constants (k_F, k_{IC}, k_{ISC}, and k_{RISC}) were determined from the measurements of quantum yields (Φ_F and Φ_{TADF}) and lifetimes (τ_F, τ_{TADF}) and of the prompt (fluorescence) and delayed (TADF) components according to Adachi's method (equations 1–2[27], 3–5[28], and 6[29]).

ω-DABNA-4TBP

$\Phi = 0.939$ (excitation = 365 nm)

$\Phi_F = 0.898$

$\Phi_{TADF} = 0.041$

$\tau_F = 6.18$ ns

$\tau_{TADF} = 18.4$ μs

$k_p = 1.62 \times 10^8$ s^{-1}	$k_p = 1/\tau_F$	(1)
$k_d = 5.44 \times 10^4$ s^{-1}	$k_d = 1/\tau_{TADF}$	(2)
$k_F = 1.45 \times 10^8$ s^{-1}	$k_F = \Phi_F/\tau_F$	(3)
$k_{IC} = 9.43 \times 10^6$ s^{-1}	$\Phi = k_F/(k_F + k_{IC})$	(4)
$k_{ISC} = 7.08 \times 10^6$ s^{-1}	$\Phi_F = k_F/(k_F + k_{IC} + k_{RISC})$	(5)
$k_{RISC} = 5.69 \times 10^4$ s^{-1}	$k_{RISC} = k_p k_d/(k_p - k_{ISC})$	(6)

ω-DABNA-4CzP

$\Phi = 0.930$ (excitation = 365 nm)

$\Phi_F = 0.888$

$\Phi_{TADF} = 0.042$

$\tau_F = 5.91$ ns

$\tau_{TADF} = 14.3$ μs

$k_p = 1.69 \times 10^8$ s^{-1}	$k_p = 1/\tau_F$	(1)
$k_d = 7.01 \times 10^4$ s^{-1}	$k_d = 1/\tau_{TADF}$	(2)
$k_F = 1.50 \times 10^8$ s^{-1}	$k_F = \Phi_F/\tau_F$	(3)
$k_{IC} = 1.13 \times 10^7$ s^{-1}	$\Phi = k_F/(k_F + k_{IC})$	(4)
$k_{ISC} = 7.57 \times 10^6$ s^{-1}	$\Phi_F = k_F/(k_F + k_{IC} + k_{RISC})$	(5)
$k_{RISC} = 7.34 \times 10^4$ s^{-1}	$k_{RISC} = k_p k_d/(k_p - k_{ISC})$	(6)

---

(27) K. Masui, H. Nakanotani, C. Adachi, *Org. Electron.* **2013**, *14*, 2721.

(28) Q. Zhang, H. Kuwabara, W. J. Potscavage, S. Huang, Y. Hatae, T. Shibata, C. Adachi, *J. Am. Chem. Soc.* **2014**, *136*, 18070.

(29) H. Kaji, H. Suzuki, T. Fukushima, K. Shizu, K. Katsuaki, S. Kubo, T. Komino, H. Oiwa, F. Suzuki, A. Wakamiya, Y. Murata, C. Adachi, *Nat. Commun.* **2015**, *6*, 8476.

ω-DABNA-4CNP

$\Phi = 0.936$ (excitation = 365 nm)

$\Phi_F = 0.887$

$\Phi_{TADF} = 0.049$

$\tau_F = 6.39$ ns

$\tau_{TADF} = 20.9$ μs

$k_p = 1.56 \times 10^8$ s^{-1}	$k_p = 1/\tau_F$	(1)
$k_d = 4.79 \times 10^4$ s^{-1}	$k_d = 1/\tau_{TADF}$	(2)
$k_F = 1.38 \times 10^8$ s^{-1}	$k_F = \Phi_F/\tau_F$	(3)
$k_{IC} = 9.49 \times 10^6$ s^{-1}	$\Phi = k_F/(k_F + k_{IC})$	(4)
$k_{ISC} = 8.19 \times 10^6$ s^{-1}	$\Phi_F = k_F/(k_F + k_{IC} + k_{RISC})$	(5)
$k_{RISC} = 5.05 \times 10^4$ s^{-1}	$k_{RISC} = k_p k_d/(k_p - k_{ISC})$	(6)

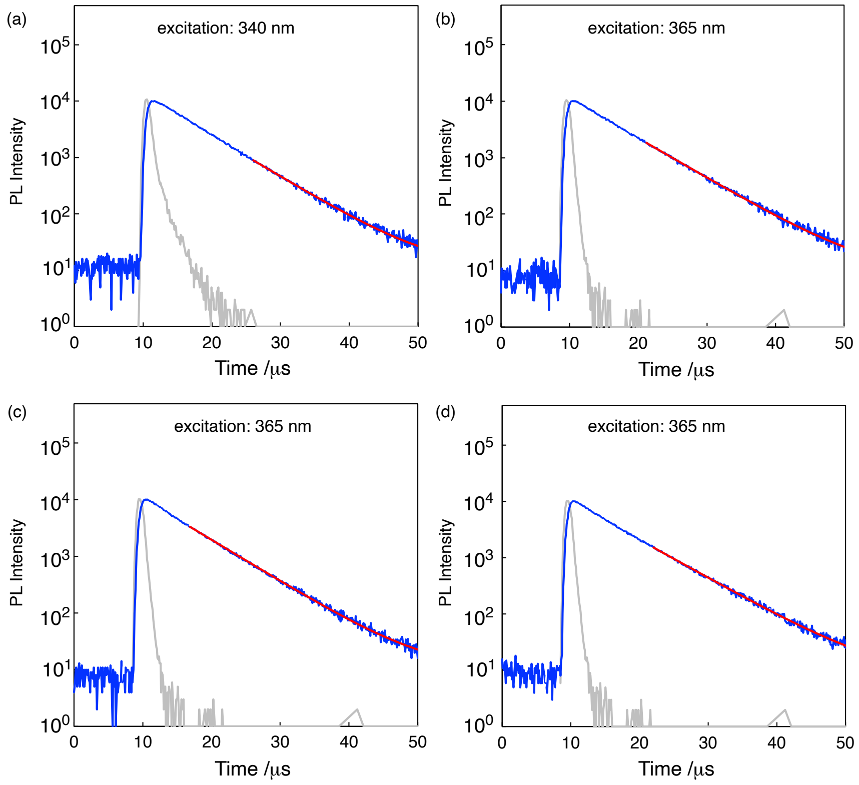

Figure S10. Transient decay spectra of a) **ω-DABNA**[26], b) **ω-DABNA-4TBP**, c) **ω-DABNA-4CzP**, and d) **ω-DABNA-4CNP** in PMMA 1 wt.%-doped film at 300 K. The gray curve represents an instrument response function (IRF). The red curve represents the single exponential fitting data (background = 8–11).

Table S2. Summary of Photophysical Data of **ω-DABNA, ω-DABNA-4TBP**, **ω-DABNA-4CzP**, and **ω-DABNA-4CNP** in 1 wt.% PMMA Film.

Compound	λ_{abs} [a)] [nm]	λ_{em} [b)] [nm]	FWHM[c)] [nm]	Φ [d)] [%]	λ_{em} [e)] [nm]	ΔE_S [f)] [eV]	ΔE_T [f)] [eV]	ΔE_{ST} [g)] [meV]
**ω-DABNA**[h)]	495	509	22	87	514/517	2.41	2.40	13
**ω-DABNA -4TBP**[i)]	506	519	23	94	524/528	2.36	2.35	15
**ω-DABNA -4CzP**[i)]	509	521	23	93	527/529	2.35	2.34	15
**ω-DABNA -4CNP**[i)]	513	528	24	94	534/536	2.32	2.31	10

[a)]Maximum absorption wavelength. [b)]Maximum fluorescence wavelength. [c)]Full width at half maximum. [d)]Absolute photoluminescence quantum yield. [e)]Maximum fluorescence phosphorescence wavelength. [f)]Singlet and triplet energies estimated from peak top emission at 77 K. [g)]Energy gap between Singlet and Triplet states. [h)]ref [26]. [i)]Excited at 405 nm. [j)]Excited at 470 nm.

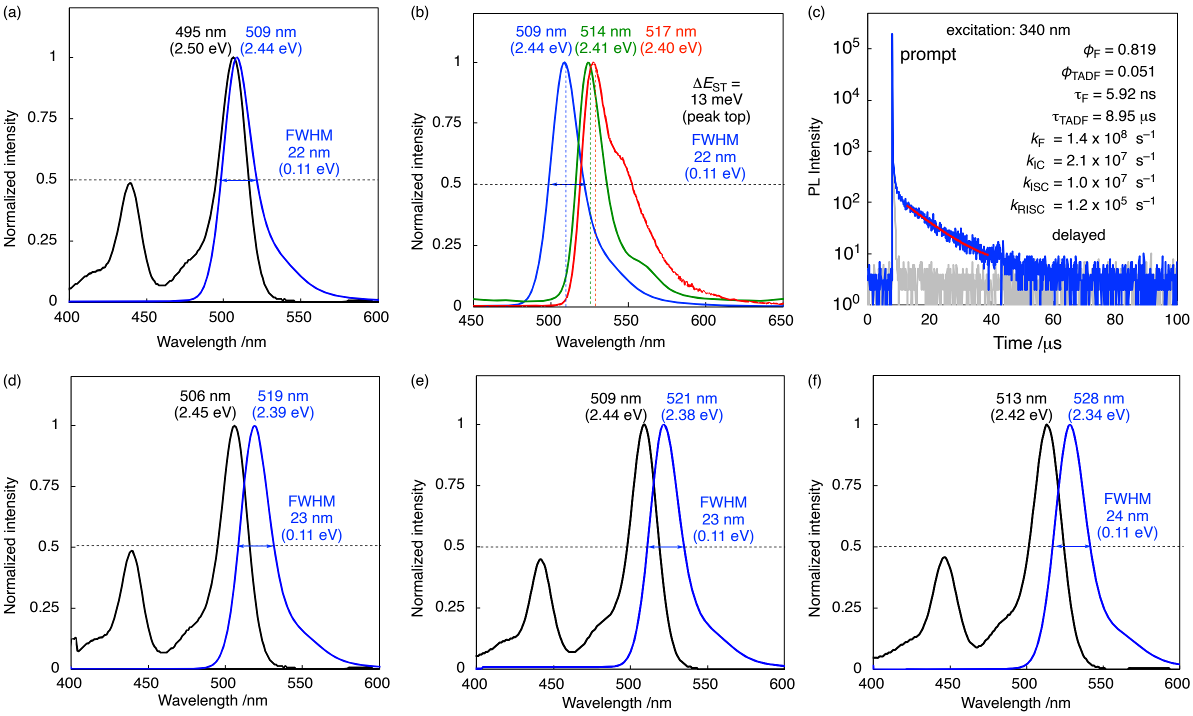

Figure S11. Photophysical properties of a,b,c) **ω-DABNA**[26],d) **ω-DABNA-4TBP**, e) **ω-DABNA-4CzP**, and f) **ω-DABNA-4CNP** in PMMA 1 wt.%-doped film. a,d,e,f) Normalized absorption (black) and fluorescence (blue). b) Fluorescence spectra at 300 K (blue) and 77 K with (green) and without (red) a delay time of 25 ms. b) Transient photoluminescence decay curves at 300 K and their relevant parameters, and the gray curve represents an instrument response function (IRF). The red curve represents the single exponential fitting data (background = 5). Φ_F and Φ_{TADF}, quantum yields of the fluorescent and TADF components, respectively; τ_F and τ_{TADF}, lifetimes of the fluorescent and TADF components, respectively. Rate constants from fluorescence (k_F), internal conversion from S_1 to S_0 (k_{IC}), intersystem crossing from S_1 to T_1 (k_{ISC}), and revers intersystem crossing from T_1 to S_1 (k_{RISC}) calculated from Φ, Φ_F, Φ_{TADF}, τ_F, and τ_{TADF}, according to the reported methods.[27–29]

Electrochemical Characterization. Cyclic and differential pulse voltammetry measurements were performed with an ALS600E electrochemical analyzer (BAS). The cell consisted of a glassy carbon electrode, a Pt wire counter electrode, and a standard Ag/AgCl reference electrode. The measurements were carried out under a N_2 atmosphere using a 1 mM sample solution in DCM for cyclic voltammetry (CV) and DMF for differential pulse voltammetry (DPV), respectively, containing 0.1 M tetrabutylammonium hexafluorophosphate as the supporting electrolyte. All potentials are referenced against the ferrocene/ferricenium (Fc/Fc^+) couple. The scan rate is 0.2 V s^{-1}.

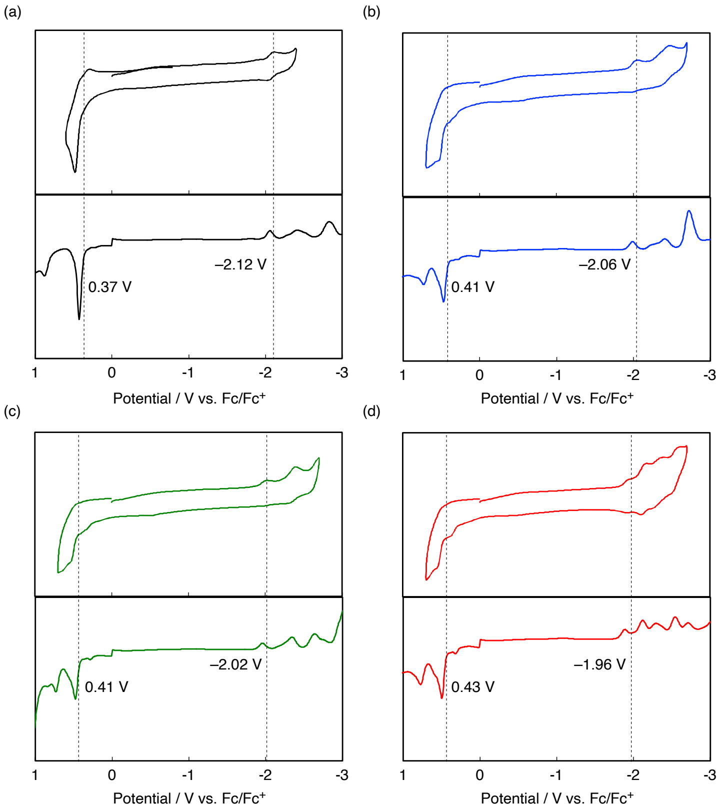

Figure S12. Cyclic and differential pulse voltammetry of (a) **ω-DABNA**, (b) **ω-DABNA-4TBP**, (c) **ω-DABNA-4CzP**, and (d) **ω-DABNA-4CNP** measured at a scan rate of 0.2 V s^{-1} in DCM (1 mM) for CV and DMF (1 mM) for DPV at room temperature using $n$$Bu_4NClO_4$ (0.1 M) as the supporting electrolyte. All potentials are referenced against the ferrocene/ferricenium (Fc/Fc^+) couple.

Table S3. The redox potentials of **ω-DABNA, ω-DABNA-4TBP**, **ω-DABNA-4CzP,** and **ω-DABNA-4CNP**.

compounds	Oxidation potential (eV) (vs. Fc/Fc^+)	Reduction potential (eV) (vs. Fc/Fc^+)
**ω-DABNA**	–5.17	–2.68
**ω-DABNA-4TBP**	–5.21	–2.75
**ω-DABNA-4CzP**	–5.21	–2.78
**ω-DABNA-4CNP**	–5.23	–2.85

Crystallographic Data Collection and Structure Determination. X-ray Crystallographic Analysis of compound ω-DABNA-Cl and ω-DABNA-4TBP. Pure orange single crystals were grown by slow diffusion of acetonitrile into solutions of **ω-DABNA-Cl** and **ω-DABNA-4TBP** in toluene. Intensity data were collected at 100 K on a Rigaku XtaLAB Synergy-R diffractometer equipped with a PhotonJet-R generator and a HyPix-6000HE photon counting detector with Cu*K*α radiation (λ = 1.54184 Å). The reflection data were collected, integrated, scaled, and averaged by using Rigaku CrysAlisPro software. Empirical absorption correction using spherical harmonics, implemented in SCALE3 ABSPACK scaling algorithm (CrysAlisPro, Rigaku Oxford Diffracion). The structures were solved by the direct method (SHELXT 2018/2)[30] and refined by full-matrix least square method on F^2 (SHELXT-2019/2)[31]. All hydrogen atoms were placed using AFIX instructions, while all other atoms were refined anisotropically.

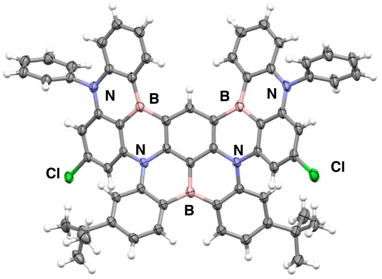

Formula	$C_{62}H_{47}B_3Cl_2N_4$, C_2H_3N
Formula Weight	992.42
Temperature, K	100
Wavelength, Å	1.54184
Crystal System	triclinic
Space Group	*P*–1
a, Å	10.4868(2)
b, Å	10.6871(2)
c, Å	24.2067(3)
α, °	79.0800(10)
β, °	80.0730(10)
γ, °	81.609(2)
Volume, Å^3	2605.87(8)
Z	2

Density$_{calcd}$, g . cm^{-3}	1.265
Abs. Coefficient, cm^{-1}	1.478
F(000)	1036
Crystal Size, mm^3	0.060, 0.020, 0.020
$2\theta_{min}$, $2\theta_{max}$, deg	7.5, 149.0
Index Ranges	–13≤h≤13
	–13≤k≤13
	–30≤l≤23
Reflections (unique)	10631
Reflections (I>2.0s(I))	8402
Parameters	705
GOF on F^2	1.052
R_1 (I>2.0σ(I))	0.0583
R, wR_2 (all data)	0.0724, 0.1711
Largest diff peak and hole, e, Å^{-3}	0.598, –0.529

Figure S13. X-ray crystal structure of **ω-DABNA-Cl** (left), and crystal data and structure refinement (right). Thermal ellipsoids are shown at 50% probability.

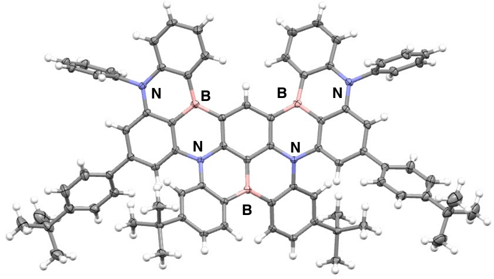

Formula	$C_{82}H_{73}B_3N_4$, C_7H_8, C_2H_3N
Formula Weight	1321.11
Temperature, K	100
Wavelength, Å	1.54184
Crystal System	Monoclinic
Space Group	*C*2/*c*
a, Å	25.2228(5)
b, Å	30.1157(6)
c, Å	9.7925(2)
α, °	90
β, °	90.098(2)
γ, °	90
Volume, Å^3	7427.5(3)
Z	4

Density$_{calcd}$, g . cm^{-3}	1.181
Abs. Coefficient, cm^{-1}	0.516
F(000)	2808
Crystal Size, mm^3	0.12, 0.05, 0.05
$2\theta_{min}$, $2\theta_{max}$, deg	4.6, 149.0
Index Ranges	–30≤h≤31
	–28≤k≤37
	–12≤l≤12
Reflections (unique)	7580
Reflections (I>2.0s(I))	5162
Parameters	471
GOF on F^2	1.049
R_1 (I>2.0σ(I))	0.0640
R, wR_2 (all data)	0.0983, 0.1852
Largest diff peak and hole, e, Å^{-3}	0.636, –0.412

Figure S14. X-ray crystal structure of **ω-DABNA-4TBP** (left), and crystal data and structure refinement (right). Thermal ellipsoids are shown at 50% probability.

(30) Sheldrick, G. M. *Acta Cryst*. **2015**, *A71*, 3.

(31) Sheldrick, G. M. *Acta Cryst*. **2015**, *C71*, 3.

Device fabrication and measurement of electroluminescence characteristics. Organic light emitting diodes (OLEDs) were fabricated on glass substrates coated with a patterned transparent ITO conductive layer. The substrates were treated with 300W oxygen plasma. OLED employing **ω-DABNA**, **ω-DABNA-4TBP**, **ω-DABNA-4CzP**, and **ω-DABNA-4CNP** as an emitter with the following structure was fabricated: indium tin oxide (ITO, 50 nm); *N*,*N*'-di(1-naphthyl)-*N*,*N*'-diphenyl-(1,1'-biphenyl)-4,4'-diamine (NPD, 40 nm); tris(4-carbazolyl-9-ylphenyl)amine (TCTA, 15 nm); 1,3-bis(*N*- carbazolyl)benzene (mCP, 15 nm); 0.5 wt% emitter and 99.5 wt% of DOBNA-Ph (30 nm); 3,4-di(9H-carbazol-9- yl)benzonitrile (3,4-2CzBN, 10 nm); 2,7-bis(2,2'-bipyridine-5-yl)triphenylene (BPy-TP2, 25 nm); LiF (1 nm); Al (100 nm). The emitter molecule was not sublimated before the OLED fabrication in this study. The pressure during the vacuum evaporation was 5.0×10^{-4} Pa, and the film thickness was controlled using a calibrated quartz crystal microbalance during deposition. After the deposition of all layers, the OLED test modules were encapsulated with a capping glass in an evaporation chamber filled with nitrogen. The OLED characteristics of all fabricated devices were evaluated at room temperature (298 K) in an air atmosphere using a voltage–current–luminance measuring system, comprising a source meter (Keithley 2400) and spectral radiance meter (Topcon SR-3AR). The EQE was calculated using the EL spectrum, assuming the light-emitting surface to be a perfect diffusion surface and adding up all radiance elements from every angle and inputting them into the formula for obtaining EQE.

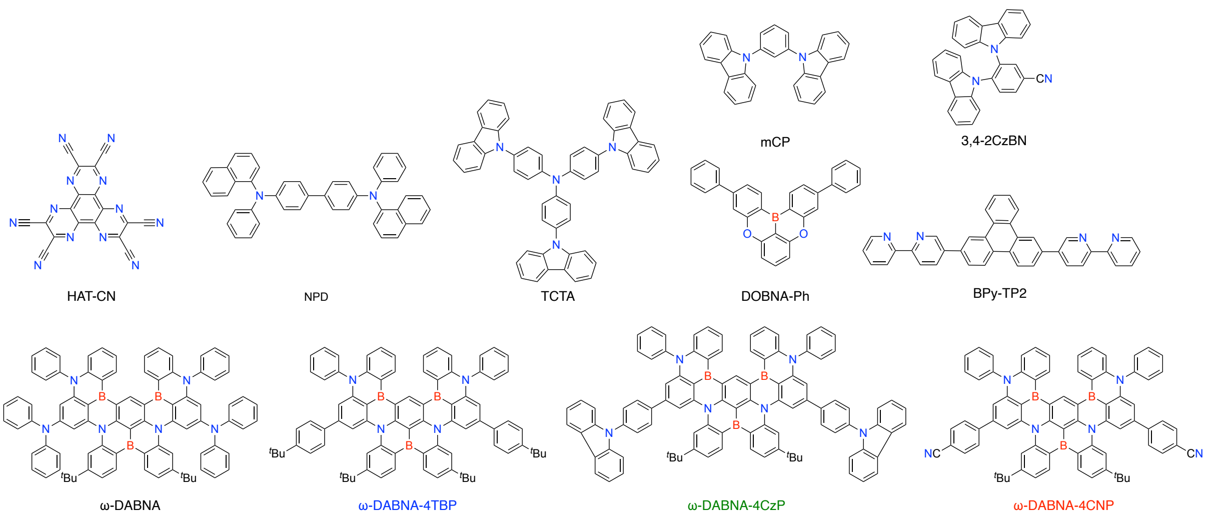

Figure S15. Molecular structures of OLED materials employed in this study.

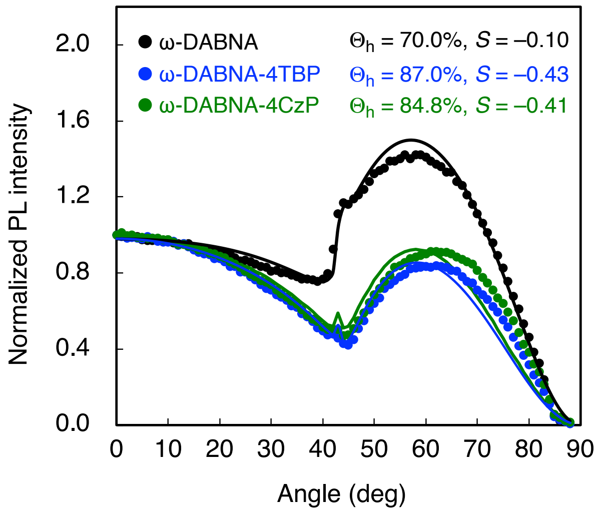

Figure S16. Angular dependent PL measurement of **ω-DABNA**, **ω-DABNA-4TBP**, and **ω-DABNA-4CzP** doped in films of DOBNA-Ph.

Table S4. Summary of OLED Performance Employing Green MR-TADF Emitter.

Emitter	λ_{EL} [nm][a]	FWHM [nm][b]	V_{on} [eV][c]	EQE_{max} [%][d]	CIE [x, y][e]	EQE_{1000} [eV][f]	roll-off [%][g]	CE_{max} [cd A^{-1}][h]	PE_{max} [lm W^{-1}][i]	PE_{1000} [lm W^{-1}][j]	Ref
**ω-DABNA -4TBP**	526	25	3.0	34.7	0.20, 0.75	32.6	6.1	142.2	150.0	95.6	This work
**ω-DABNA -4CzP**	528	25	3.0	33.4	0.22, 0.73	30.5	8.7	140.0	147.0	90.2	This work
**ω-DABNA -4CNP**	537	25	3.0	32.8	0.28, 0.70	28.6	12.8	145.8	153.8	91.1	This work
**ω-DABNA**	510	25	3.0	32.7	0.12, 0.72	29.5	9.8	104.2	110.3	66.2	This work
**CzB2-M/P**	497	29	3.4	26.7	0.12, 0.57	18	32.6	67.8	62.6	27.8	32
**ν-DABNA -CN-Me**	504	23	3.0	31.9	0.13, 0.65	28.5	10.7	88.9	93.6	56.8	33
**ω-DABNA**	512	25	3.2	31.1	0.13, 0.73	29.4	5.5	101.8	88.8	67.0	26
**ω-DABNA**	512	25	3.0	30.8	0.13, 0.73	29.2	5.2	101.9	106.2	-	34
**ω-DABNA -M**	515	25	3.1	32.7	0.15, 0.74	27.4	16.2	114.1	114.5	-	34
**ω-DABNA -PH**	521	30	3.2	31.8	0.19, 0.74	27.4	13.8	123.6	124.8	-	34
**TCz-B**	515	30	4.2	29.2	0.16, 0.71	9.4	67.8	100.7	72.4	-	35
**DBNO**	504	24	3.1	35.9	0.18, 0.60	5.9	83.6	94.1	98.5	10.0	36
**DBON**	510	29	3.6	26.7	0.17, 0.68	12	55.1	94.1	75.8	22.8	37
**tCzphB-Fl**	535	26	3.0	26.2	0.26, 0.72	8	69.5	-	-	-	38
**tCzphB-Ph**	527	24	2.8	29.3	0.21, 0.75	9	69.3	-	-	-	38

**DBTN-2**	520	29	2.8	35.2	0.19, 0.74	20.4	42.0	132.9	130.4	56.0	39
**DBT-DBN**	520	24	3.2	31.3	0.22, 0.70	8	74.4	116.2	93.6	20.0	40
**tPhCzPh3 Si**	512	28	3.6	34.6	0.14, 0.70	16.1	53.5	112.3	80.1	28.3	41
**2PyBN**	505	26	2.4	37.1	0.12, 0.65	18	51.5	106.7	139.6	50.0	42
**SS-DAO**	518	24	3.7	31.6	0.18, 0.73	13.8	56.3	107.5	83.6	25.3	43
**BpIC-DPA**	536	27	2.4	16.8	0.30, 0.67	5.6	66.7	62.4	81.7	-	44
**BNDCN**	507	30	3.9	32.3	0.13, 0.65	12.2	62.2	104.3	76.2	21.3	45
**m-CzDAz -BNCz**	508	29	3.0	36.6	0.12, 0.67	18.5	49.5	104	108.6	30.0	46
**PhCzBN-PO**	513	29	2.4	25.6	0.22, 0.68	13.2	48.4	90.8	118.9	30.0	47
**QB-DPA**	502	17	2.5	36	0.10, 0.61	30.4	15.6	78.6	89.1	50.0	48
**QB-PXZ**	516	20	2.4	36.6	0.18, 0.74	31.8	13.1	134.8	151.3	90.0	49
**5Cz-BN**	502	29	3.0	32.2	0.31, 0.62	12.1	62.4	-	-	-	49
**PhQA1**	534	28	3.3	7.02	0.33, 0.65	4.3	38.7	27.76	26.42	12.0	50
**PhQA3**	540	28	3.3	6.4	0.37, 0.62	4.77	25.5	25.23	24.02	14.5	50
**pTIQA**	526	28	2.8	21.1	0.30, 0.64	20.6	2.4	66.1	59.5	30.0	51
**CNBN**	506	16	2.4	34.4	0.12, 0.68	23.3	32.3	98.3	128.7	80.0	52
**MCNBN**	517	17	2.4	30.8	0.17, 0.74	20.4	33.8	111.4	145.8	80.0	52
**PCNBN**	508	20	2.4	29.1	0.14, 0.67	24.4	16.2	85.2	95.6	60.0	52
**PMCNBN**	519	22	2.4	27.2	0.19, 0.73	20.3	25.4	99.6	111.8	60.0	52
**ω`-DABNA**	517	25	2.4	32.3	0.16, 0.73	26.1	19.2	118	154.5	-	53
**ω`-DABNA -D**	519	25	2.4	34.6	0.16, 0.75	30.7	11.3	128.5	155.5	-	53

[a]Maximum wavelength of EL spectrum. [b]Full width at half maximum. [c]Turn-on voltage at the luminescence of 1 cd m^{-2}. [d]Maximum External quantum efficiency. [e]CIE (x,y) coordinates.[f] External quantum efficiency at 1000 cd m^{-2}. [g]Efficiency roll-off at 1000 cd m^{-2} from EQE_{max}. [h]Maximum current efficiency [i]Maximum power efficiency. [j]Power efficiency at 1000 cd m^{-2}.

(32) S. Oda, W. Kumano, T. Hama, R. Kawasumi, K. Yoshiura, T. Hatakeyama, *Angew. Chem. Int. Ed.* **2021**, 60, 2882–2886.

(33) S. Oda, T. Sugitani, H. Tanaka, K. Tabata, R. Kawasumi, T. Hatakeyama, *Adv. Mater.* **2022**, 34, 2201778.

(34) Y.-T. Lee, C.-Y. Chan, N. Matsuno, S. Uemura, S. Oda, M. Kondo, R. W. Weerasinghe, Y. Hu, G. N. I. Lestanto, Y. Tsuchiya, Y. Li, T. Hatakeyama. C. Adachi, *Nat. Commun.* **2024** *15*, 3174.

(35) M. Yang, S. Shikita, H. Min, I. S. Park, H. Shibata, N. Amanokura, T. Yasuda, *Angew. Chem. Int. Ed.* **2021**, 60, 23142.

Hyperfluorescence (HF)-OLEDs were fabricated on glass substrates coated with a patterned transparent ITO conductive layer. The substrates were treated with 300W oxygen plasma. OLED employing **ω-DABNA, ω-DABNA-4TBP, ω-DABNA-4CzP,** and **ω-DABNA-4CNP** as an emitter with the following structure was fabricated: indium tin oxide (ITO, 50 nm); 1,4,5,8,9,11-hexaazatriphenylenehexacarbonitrile (HAT-CN, 5 nm); *N*,*N*'-di(1-naphthyl)-*N*,*N*'-diphenyl-(1,1'-biphenyl)-4,4'-diamine (NPD, 60 nm); 9-(3-(triphenylsilyl)phenyl)-9H-3,9'-bicarbazole (Si-CzCz, 10 nm); Si-CzCz: 4tCzBN-PhCN: emitter (30 nm, 79:20:1 wt%); 9,9'-(6-(3-

(36) X. Cai, J. Xue, C. Li, B. Liang, A. Ying, Y. Tan, S. Gong, Y. Wang, *Angew. Chem. Int. Ed.* **2022**, 61, e202200337.

(37) X.-F. Luo, H.-X. Ni, A.-Q. Lv, X.-K. Yao, H.-L. Ma, Y.-X. Zheng, *Adv. Optical Mater*. **2022**, 10, 2200504.

(38) J. Liu, Y. Zhu, T. Tsuboi1, C. Deng, W. Lou, D. Wang, T. Liu, Q. Zhang, *Nat. Commun*. **2022**, 13 ,4876.

(39) X. C. Fan, K. Wang, Y. Z. Shi, Y. C. Cheng, Y. T. Lee, J. Yu, X. K. Chen, C. Adachi, X. H. Zhan, *Nat. Photonics* **2023**, *17*, 280–285.

(40) M. Wang, Z. Fu, R. Cheng, J. Du, T. Wu, Z. Bin, Di Wu, Y. Yang, J. Lan, *Chem. Commun.* **2023**, *59*, 5126.

(41) H.-X. Ni, W. Sun, X.-F. Luo, L. Yuan, X. Liang, X.-J. Liao, L. Zhou, Y.-X. Zheng, *The Innovation Materials* **2023**, 3, 100041.

(42) Y. Zou, M. Yu, J. Miao, T. Huang, S. Liao, X. Cao, C. Yang, *Chem. Sci.* **2023**, *14*, 3326.

(43) L. Liang, C. Qu, X. Fan, K. Ye, Y. Zhang, Z. Zhang, L. Duan, Y. Wang, *Angew. Chem. Int. Ed.* **2023**, 62, e202316710.

(44) X. Zhuang, J. Liang, X. Song, Q. Wang, H. Bi, B. Liang, Y. Wang, *Chem. Eng. J.* **2024**, 481, 148781.

(45) X.-F. Luo, L. Shen, J.-Y. Wanga, X. Xiao, *Chem. Commun.* **2024**, *60*, 574.

(46) Z. Zhang, Y. Zhang, P. Jiang, G. Lu, Z. Chen, H. Yu, Y. Qi, *Chem. Eur. J.* **2024**, 31, e202404484.

(47) T. Huang, Y. Xu, X. Lu, Y. Qu, J. Wei, Y. Wang, *Angew. Chem. Int. Ed.* **2024,** *63,* e202411268.

(48) J. Dong, Y. Xu, S. Wang, J. Miao, N. Li, Z. Huang, C. Yang, *Chem. Commun.* **2024**, *60*, 6789.

(49) S. Xiao, X. Cao, G. Chen, X. Yin, Z. Chen, J. Miao, C. Yang, *Angew. Chem. Int. Ed.* **2025**, *64,* e202418348.

(50) Z. Wu, P. Zou, J. Xu, X. Dong, B. Z. Tang, Z. Zhao, *Commun. Chem.* **2025**, *8*, 33.

(51) G. Meng, J. Zhou, Q. Wang, T. Huang, G. Zhang, L. Duan, D. Zhang, *Adv. Funct. Mater.* **2025**, *35*, 2422973.

(52) Z. Xue, Y. Hu, S. Xiao, J. Liu, J. Miao, C. Yang, *Angew. Chem. Int. Ed.* **2025,** *64,* e202500108.

(53) M Quan, Z.-L. Zhu, G. Chen, J. Miao, C. Yang, *Angew. Chem. Int. Ed*. **2025**, e202512162.

(54) Y. Zhang, G. Li, L. Wang, T. Huang, J. Wei, G. Meng, X. Wang, X. Zeng, D. Zhang, L. Duan, *Angew. Chem. Int. Ed*. **2022**, 61, e202202380.

(55) T. Fan, S. Zhu, X. Cao, X. Liang, M. Du, Y. Zhang, R. Liu, D. Zhang, L. Duan, *Angew. Chem. Int. Ed.* **2023,** *62,* e202313254.

(56) X. Wang, Y. Zhang, H. Dai, G. Li, M. Liu, G. Meng, X. Zeng, T. Huang, L. Wang, Q. Peng, D. Yang, D. Ma, D. Zhang, L. Duan, *Angew. Chem. Int. Ed.* **2022**, *61*, e202206916.

(57) Z.-G. Wu, Y. Xin, C. Lu, W. Huang, H. Xu, X. Liang, X. Cao, C. Li, D. Zhang, Y. Zhang, L. Duan, *Angew. Chem. Int. Ed.* **2023,** *62,* e202318742.

(58) T. Feng, X. Nie, D. Liu, L. Wu, C. Y. Liu, X. Mu, Z. Xin, B. Liu, H. Qi, J. Zhang, W. Li, S.-J. Su, Z. Ge, *Angew. Chem. Int. Ed.* **2024,** *63,* e202415113.

(59) X. Zeng, L. Wang, H. Dai, T. Huang, M. Du, D. Wang, D. Zhang, L. Duan, *Adv. Mater.* **2023**, *35*, 2211316.

(triphenylsilyl)phenyl)-1,3,5-triazine-2,4-diyl)bis(9*H*-carbazole) (SiTrzCz2, 5 nm); 9,10-bis(6-phenylpyridin-3-yl)anthracene/8-hydroxyquinolinolato-lithium (50%) (DPPyA/Liq, 30 nm); LiF (1 nm); Al (100 nm). The emitter molecule was not sublimated before the OLED fabrication in this study. The pressure during the vacuum evaporation was 5.0 × 10^{-4} Pa, and the film thickness was controlled using a calibrated quartz crystal microbalance during deposition. After the deposition of all layers, the OLED test modules were encapsulated with a capping glass in an evaporation chamber filled with nitrogen. The OLED characteristics of all fabricated devices were evaluated at room temperature (298 K) in an air atmosphere using a voltage–current–luminance measuring system, comprising a source meter (Keithley 2400) and spectral radiance meter (Topcon SR-3AR). The EQE was calculated using the EL spectrum, assuming the light-emitting surface to be a perfect diffusion surface and adding up all radiance elements from every angle and inputting them into the formula for obtaining EQE.

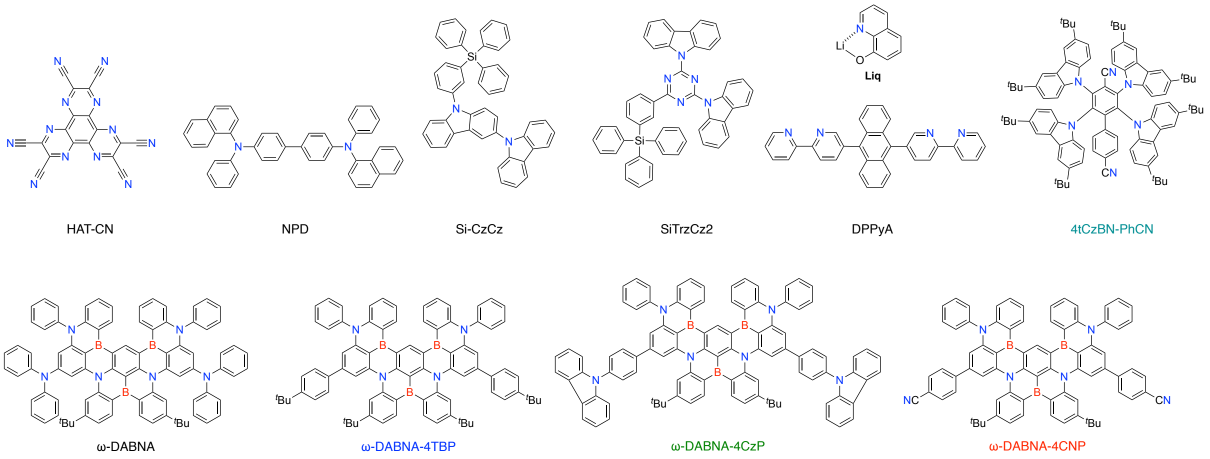

Figure S17. Molecular structures of HF devices employed in this study.

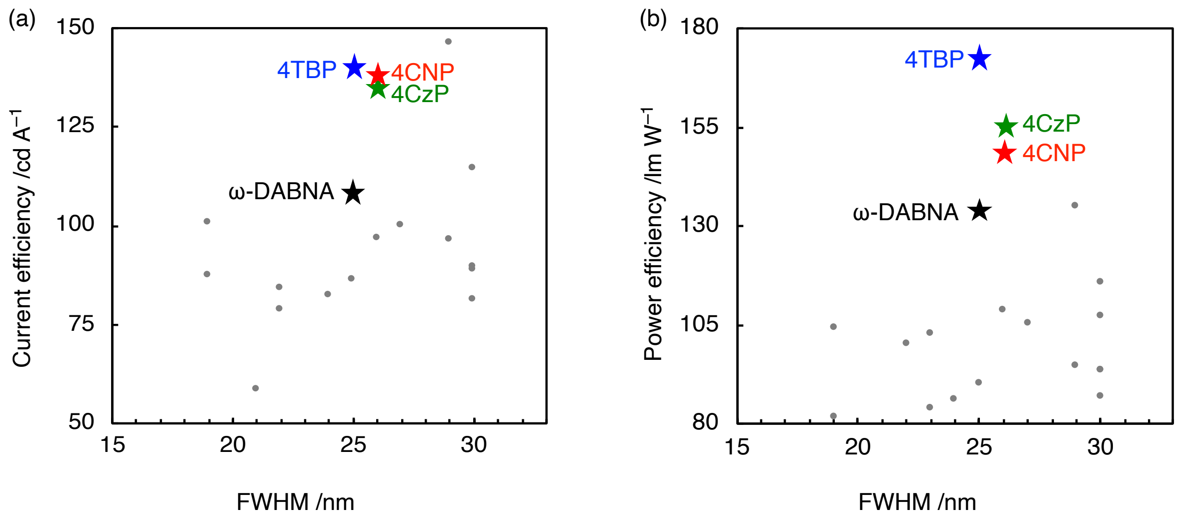

Figure S18. a,b) Comparison of maximum current efficiency and power efficiency values of reported green HF-OLEDs (FWHM of ≤ 30 nm) at 1000 cd m^{-2}. Highlighted in the plots are our compounds.

Table S5. Summary of HF-OLED Performance Employing Green MR-TADF Emitter.

Emitter	λ_{EL}	FWHM	V_{on}	EQE_{max}	CIE	EQE_{1000}	roll-off	CE_{max}	PE_{max}	PE_{1000}	Ref
	[nm][a]	[nm][b]	[eV][c]	[%][d]	[x,y][e]	[eV][f]	[%][g]	[cd A^{-1}][h]	[lm W^{-1}][i]	[lm W^{-1}][j]	
**ω-DABNA -4TBP**	523	25	2.5	36.1	0.19, 0.73	33.2	8.0	139.7	172.2	88.9	This work
**ω-DABNA -4CzP**	525	26	2.5	35.1	0.21, 0.71	34	3.1	135.0	155.3	90.4	This work
**ω-DABNA -4CNP**	533	26	2.5	33.0	0.25, 0.70	32.4	1.8	137.7	148.6	94.8	This work
**ω-DABNA**	510	25	2.5	34.8	0.15, 0.69	32.4	6.9	108.7	134.0	68.0	This work
**2PyBN**	501	26	2.4	38.0	0.12, 0.57	29.4	22.6	96.9	108.8	60.0	42
**DBNO**	504	27	3.0	37.1	0.14, 0.53	20.6	44.5	100.1	105.6	20.0	35
**IDID2BN**	534	29	3.20	36.6	0.31, 0.65	34.5	5.7	146.3	135.2	90.0	55
**pDBIC**	535	30	3.4	31.0	0.33, 0.64	20	35.5	-	87	50.0	56
**m-CzDAz-BNCz**	508	30	2.7	30.2	0.12, 0.65	22.8	24.5	81.4	93.6	50.0	47
**TPABO-DICz**	522	22	2.20	25.8	0.24, 0.74	25.6	0.8	84.1	100.5	78.5	57
**BN-ICz-1**	523	23	2.8	30.5	0.22, 0.74	17.2	43.6	-	84.2	37.7	54
**BN-ICz-2**	523	23	2.8	29.8	0.23, 0.73	26.1	12.4	-	102.9	54.6	54
**ω-DABNA**	512	24	3.0	28.0	0.15, 0.64	23.9	14.6	82.5	86.4	-	33
**ω-DABNA -M**	515	25	3.0	28.3	0.18, 0.65	25	11.7	86.4	90.5	-	33
**ω-DABNA -PH**	521	30	3.0	27.3	0.20, 0.66	24.6	9.9	89.6	93.8	-	33
**ω-DABNA -PH**	522	30	2.6	24.4	0.24, 0.70	18.5	24.2	89.0	107.5	-	33
**NT-2BN**	514	22	2.5	24.9	0.14, 0.70	14.7	41.0	78.8	-	-	58
**NT-3BN**	512	21	2.5	17.4	0.17, 0.70	7.2	58.6	58.7	-	-	58
**ββICZ**	508	19	2.4	30.9	0.22, 0.64	28.9	6.5	100.9	104.3	76.8	59
**ββCNICZ**	509	19	2.6	27.0	0.21, 0.65	25.5	5.6	87.3	81.9	66.8	59
**PhQA1**	534	29	3.2	23.8	0.33, 0.64	19.1	19.7	96.49	94.7	60.5	50
**PhQA3**	538	30	3.1	29.5	0.33, 0.62	24.8	15.9	114.5	116.0	79.1	50

[a]Maximum wavelength of EL spectrum. [b]Full width at half maximum. [c]Turn-on voltage at the luminescence of 1 cd m^{-2}. [d]Maximum External quantum efficiency. [e]CIE (x,y) coordinates.[f] External quantum efficiency at 1000 cd m^{-2}. [g]Efficiency roll-off at 1000 cd m^{-2} from EQE_{max}. [h]Maximum current efficiency [i]Maximum power efficiency. [j]Power efficiency at 1000 cd m^{-2}.

Table S6. The device operational lifetime of device E–H.

	Measured	Estimated
Device	LT_{80} at 5000 cd m^{-2}/ h	LT_{80} at 1000 cd m^{-2}/ h
E	196	3352
F	195	3543
G	25	457
H	12	224

Thermal Properties (TGA and DSC Analysis)

Thermogravimetric analysis (TGA) was performed to evaluate the thermal stability of **ω-DABNA-4TBP**, **ω-DABNA-4CzP**, and **ω-DABNA-4CNP** (**Figure S19**). The measurements were carried out under a nitrogen atmosphere with a heating rate of 10 °C min^{-1}. The T_{d5} values were determined to be 449 °C for **ω-DABNA-4TBP**, 545 °C **for ω-DABNA-4CzP**, and 474 °C for **ω-DABNA-4CNP**.

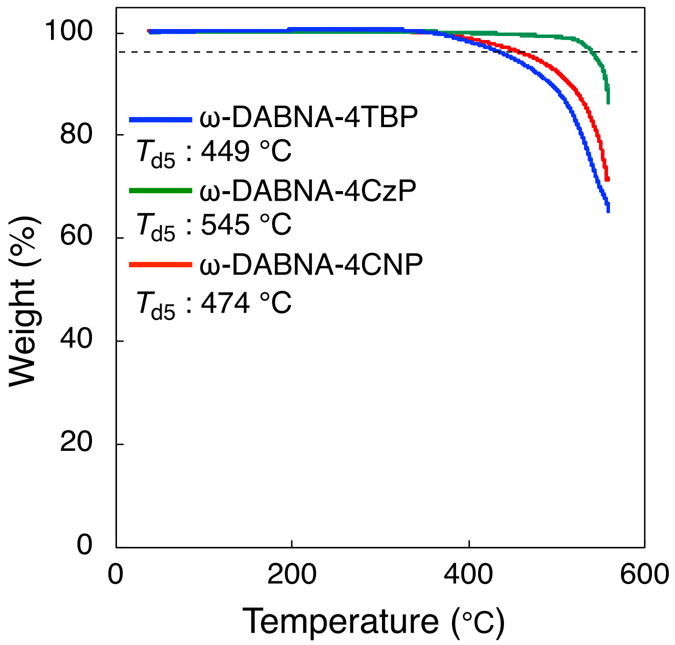

Figure S19. Thermogravimetric analysis (TGA) curves of **ω-DABNA-4TBP**, **ω-DABNA-4CzP**, and **ω-DABNA-4CNP** were measured under a nitrogen atmosphere. The decomposition temperature (T_{d5}) was defined as the temperature at which 5% weight loss occurs.

Differential scanning calorimetry (DSC) measurements were performed using heating–cooling cycles to assess the thermal transitions and stability of the materials (**Figure S20**). No discernible glass transition temperature (T_g) or other thermal transitions, such as melting or crystallization peaks, were observed within the measured temperature range. This indicates the absence of irreversible thermal events, demonstrating the high thermal stability of the materials.

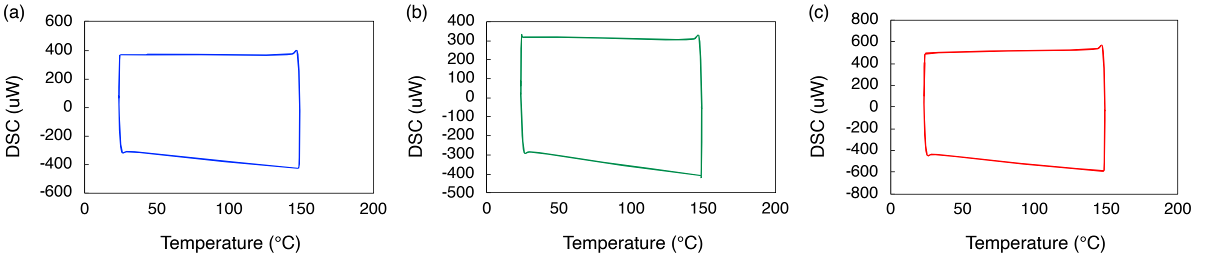

Figure S20. Differential scanning calorimetry (DSC) thermograms of the (a) **ω-DABNA-4TBP**, (b) **ω-DABNA-4CzP**, and (c) **ω-DABNA-4CNP** recorded during the second heating cycle under a nitrogen atmosphere.

Thermal gravimetric analysis and differential scanning calorimetry measurements indicate that all ω-DABNA derivatives exhibit high thermal stability without detectable glass transition or phase transitions under device-relevant conditions (**Figures S19** and **S20**), supporting their suitability for vacuum deposition and OLED fabrication.

UV–vis absorption spectra and extinction coefficients.

The molar extinction coefficient (ε) versus wavelength plots of ω-DABNA-derivatives were measured in dilute 3-methylpentane solution (1 × 10 M^{-5}) at room temperature and are shown in **Figure S21**. All three compounds exhibit intense and well-defined absorption bands in the visible region with large molar extinction coefficients, indicating strongly allowed electronic transitions. The experimentally obtained absorption intensities are consistent with the relatively large oscillator strengths for the S_1 states calculated by TD-DFT. The sharp absorption features and the small Stokes shifts previously observed for all derivatives are characteristic of short-range charge-transfer (SR-CT) transitions commonly found in boron/nitrogen-based multiple-resonance (MR) emitters. Notably, the absorption spectral profiles are very similar across the three compounds, suggesting that the peripheral substituents (^{t}Bu, Cz, and CN) do not significantly perturb the intrinsic MR electronic structure of the ω-DABNA core in the ground state.

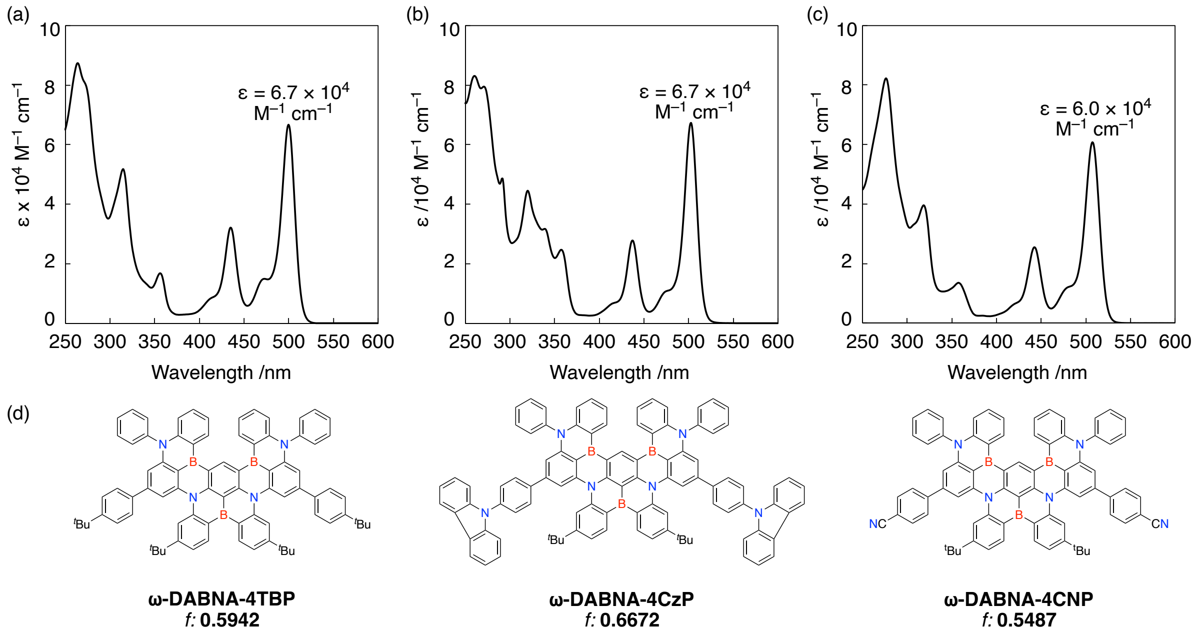

Figure S21. Molar extinction coefficient (ε, × 10^4 M^{-1} cm^{-1}) versus wavelength plots of a) **ω-DABNA-4TBP**, b) **ω-DABNA-4CzP**, and c) **ω-DABNA-4CNP** measured in 3-methylpentane solution (1 × 10^{-5} M) at room temperature. (d) Calculated oscillator strengths for the S_1 states of the three compounds obtained from TD-DFT calculations.

Temperature-Dependent Time-Resolved Photoluminescence (TRPL)

TRPL measurements were carried out for ω-DABNA derivatives at six temperatures over a wide temperature range to investigate the evolution of the excited-state dynamics. The decay profiles were recorded at the emission maxima and analyzed as a function of temperature by separating the prompt and delayed components.

With increasing temperature, the delayed emission component becomes more pronounced, while the prompt fluorescence component remains nearly unchanged. This behavior is consistent with thermally activated RISC, which is a characteristic feature of TADF-active MR emitters. The similar temperature-dependent trends observed for the three derivatives indicate that the peripheral substituents do not significantly alter the intrinsic excited-state dynamics of the ω-DABNA core.

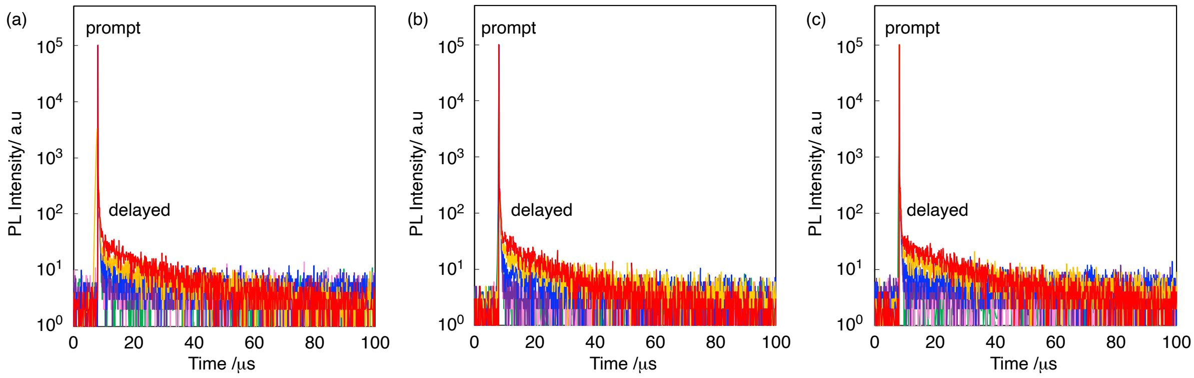

Figure S22. Time-resolved photoluminescence (TRPL) decay curves of 1 wt.% PMMA-doped films of (a) **ω-DABNA-4TBP**, (b) **ω-DABNA-4CzP**, and (c) **ω-DABNA-4CNP** measured at different temperatures. The transient decay spectra were recorded at 77 K (green), 100 K (magenta), 150 K (purple), 200 K (blue), 250 K (orange), and 300 K (red).

Table S7. Summary of the percentage of prompt and delayed components obtained from temperature-dependent TRPL decay analysis.

Temperature (K)	**ω–DABNA-4TBP**		**ω–DABNA-4CzP**		**ω–DABNA-4CNP**	
	Prompt (%)	Delayed (%)	Prompt (%)	Delayed (%)	Prompt (%)	Delayed (%)
77	100	0	100	0	100	0
100	100	0	100	0	100	0
150	100	0	100	0	100	0
200	98.1	1.9	97.6	2.4	96.9	3.1
250	97.3	2.7	97.8	3.2	96.3	3.7

300	95.7	4.3	95.7	4.3	95.0	5.0

Bond Dissociation Energy (E_{BDE})

Table S8. Calculated bond dissociation energy (E_{BDE}) for the C–R bond cleavage in **ω-DABNA-4TBP**, **ω-DABNA-4CzP**, and **ω-DABNA-4CNP**, evaluated using electronic energies of the optimized structures at the B3LYP/6-31G(d) level of theory.

States

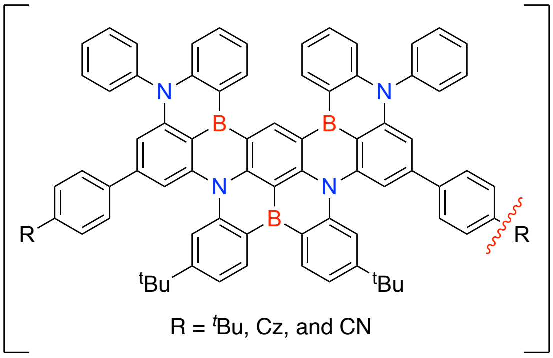

Bond dissociation energy (E_{BDE}, eV)				
States	Dissociation products	**ω-DABNA-4TBP**	**ω-DABNA-4CzP**	**ω-DABNA-4CNP**
Radical anion	Radical (core)[a] + Anion (R)[b]	3.84	3.79	6.25
Radical anion	Anion (core)[a] + Radical (R)[b]	5.84	2.79	4.22
Neutral	Radical (core)[a] + Radical (R)[b]	3.94	3.73	5.99

[a]Fragment associated with the ω-DABNA core after C–R bond cleavage. [b]Fragment originating from the cleaved terminal substituent (R).

To evaluate the intrinsic chemical robustness of the terminal emitters, bond dissociation energy (E_{BDE}) was calculated for **ω-DABNA-4TBP**, **ω-DABNA-4CzP**, and **ω-DABNA-4CNP**. The calculations were performed for the relevant C–R bond. The calculations focused on the relevant C–R bond linking the ω-DABNA core phenyl unit and the terminal substituent (R = ^{t}Bu, Cz, and CN). The calculated E_{BDE} values are summarized in **Table S8**. A clear dependence of the E_{BDE} on the terminal substituent is observed. Notably, **ω-DABNA-4CzP** exhibits a lower E_{BDE} than **ω-DABNA-4TBP** and **ω-DABNA-4CNP**, indicating a relatively weaker carbazole–phenyl linkage. Although E_{BDE} calculations do not directly describe the full degradation pathways in operating devices, they are widely used as indicators of relative bond robustness. In this context, the reduced E_{BDE} of **ω-DABNA-4CzP** is consistent with its comparatively lower operational stability observed in TADF devices.

Cartesian coordinates

b (S_0, C_2 symmetry, Gaussian)
E(B3LYP/6–31G(d)) = −3558.85777970 hartree

Center Number	Atomic Number	Atomic Type	Coordinates (Angstroms) X	Y	Z
1	5	0	0.000000	0.000000	-2.072880
2	7	0	0.589289	-2.368279	-0.527927
3	7	0	-0.589289	2.368279	-0.527927
4	6	0	-0.298669	1.186360	0.171724
5	6	0	0.000000	0.000000	-0.554994
6	6	0	0.298669	-1.186360	0.171724
7	6	0	0.313079	-1.177381	1.577181
8	6	0	0.000000	0.000000	2.247296
9	6	0	-0.313079	1.177381	1.577181
10	1	0	0.000000	0.000000	3.334667
11	6	0	-0.450944	2.507944	-1.923440
12	6	0	-0.641208	3.784243	-2.502796
13	6	0	-0.114811	1.391812	-2.734700
14	6	0	-0.446449	4.013178	-3.860757
15	1	0	-0.931912	4.606334	-1.866427
16	6	0	0.118468	1.668934	-4.101094
17	6	0	-0.033797	2.925752	-4.657291
18	1	0	0.166904	3.066065	-5.715445
19	6	0	0.450944	-2.507944	-1.923440
20	6	0	0.114811	-1.391812	-2.734700
21	6	0	0.641208	-3.784243	-2.502796
22	6	0	-0.118468	-1.668934	-4.101094
23	6	0	0.446449	-4.013178	-3.860757
24	1	0	0.931912	-4.606334	-1.866427
25	6	0	0.033797	-2.925752	-4.657291
26	1	0	-0.166904	-3.066065	-5.715445
27	6	0	-0.976226	3.520928	0.245027
28	6	0	0.000000	4.407173	0.696429
29	6	0	-2.329945	3.735032	0.514826
30	6	0	-0.362394	5.536649	1.451657
31	6	0	-2.675015	4.860826	1.260331
32	1	0	-3.081281	3.043731	0.152784
33	6	0	-1.722890	5.753474	1.739476
34	6	0	0.976226	-3.520928	0.245027
35	6	0	0.000000	-4.407173	0.696429
36	6	0	2.329945	-3.735032	0.514826
37	6	0	0.362394	-5.536649	1.451657
38	6	0	2.675015	-4.860826	1.260331
39	1	0	3.081281	-3.043731	0.152784
40	6	0	1.722890	-5.753474	1.739476
41	1	0	0.459454	0.863476	-4.741575
42	1	0	-0.459454	-0.863476	-4.741575
43	6	0	-0.646653	5.399243	-4.499095
44	6	0	-1.730926	5.300835	-5.598914
45	6	0	-1.094249	6.464696	-3.480417
46	6	0	0.684244	5.869680	-5.133219
47	1	0	-1.454318	4.585587	-6.380207
48	1	0	-2.689350	4.979141	-5.175794
49	1	0	-1.879840	6.277331	-6.076250
50	1	0	-0.349012	6.609600	-2.689467
51	1	0	-1.227953	7.426584	-3.988402

52	1	0	-2.048596	6.203603	-3.009348
53	1	0	0.555982	6.852930	-5.602619
54	1	0	1.470775	5.954661	-4.374663
55	1	0	1.037225	5.176263	-5.903277
56	6	0	0.646653	-5.399243	-4.499095
57	6	0	1.730926	-5.300835	-5.598914
58	6	0	1.094249	-6.464696	-3.480417
59	6	0	-0.684244	-5.869680	-5.133219
60	1	0	1.454318	-4.585587	-6.380207
61	1	0	2.689350	-4.979141	-5.175794
62	1	0	1.879840	-6.277331	-6.076250
63	1	0	0.349012	-6.609600	-2.689467
64	1	0	1.227953	-7.426584	-3.988402
65	1	0	2.048596	-6.203603	-3.009348
66	1	0	-0.555982	-6.852930	-5.602619
67	1	0	-1.470775	-5.954661	-4.374663
68	1	0	-1.037225	-5.176263	-5.903277
69	7	0	0.616918	6.439281	1.916185
70	7	0	-0.616918	-6.439281	1.916185
71	6	0	1.916521	5.975072	2.279033
72	6	0	3.057621	6.662633	1.840001
73	6	0	2.069911	4.838104	3.086527
74	6	0	4.327078	6.220693	2.208602
75	1	0	2.942294	7.540875	1.212628
76	6	0	3.343850	4.391846	3.434000
77	6	0	4.478500	5.081631	3.001976
78	1	0	5.201999	6.763295	1.860647
79	1	0	3.447035	3.507328	4.056669
80	6	0	-1.916521	-5.975072	2.279033
81	6	0	-3.057621	-6.662633	1.840001
82	6	0	-2.069911	-4.838104	3.086527
83	6	0	-4.327078	-6.220693	2.208602
84	1	0	-2.942294	-7.540875	1.212628
85	6	0	-3.343850	-4.391846	3.434000
86	6	0	-4.478500	-5.081631	3.001976
87	1	0	-5.201999	-6.763295	1.860647
88	1	0	-3.447035	-3.507328	4.056669
89	6	0	0.326167	7.831757	2.023596
90	6	0	0.695429	8.541443	3.175922
91	6	0	-0.317078	8.510721	0.977474
92	6	0	0.429468	9.906147	3.274007
93	1	0	1.191226	8.018362	3.987595
94	6	0	-0.595919	9.871595	1.092123
95	6	0	-0.221436	10.577865	2.237156
96	1	0	0.721698	10.442723	4.172725
97	1	0	-1.095692	10.383648	0.274221
98	6	0	-0.326167	-7.831757	2.023596
99	6	0	-0.695429	-8.541443	3.175922
100	6	0	0.317078	-8.510721	0.977474
101	6	0	-0.429468	-9.906147	3.274007
102	1	0	-1.191226	-8.018362	3.987595
103	6	0	0.595919	-9.871595	1.092123
104	6	0	0.221436	-10.577865	2.237156
105	1	0	-0.721698	-10.442723	4.172725
106	1	0	1.095692	-10.383648	0.274221
107	1	0	-0.433852	11.639868	2.319987
108	1	0	5.469535	4.734818	3.280065
109	1	0	-5.469535	-4.734818	3.280065
110	1	0	0.433852	-11.639868	2.319987
111	1	0	-0.595496	7.968676	0.078897

112	1	0	-2.028154	6.607725	2.330957
113	1	0	2.028154	-6.607725	2.330957
114	1	0	0.595496	-7.968676	0.078897
115	17	0	-4.370952	5.152724	1.630029
116	17	0	4.370952	-5.152724	1.630029
117	1	0	1.189806	4.308138	3.437235
118	1	0	-1.189806	-4.308138	3.437235
119	1	0	-0.551533	2.068244	2.142390
120	1	0	0.551533	-2.068244	2.142390
121	1	0	-1.039340	-4.219211	0.454271
122	1	0	1.039340	4.219211	0.454271

ω-DABNA-Cl (S_0, C_2 symmetry, Gaussian)
E(B3LYP/6–31G(d)) = −2714.51810013 hartree

Center Number	Atomic Number	Atomic Type	Coordinates (Angstroms) X	Y	Z
1	5	0	0.000000	0.000000	-2.775119
2	7	0	-0.284837	-2.413386	-1.263264
3	7	0	0.284837	2.413386	-1.263264
4	6	0	0.158742	1.211265	-0.537624
5	6	0	0.000000	0.000000	-1.257746
6	6	0	-0.158742	-1.211265	-0.537624
7	6	0	-0.190478	-1.233029	0.885005
8	6	0	0.000000	0.000000	1.527115
9	6	0	0.190478	1.233029	0.885005
10	1	0	0.000000	0.000000	2.610759
11	6	0	0.776711	2.400357	-2.605622
12	6	0	1.515217	3.503516	-3.078827
13	6	0	0.593730	1.260976	-3.421808
14	6	0	2.035239	3.540739	-4.371414
15	1	0	1.697764	4.331141	-2.408464
16	6	0	1.130469	1.316597	-4.726757
17	6	0	1.815079	2.421589	-5.201440
18	1	0	2.208400	2.408287	-6.213674
19	6	0	-0.776711	-2.400357	-2.605622
20	6	0	-0.593730	-1.260976	-3.421808
21	6	0	-1.515217	-3.503516	-3.078827
22	6	0	-1.130469	-1.316597	-4.726757
23	6	0	-2.035239	-3.540739	-4.371414
24	1	0	-1.697764	-4.331141	-2.408464
25	6	0	-1.815079	-2.421589	-5.201440
26	1	0	-2.208400	-2.408287	-6.213674
27	6	0	-0.099312	3.642298	-0.660258
28	6	0	0.000000	3.784876	0.745011
29	6	0	-0.613477	4.680791	-1.447891
30	6	0	-0.329418	5.030851	1.340691
31	6	0	-0.948295	5.867834	-0.810474
32	1	0	-0.762971	4.572870	-2.512739
33	6	0	-0.810461	6.092133	0.548710
34	6	0	0.099312	-3.642298	-0.660258
35	6	0	0.000000	-3.784876	0.745011
36	6	0	0.613477	-4.680791	-1.447891
37	6	0	0.329418	-5.030851	1.340691
38	6	0	0.948295	-5.867834	-0.810474
39	1	0	0.762971	-4.572870	-2.512739
40	6	0	0.810461	-6.092133	0.548710
41	1	0	1.029973	0.444004	-5.365910

42	1	0	-1.029973	-0.444004	-5.365910
43	6	0	2.846659	4.738233	-4.897847
44	6	0	2.134658	5.325374	-6.140662
45	6	0	2.991721	5.861938	-3.853855
46	6	0	4.264663	4.263077	-5.296278
47	1	0	2.034018	4.585743	-6.941835
48	1	0	1.130258	5.681919	-5.885314
49	1	0	2.704967	6.173446	-6.538970
50	1	0	3.524456	5.521653	-2.958438
51	1	0	3.567162	6.689654	-4.283631
52	1	0	2.019814	6.262222	-3.543788
53	1	0	4.852237	5.104524	-5.683488
54	1	0	4.795374	3.846768	-4.432425
55	1	0	4.234508	3.492162	-6.073253
56	6	0	-2.846659	-4.738233	-4.897847
57	6	0	-2.134658	-5.325374	-6.140662
58	6	0	-2.991721	-5.861938	-3.853855
59	6	0	-4.264663	-4.263077	-5.296278
60	1	0	-2.034018	-4.585743	-6.941835
61	1	0	-1.130258	-5.681919	-5.885314
62	1	0	-2.704967	-6.173446	-6.538970
63	1	0	-3.524456	-5.521653	-2.958438
64	1	0	-3.567162	-6.689654	-4.283631
65	1	0	-2.019814	-6.262222	-3.543788
66	1	0	-4.852237	-5.104524	-5.683488
67	1	0	-4.795374	-3.846768	-4.432425
68	1	0	-4.234508	-3.492162	-6.073253
69	7	0	-0.188827	5.192275	2.723924
70	7	0	0.188827	-5.192275	2.723924
71	6	0	0.355499	4.205145	3.572850
72	6	0	0.608700	4.524543	4.925351
73	6	0	0.647876	2.898886	3.085095
74	6	0	1.176250	3.584873	5.773569
75	1	0	0.375382	5.509675	5.308821
76	6	0	1.249229	1.988968	3.984134
77	6	0	1.516737	2.309127	5.306435
78	1	0	1.369027	3.857418	6.808175
79	1	0	1.531889	1.009206	3.612304
80	6	0	-0.355499	-4.205145	3.572850
81	6	0	-0.608700	-4.524543	4.925351
82	6	0	-0.647876	-2.898886	3.085095
83	6	0	-1.176250	-3.584873	5.773569
84	1	0	-0.375382	-5.509675	5.308821
85	6	0	-1.249229	-1.988968	3.984134
86	6	0	-1.516737	-2.309127	5.306435
87	1	0	-1.369027	-3.857418	6.808175
88	1	0	-1.531889	-1.009206	3.612304
89	5	0	0.333202	2.584534	1.611363
90	5	0	-0.333202	-2.584534	1.611363
91	6	0	-0.588287	6.452522	3.301118
92	6	0	-1.886550	6.607060	3.794460
93	6	0	0.318576	7.514855	3.360233
94	6	0	-2.276852	7.827199	4.349207
95	1	0	-2.578696	5.772208	3.739252
96	6	0	-0.076426	8.733443	3.915168
97	6	0	-1.373311	8.890716	4.409982
98	1	0	-3.286725	7.946374	4.731430
99	1	0	0.628829	9.558539	3.959968
100	6	0	0.588287	-6.452522	3.301118
101	6	0	1.886550	-6.607060	3.794460

102	6	0	-0.318576	-7.514855	3.360233
103	6	0	2.276852	-7.827199	4.349207
104	1	0	2.578696	-5.772208	3.739252
105	6	0	0.076426	-8.733443	3.915168
106	6	0	1.373311	-8.890716	4.409982
107	1	0	3.286725	-7.946374	4.731430
108	1	0	-0.628829	-9.558539	3.959968
109	1	0	-1.679238	9.840011	4.840686
110	1	0	1.983736	1.585369	5.968159
111	1	0	-1.983736	-1.585369	5.968159
112	1	0	1.679238	-9.840011	4.840686
113	1	0	1.323569	7.378487	2.972022
114	1	0	-1.075101	7.051582	0.968330
115	1	0	1.075101	-7.051582	0.968330
116	1	0	-1.323569	-7.378487	2.972022
117	17	0	-1.609475	7.206464	-1.823641
118	17	0	1.609475	-7.206464	-1.823641

ω-DABNA (S_0, C_2 symmetry, Gaussian)
E(B3LYP/6–31G(d)) = −3720.78087742 hartree

Center Number	Atomic Number	Atomic Type	Coordinates (Angstroms) X	Y	Z
1	5	0	0.000000	0.000000	-2.200737
2	7	0	-0.493814	-2.380533	-0.690702
3	7	0	0.493814	2.380533	-0.690702
4	6	0	0.264381	1.193754	0.034942
5	6	0	0.000000	0.000000	-0.684757
6	6	0	-0.264381	-1.193754	0.034942
7	6	0	-0.298965	-1.211617	1.457051
8	6	0	0.000000	0.000000	2.098079
9	6	0	0.298965	1.211617	1.457051
10	1	0	0.000000	0.000000	3.181891
11	6	0	0.981618	2.323678	-2.030867
12	6	0	1.811248	3.359221	-2.506878
13	6	0	0.702173	1.204270	-2.847684
14	6	0	2.327600	3.353722	-3.800392
15	1	0	2.062356	4.167972	-1.835905
16	6	0	1.238902	1.214617	-4.153491
17	6	0	2.011794	2.258076	-4.630744
18	1	0	2.397534	2.213267	-5.645189
19	6	0	-0.981618	-2.323678	-2.030867
20	6	0	-0.702173	-1.204270	-2.847684
21	6	0	-1.811248	-3.359221	-2.506878
22	6	0	-1.238902	-1.214617	-4.153491
23	6	0	-2.327600	-3.353722	-3.800392
24	1	0	-2.062356	-4.167972	-1.835905
25	6	0	-2.011794	-2.258076	-4.630744
26	1	0	-2.397534	-2.213267	-5.645189
27	6	0	0.213995	3.641276	-0.085745
28	6	0	0.341712	3.768805	1.319015
29	6	0	-0.202900	4.719225	-0.866413
30	6	0	0.128205	5.043413	1.905796
31	6	0	-0.450946	5.961052	-0.259655
32	1	0	-0.343471	4.614480	-1.933163
33	6	0	-0.276608	6.132114	1.117881
34	1	0	-0.475839	7.099298	1.556838
35	6	0	-0.213995	-3.641276	-0.085745

36	6	0	-0.341712	-3.768805	1.319015
37	6	0	0.202900	-4.719225	-0.866413
38	6	0	-0.128205	-5.043413	1.905796
39	6	0	0.450946	-5.961052	-0.259655
40	1	0	0.343471	-4.614480	-1.933163
41	6	0	0.276608	-6.132114	1.117881
42	1	0	0.475839	-7.099298	1.556838
43	1	0	1.062991	0.353670	-4.792313
44	1	0	-1.062991	-0.353670	-4.792313
45	7	0	-0.882500	7.053782	-1.055642
46	7	0	0.882500	-7.053782	-1.055642
47	6	0	-0.405710	8.368168	-0.793880
48	6	0	0.951207	8.594780	-0.511569
49	6	0	-1.285030	9.462046	-0.828174
50	6	0	1.411297	9.886290	-0.262220
51	1	0	1.638125	7.754696	-0.489597
52	6	0	-0.812535	10.752186	-0.594522
53	1	0	-2.335939	9.293103	-1.040473
54	6	0	0.535719	10.973511	-0.306219
55	1	0	2.465371	10.043510	-0.048060
56	1	0	-1.507750	11.587152	-0.625618
57	1	0	0.899561	11.979872	-0.119149
58	6	0	0.405710	-8.368168	-0.793880
59	6	0	-0.951207	-8.594780	-0.511569
60	6	0	1.285030	-9.462046	-0.828174
61	6	0	-1.411297	-9.886290	-0.262220
62	1	0	-1.638125	-7.754696	-0.489597
63	6	0	0.812535	-10.752186	-0.594522
64	1	0	2.335939	-9.293103	-1.040473
65	6	0	-0.535719	-10.973511	-0.306219
66	1	0	-2.465371	-10.043510	-0.048060
67	1	0	1.507750	-11.587152	-0.625618
68	1	0	-0.899561	-11.979872	-0.119149
69	6	0	-1.780742	6.840459	-2.139115
70	6	0	-2.898001	6.004577	-1.984887
71	6	0	-1.563591	7.467004	-3.376636
72	6	0	-3.770130	5.794134	-3.051267
73	1	0	-3.074219	5.521854	-1.028937
74	6	0	-2.451803	7.265418	-4.431648
75	1	0	-0.699194	8.111188	-3.503409
76	6	0	-3.556733	6.425383	-4.278566
77	1	0	-4.628736	5.141807	-2.915481
78	1	0	-2.268934	7.758195	-5.383048
79	1	0	-4.242452	6.263927	-5.105439
80	6	0	1.780742	-6.840459	-2.139115
81	6	0	2.898001	-6.004577	-1.984887
82	6	0	1.563591	-7.467004	-3.376636
83	6	0	3.770130	-5.794134	-3.051267
84	1	0	3.074219	-5.521854	-1.028937
85	6	0	2.451803	-7.265418	-4.431648
86	1	0	0.699194	-8.111188	-3.503409
87	6	0	3.556733	-6.425383	-4.278566
88	1	0	4.628736	-5.141807	-2.915481
89	1	0	2.268934	-7.758195	-5.383048
90	1	0	4.242452	-6.263927	-5.105439
91	6	0	3.226112	4.484742	-4.332391
92	6	0	2.531499	5.149837	-5.545304
93	6	0	3.495792	5.574924	-3.277811
94	6	0	4.587890	3.900539	-4.777602
95	1	0	2.346503	4.432802	-6.351938

96	1	0	1.567224	5.582595	-5.255111
97	1	0	3.158633	5.954069	-5.949715
98	1	0	4.012496	5.173037	-2.398751
99	1	0	4.135357	6.353287	-3.709353
100	1	0	2.570847	6.057078	-2.941422
101	1	0	5.233628	4.696392	-5.168747
102	1	0	5.104556	3.426580	-3.935250
103	1	0	4.473224	3.148317	-5.564698
104	6	0	-3.226112	-4.484742	-4.332391
105	6	0	-2.531499	-5.149837	-5.545304
106	6	0	-3.495792	-5.574924	-3.277811
107	6	0	-4.587890	-3.900539	-4.777602
108	1	0	-2.346503	-4.432802	-6.351938
109	1	0	-1.567224	-5.582595	-5.255111
110	1	0	-3.158633	-5.954069	-5.949715
111	1	0	-4.012496	-5.173037	-2.398751
112	1	0	-4.135357	-6.353287	-3.709353
113	1	0	-2.570847	-6.057078	-2.941422
114	1	0	-5.233628	-4.696392	-5.168747
115	1	0	-5.104556	-3.426580	-3.935250
116	1	0	-4.473224	-3.148317	-5.564698
117	7	0	0.287449	5.192576	3.291867
118	7	0	-0.287449	-5.192576	3.291867
119	6	0	0.739135	4.161168	4.141931
120	6	0	1.027515	4.458681	5.492358
121	6	0	0.907515	2.831889	3.657850
122	6	0	1.506898	3.472643	6.342307
123	1	0	0.891020	5.462867	5.873322
124	6	0	1.422310	1.872769	4.558140
125	6	0	1.722781	2.169416	5.879048
126	1	0	1.728788	3.729702	7.375207
127	1	0	1.609215	0.869313	4.188787
128	1	0	2.120484	1.406095	6.541797
129	6	0	-0.739135	-4.161168	4.141931
130	6	0	-1.027515	-4.458681	5.492358
131	6	0	-0.907515	-2.831889	3.657850
132	6	0	-1.506898	-3.472643	6.342307
133	1	0	-0.891020	-5.462867	5.873322
134	6	0	-1.422310	-1.872769	4.558140
135	6	0	-1.722781	-2.169416	5.879048
136	1	0	-1.728788	-3.729702	7.375207
137	1	0	-1.609215	-0.869313	4.188787
138	1	0	-2.120484	-1.406095	6.541797
139	5	0	0.564712	2.546399	2.182716
140	5	0	-0.564712	-2.546399	2.182716
141	6	0	0.000000	6.480558	3.871588
142	6	0	-1.270180	6.736534	4.394708
143	6	0	0.986537	7.469844	3.909224
144	6	0	-1.551723	7.982236	4.957385
145	1	0	-2.025643	5.957382	4.357356
146	6	0	0.700469	8.715001	4.470959
147	1	0	1.968537	7.255773	3.498142
148	6	0	-0.567706	8.972382	4.995898
149	1	0	-2.540072	8.178848	5.363557
150	1	0	1.468290	9.483135	4.497502
151	1	0	-0.788859	9.942383	5.432539
152	6	0	0.000000	-6.480558	3.871588
153	6	0	1.270180	-6.736534	4.394708
154	6	0	-0.986537	-7.469844	3.909224
155	6	0	1.551723	-7.982236	4.957385

156	1	0	2.025643	-5.957382	4.357356
157	6	0	-0.700469	-8.715001	4.470959
158	1	0	-1.968537	-7.255773	3.498142
159	6	0	0.567706	-8.972382	4.995898
160	1	0	2.540072	-8.178848	5.363557
161	1	0	-1.468290	-9.483135	4.497502
162	1	0	0.788859	-9.942383	5.432539

ω-DABNA (S_1, C_2 symmetry, Gaussian)
E(B3LYP/6–31G(d)) = −3720.77935956 hartree

Center Number	Atomic Number	Atomic Type	Coordinates (Angstroms) X	Y	Z
1	5	0	0.000000	0.000000	-2.174331
2	7	0	-0.497140	-2.371745	-0.656383
3	7	0	0.497140	2.371745	-0.656383
4	6	0	0.259561	1.188246	0.074857
5	6	0	0.000000	0.000000	-0.654509
6	6	0	-0.259561	-1.188246	0.074857
7	6	0	-0.286344	-1.213993	1.504113
8	6	0	0.000000	0.000000	2.158700
9	6	0	0.286344	1.213993	1.504113
10	1	0	0.000000	0.000000	3.240194
11	6	0	1.023215	2.304974	-1.989010
12	6	0	1.897642	3.315838	-2.426584
13	6	0	0.714246	1.197172	-2.816617
14	6	0	2.418619	3.328420	-3.721940
15	1	0	2.175997	4.094744	-1.730214
16	6	0	1.250774	1.230915	-4.123249
17	6	0	2.056302	2.264751	-4.573733
18	1	0	2.436589	2.231076	-5.591290
19	6	0	-1.023215	-2.304974	-1.989010
20	6	0	-0.714246	-1.197172	-2.816617
21	6	0	-1.897642	-3.315838	-2.426584
22	6	0	-1.250774	-1.230915	-4.123249
23	6	0	-2.418619	-3.328420	-3.721940
24	1	0	-2.175997	-4.094744	-1.730214
25	6	0	-2.056302	-2.264751	-4.573733
26	1	0	-2.436589	-2.231076	-5.591290
27	6	0	0.236880	3.631335	-0.071502
28	6	0	0.328049	3.769183	1.335825
29	6	0	-0.146406	4.714861	-0.875614
30	6	0	0.121117	5.059090	1.898087
31	6	0	-0.403308	5.963251	-0.289851
32	1	0	-0.262430	4.597405	-1.944171
33	6	0	-0.256544	6.145258	1.092557
34	1	0	-0.455235	7.118772	1.517494
35	6	0	-0.236880	-3.631335	-0.071502
36	6	0	-0.328049	-3.769183	1.335825
37	6	0	0.146406	-4.714861	-0.875614
38	6	0	-0.121117	-5.059090	1.898087
39	6	0	0.403308	-5.963251	-0.289851
40	1	0	0.262430	-4.597405	-1.944171
41	6	0	0.256544	-6.145258	1.092557
42	1	0	0.455235	-7.118772	1.517494
43	1	0	1.051203	0.394098	-4.786859
44	1	0	-1.051203	-0.394098	-4.786859
45	7	0	-0.808537	7.047756	-1.111034

46	7	0	0.808537	-7.047756	-1.111034
47	6	0	-0.284402	8.349993	-0.887817
48	6	0	1.076776	8.531183	-0.591423
49	6	0	-1.119355	9.476517	-0.965906
50	6	0	1.584330	9.810422	-0.374228
51	1	0	1.729792	7.666055	-0.535630
52	6	0	-0.598736	10.753055	-0.762885
53	1	0	-2.173562	9.343488	-1.186824
54	6	0	0.753525	10.929790	-0.462145
55	1	0	2.641078	9.932154	-0.150400
56	1	0	-1.259885	11.613343	-0.827909
57	1	0	1.154693	11.926094	-0.299363
58	6	0	0.284402	-8.349993	-0.887817
59	6	0	-1.076776	-8.531183	-0.591423
60	6	0	1.119355	-9.476517	-0.965906
61	6	0	-1.584330	-9.810422	-0.374228
62	1	0	-1.729792	-7.666055	-0.535630
63	6	0	0.598736	-10.753055	-0.762885
64	1	0	2.173562	-9.343488	-1.186824
65	6	0	-0.753525	-10.929790	-0.462145
66	1	0	-2.641078	-9.932154	-0.150400
67	1	0	1.259885	-11.613343	-0.827909
68	1	0	-1.154693	-11.926094	-0.299363
69	6	0	-1.730906	6.828404	-2.170552
70	6	0	-2.851889	6.004161	-1.978629
71	6	0	-1.532023	7.428715	-3.424971
72	6	0	-3.746134	5.780747	-3.023529
73	1	0	-3.014601	5.542947	-1.009810
74	6	0	-2.441806	7.212961	-4.457952
75	1	0	-0.663753	8.060628	-3.582352
76	6	0	-3.550893	6.385669	-4.267075
77	1	0	-4.607022	5.138360	-2.858515
78	1	0	-2.272227	7.683318	-5.423020
79	1	0	-4.252932	6.212989	-5.077747
80	6	0	1.730906	-6.828404	-2.170552
81	6	0	2.851889	-6.004161	-1.978629
82	6	0	1.532023	-7.428715	-3.424971
83	6	0	3.746134	-5.780747	-3.023529
84	1	0	3.014601	-5.542947	-1.009810
85	6	0	2.441806	-7.212961	-4.457952
86	1	0	0.663753	-8.060628	-3.582352
87	6	0	3.550893	-6.385669	-4.267075
88	1	0	4.607022	-5.138360	-2.858515
89	1	0	2.272227	-7.683318	-5.423020
90	1	0	4.252932	-6.212989	-5.077747
91	6	0	3.358867	4.436628	-4.226489
92	6	0	2.695636	5.157678	-5.424986
93	6	0	3.665820	5.489982	-3.144957
94	6	0	4.700005	3.814239	-4.683097
95	1	0	2.484880	4.467014	-6.248244
96	1	0	1.747607	5.620080	-5.126810
97	1	0	3.354202	5.946391	-5.810243
98	1	0	4.162729	5.046908	-2.274224
99	1	0	4.336265	6.254298	-3.554386
100	1	0	2.757899	5.998243	-2.799991
101	1	0	5.375763	4.593326	-5.057588
102	1	0	5.197178	3.303390	-3.850632
103	1	0	4.557961	3.083037	-5.485542
104	6	0	-3.358867	-4.436628	-4.226489
105	6	0	-2.695636	-5.157678	-5.424986

106	6	0	-3.665820	-5.489982	-3.144957
107	6	0	-4.700005	-3.814239	-4.683097
108	1	0	-2.484880	-4.467014	-6.248244
109	1	0	-1.747607	-5.620080	-5.126810
110	1	0	-3.354202	-5.946391	-5.810243
111	1	0	-4.162729	-5.046908	-2.274224
112	1	0	-4.336265	-6.254298	-3.554386
113	1	0	-2.757899	-5.998243	-2.799991
114	1	0	-5.375763	-4.593326	-5.057588
115	1	0	-5.197178	-3.303390	-3.850632
116	1	0	-4.557961	-3.083037	-5.485542
117	7	0	0.262826	5.223735	3.282359
118	7	0	-0.262826	-5.223735	3.282359
119	6	0	0.682738	4.200863	4.159603
120	6	0	0.942665	4.519656	5.510618
121	6	0	0.848508	2.861729	3.695486
122	6	0	1.392831	3.542982	6.388444
123	1	0	0.806984	5.531095	5.871603
124	6	0	1.330906	1.914206	4.623936
125	6	0	1.603600	2.231662	5.947949
126	1	0	1.591758	3.812974	7.422488
127	1	0	1.514906	0.902653	4.277256
128	1	0	1.975068	1.473366	6.631648
129	6	0	-0.682738	-4.200863	4.159603
130	6	0	-0.942665	-4.519656	5.510618
131	6	0	-0.848508	-2.861729	3.695486
132	6	0	-1.392831	-3.542982	6.388444
133	1	0	-0.806984	-5.531095	5.871603
134	6	0	-1.330906	-1.914206	4.623936
135	6	0	-1.603600	-2.231662	5.947949
136	1	0	-1.591758	-3.812974	7.422488
137	1	0	-1.514906	-0.902653	4.277256
138	1	0	-1.975068	-1.473366	6.631648
139	5	0	0.538168	2.560497	2.213922
140	5	0	-0.538168	-2.560497	2.213922
141	6	0	0.000000	6.530064	3.835801
142	6	0	-1.270303	6.824499	4.336965
143	6	0	1.010823	7.494166	3.867576
144	6	0	-1.527777	8.086843	4.873583
145	1	0	-2.043893	6.063238	4.303723
146	6	0	0.747991	8.755591	4.403664
147	1	0	1.992159	7.248810	3.472949
148	6	0	-0.520074	9.053020	4.907383
149	1	0	-2.516054	8.314783	5.263037
150	1	0	1.534000	9.505131	4.426418
151	1	0	-0.722741	10.035786	5.323850
152	6	0	0.000000	-6.530064	3.835801
153	6	0	1.270303	-6.824499	4.336965
154	6	0	-1.010823	-7.494166	3.867576
155	6	0	1.527777	-8.086843	4.873583
156	1	0	2.043893	-6.063238	4.303723
157	6	0	-0.747991	-8.755591	4.403664
158	1	0	-1.992159	-7.248810	3.472949
159	6	0	0.520074	-9.053020	4.907383
160	1	0	2.516054	-8.314783	5.263037
161	1	0	-1.534000	-9.505131	4.426418
162	1	0	0.722741	-10.035786	5.323850

ω-DABNA-4TBP (S_0, C_2 symmetry, Gaussian)

E(B3LYP/6–31G(d)) = −3462.50841829 hartree

Center Number	Atomic Number	Atomic Type	Coordinates (Angstroms) X	Y	Z
1	5	0	0.000000	0.000000	-2.078766
2	7	0	-0.608350	-2.352781	-0.567154
3	7	0	0.608350	2.352781	-0.567154
4	6	0	0.323011	1.179010	0.157178
5	6	0	0.000000	0.000000	-0.562640
6	6	0	-0.323011	-1.179010	0.157178
7	6	0	-0.362281	-1.193999	1.580073
8	6	0	0.000000	0.000000	2.221447
9	6	0	0.362281	1.193999	1.580073
10	1	0	0.000000	0.000000	3.305363
11	6	0	1.090686	2.274973	-1.908151
12	6	0	1.968110	3.271015	-2.383356
13	6	0	0.757929	1.170297	-2.725318
14	6	0	2.487494	3.238892	-3.675404
15	1	0	2.256907	4.067267	-1.712430
16	6	0	1.295712	1.155357	-4.030765
17	6	0	2.120191	2.159450	-4.506076
18	1	0	2.507353	2.094619	-5.518886
19	6	0	-1.090686	-2.274973	-1.908151
20	6	0	-0.757929	-1.170297	-2.725318
21	6	0	-1.968110	-3.271015	-2.383356
22	6	0	-1.295712	-1.155357	-4.030765
23	6	0	-2.487494	-3.238892	-3.675404
24	1	0	-2.256907	-4.067267	-1.712430
25	6	0	-2.120191	-2.159450	-4.506076
26	1	0	-2.507353	-2.094619	-5.518886
27	6	0	0.396323	3.625316	0.041200
28	6	0	0.540182	3.746337	1.443707
29	6	0	0.013864	4.724428	-0.730308
30	6	0	0.396665	5.026801	2.035953
31	6	0	-0.165437	5.979700	-0.127875
32	1	0	-0.131506	4.622318	-1.797725
33	6	0	0.044050	6.134260	1.246587
34	1	0	-0.115643	7.104630	1.696626
35	6	0	-0.396323	-3.625316	0.041200
36	6	0	-0.540182	-3.746337	1.443707
37	6	0	-0.013864	-4.724428	-0.730308
38	6	0	-0.396665	-5.026801	2.035953
39	6	0	0.165437	-5.979700	-0.127875
40	1	0	0.131506	-4.622318	-1.797725
41	6	0	-0.044050	-6.134260	1.246587
42	1	0	0.115643	-7.104630	1.696626
43	1	0	1.078972	0.304189	-4.670199
44	1	0	-1.078972	-0.304189	-4.670199
45	6	0	3.449640	4.318789	-4.202557
46	6	0	2.813884	5.004205	-5.436064
47	6	0	3.753501	5.405967	-3.154318
48	6	0	4.787686	3.660322	-4.615500
49	1	0	2.605880	4.288928	-6.238659
50	1	0	1.869654	5.492465	-5.169278
51	1	0	3.490492	5.768933	-5.837031
52	1	0	4.240212	4.990348	-2.264667
53	1	0	4.433505	6.150289	-3.584094
54	1	0	2.847264	5.932273	-2.834334
55	1	0	5.480613	4.417516	-5.002608

56	1	0	5.263524	3.170381	-3.758405
57	1	0	4.648652	2.905694	-5.396487
58	6	0	-3.449640	-4.318789	-4.202557
59	6	0	-2.813884	-5.004205	-5.436064
60	6	0	-3.753501	-5.405967	-3.154318
61	6	0	-4.787686	-3.660322	-4.615500
62	1	0	-2.605880	-4.288928	-6.238659
63	1	0	-1.869654	-5.492465	-5.169278
64	1	0	-3.490492	-5.768933	-5.837031
65	1	0	-4.240212	-4.990348	-2.264667
66	1	0	-4.433505	-6.150289	-3.584094
67	1	0	-2.847264	-5.932273	-2.834334
68	1	0	-5.480613	-4.417516	-5.002608
69	1	0	-5.263524	-3.170381	-3.758405
70	1	0	-4.648652	-2.905694	-5.396487
71	7	0	0.578859	5.165847	3.420760
72	7	0	-0.578859	-5.165847	3.420760
73	6	0	0.982153	4.111123	4.264867
74	6	0	1.297523	4.389414	5.613697
75	6	0	1.073187	2.774974	3.777630
76	6	0	1.728386	3.376780	6.457843
77	1	0	1.218803	5.398623	5.997695
78	6	0	1.541388	1.786932	4.672597
79	6	0	1.868407	2.064112	5.991047
80	1	0	1.972085	3.618979	7.489404
81	1	0	1.669827	0.775532	4.299957
82	6	0	-0.982153	-4.111123	4.264867
83	6	0	-1.297523	-4.389414	5.613697
84	6	0	-1.073187	-2.774974	3.777630
85	6	0	-1.728386	-3.376780	6.457843
86	1	0	-1.218803	-5.398623	5.997695
87	6	0	-1.541388	-1.786932	4.672597
88	6	0	-1.868407	-2.064112	5.991047
89	1	0	-1.972085	-3.618979	7.489404
90	1	0	-1.669827	-0.775532	4.299957
91	5	0	0.704477	2.510067	2.305990
92	5	0	-0.704477	-2.510067	2.305990
93	6	0	0.384803	6.471672	3.999226
94	6	0	-0.860919	6.817928	4.528681
95	6	0	1.438677	7.389664	4.026635
96	6	0	-1.051162	8.082955	5.086952
97	1	0	-1.669563	6.093849	4.498344
98	6	0	1.244220	8.653972	4.584995
99	1	0	2.401120	7.104859	3.611952
100	6	0	0.000000	9.001869	5.115685
101	1	0	-2.020903	8.350008	5.497640
102	1	0	2.065054	9.365547	4.606411
103	6	0	-0.384803	-6.471672	3.999226
104	6	0	0.860919	-6.817928	4.528681
105	6	0	-1.438677	-7.389664	4.026635
106	6	0	1.051162	-8.082955	5.086952
107	1	0	1.669563	-6.093849	4.498344
108	6	0	-1.244220	-8.653972	4.584995
109	1	0	-2.401120	-7.104859	3.611952
110	6	0	0.000000	-9.001869	5.115685
111	1	0	2.020903	-8.350008	5.497640
112	1	0	-2.065054	-9.365547	4.606411
113	1	0	-0.149886	9.986341	5.550257
114	1	0	2.228954	1.279055	6.649578
115	1	0	-2.228954	-1.279055	6.649578

116	1	0	0.149886	-9.986341	5.550257
117	6	0	-0.578520	7.144905	-0.952802
118	6	0	-0.075611	8.429779	-0.706277
119	6	0	-1.489203	7.003642	-2.013709
120	6	0	-0.463316	9.522869	-1.482088
121	1	0	0.649434	8.576660	0.089578
122	6	0	-1.873029	8.097006	-2.782822
123	1	0	-1.922066	6.029111	-2.221966
124	6	0	-1.371678	9.387699	-2.540379
125	1	0	-0.034815	10.491981	-1.250818
126	1	0	-2.587741	7.937069	-3.585684
127	6	0	0.578520	-7.144905	-0.952802
128	6	0	0.075611	-8.429779	-0.706277
129	6	0	1.489203	-7.003642	-2.013709
130	6	0	0.463316	-9.522869	-1.482088
131	1	0	-0.649434	-8.576660	0.089578
132	6	0	1.873029	-8.097006	-2.782822
133	1	0	1.922066	-6.029111	-2.221966
134	6	0	1.371678	-9.387699	-2.540379
135	1	0	0.034815	-10.491981	-1.250818
136	1	0	2.587741	-7.937069	-3.585684
137	6	0	1.823627	-10.568997	-3.417053
138	6	0	1.164670	-11.896380	-2.996583
139	6	0	1.446790	-10.290267	-4.891684
140	6	0	3.357824	-10.736604	-3.304386
141	1	0	1.417153	-12.168559	-1.965392
142	1	0	0.072811	-11.855199	-3.082244
143	1	0	1.516367	-12.705268	-3.646953
144	1	0	1.919041	-9.376553	-5.267362
145	1	0	1.769969	-11.120365	-5.531958
146	1	0	0.362548	-10.176230	-5.004065
147	1	0	3.697598	-11.571547	-3.929592
148	1	0	3.891272	-9.837747	-3.630671
149	1	0	3.654328	-10.943608	-2.269785
150	6	0	-1.823627	10.568997	-3.417053
151	6	0	-1.164670	11.896380	-2.996583
152	6	0	-1.446790	10.290267	-4.891684
153	6	0	-3.357824	10.736604	-3.304386
154	1	0	-1.417153	12.168559	-1.965392
155	1	0	-0.072811	11.855199	-3.082244
156	1	0	-1.516367	12.705268	-3.646953
157	1	0	-1.919041	9.376553	-5.267362
158	1	0	-1.769969	11.120365	-5.531958
159	1	0	-0.362548	10.176230	-5.004065
160	1	0	-3.697598	11.571547	-3.929592
161	1	0	-3.891272	9.837747	-3.630671
162	1	0	-3.654328	10.943608	-2.269785

ω-DABNA-4TBP (S_1, C_2 symmetry, Gaussian)
E(B3LYP/6–31G(d)) = −3462.50708616 hartree

Center Number	Atomic Number	Atomic Type	Coordinates (Angstroms) X	Y	Z
1	5	0	0.000000	0.000000	-2.037885
2	7	0	-0.575056	-2.356202	-0.524369
3	7	0	0.575056	2.356202	-0.524369
4	6	0	0.300114	1.180721	0.206815

5	6	0	0.000000	0.000000	-0.521260
6	6	0	-0.300114	-1.180721	0.206815
7	6	0	-0.331631	-1.203741	1.634537
8	6	0	0.000000	0.000000	2.287266
9	6	0	0.331631	1.203741	1.634537
10	1	0	0.000000	0.000000	3.368862
11	6	0	1.089533	2.274900	-1.856705
12	6	0	1.987766	3.263807	-2.302419
13	6	0	0.750834	1.174556	-2.682235
14	6	0	2.510541	3.252944	-3.595660
15	1	0	2.287045	4.038885	-1.610789
16	6	0	1.287994	1.185742	-3.988146
17	6	0	2.122260	2.194272	-4.442975
18	1	0	2.505382	2.144009	-5.458585
19	6	0	-1.089533	-2.274900	-1.856705
20	6	0	-0.750834	-1.174556	-2.682235
21	6	0	-1.987766	-3.263807	-2.302419
22	6	0	-1.287994	-1.185742	-3.988146
23	6	0	-2.510541	-3.252944	-3.595660
24	1	0	-2.287045	-4.038885	-1.610789
25	6	0	-2.122260	-2.194272	-4.442975
26	1	0	-2.505382	-2.144009	-5.458585
27	6	0	0.363295	3.625717	0.064861
28	6	0	0.479266	3.756105	1.469952
29	6	0	-0.005806	4.721199	-0.729424
30	6	0	0.335256	5.051614	2.033627
31	6	0	-0.197631	5.986506	-0.154360
32	1	0	-0.137390	4.596392	-1.796242
33	6	0	-0.004129	6.150797	1.227369
34	1	0	-0.173589	7.123703	1.667686
35	6	0	-0.363295	-3.625717	0.064861
36	6	0	-0.479266	-3.756105	1.469952
37	6	0	0.005806	-4.721199	-0.729424
38	6	0	-0.335256	-5.051614	2.033627
39	6	0	0.197631	-5.986506	-0.154360
40	1	0	0.137390	-4.596392	-1.796242
41	6	0	0.004129	-6.150797	1.227369
42	1	0	0.173589	-7.123703	1.667686
43	1	0	1.065827	0.351038	-4.647159
44	1	0	-1.065827	-0.351038	-4.647159
45	6	0	3.487964	4.327951	-4.102194
46	6	0	2.862241	5.050595	-5.319574
47	6	0	3.809332	5.386879	-3.030485
48	6	0	4.815750	3.659324	-4.531819
49	1	0	2.640399	4.355634	-6.136333
50	1	0	1.926825	5.548836	-5.040397
51	1	0	3.550712	5.812052	-5.706743
52	1	0	4.289105	4.943555	-2.150377
53	1	0	4.501160	6.129887	-3.443396
54	1	0	2.911226	5.920080	-2.698877
55	1	0	5.519379	4.413469	-4.905708
56	1	0	5.285938	3.144713	-3.686129
57	1	0	4.663339	2.922686	-5.327488
58	6	0	-3.487964	-4.327951	-4.102194
59	6	0	-2.862241	-5.050595	-5.319574
60	6	0	-3.809332	-5.386879	-3.030485
61	6	0	-4.815750	-3.659324	-4.531819
62	1	0	-2.640399	-4.355634	-6.136333
63	1	0	-1.926825	-5.548836	-5.040397
64	1	0	-3.550712	-5.812052	-5.706743

65	1	0	-4.289105	-4.943555	-2.150377
66	1	0	-4.501160	-6.129887	-3.443396
67	1	0	-2.911226	-5.920080	-2.698877
68	1	0	-5.519379	-4.413469	-4.905708
69	1	0	-5.285938	-3.144713	-3.686129
70	1	0	-4.663339	-2.922686	-5.327488
71	7	0	0.506628	5.211682	3.416683
72	7	0	-0.506628	-5.211682	3.416683
73	6	0	0.885978	4.171367	4.288802
74	6	0	1.174994	4.476834	5.637596
75	6	0	0.980877	2.824155	3.823244
76	6	0	1.583814	3.479138	6.511581
77	1	0	1.092656	5.493720	5.999320
78	6	0	1.423577	1.854308	4.748638
79	6	0	1.724017	2.158446	6.069769
80	1	0	1.805413	3.738624	7.543658
81	1	0	1.554015	0.834690	4.401455
82	6	0	-0.885978	-4.171367	4.288802
83	6	0	-1.174994	-4.476834	5.637596
84	6	0	-0.980877	-2.824155	3.823244
85	6	0	-1.583814	-3.479138	6.511581
86	1	0	-1.092656	-5.493720	5.999320
87	6	0	-1.423577	-1.854308	4.748638
88	6	0	-1.724017	-2.158446	6.069769
89	1	0	-1.805413	-3.738624	7.543658
90	1	0	-1.554015	-0.834690	4.401455
91	5	0	0.645070	2.537561	2.344914
92	5	0	-0.645070	-2.537561	2.344914
93	6	0	0.337001	6.535214	3.965785
94	6	0	-0.903820	6.916517	4.481563
95	6	0	1.409975	7.430515	3.975566
96	6	0	-1.069729	8.197532	5.010514
97	1	0	-1.726791	6.208432	4.464029
98	6	0	1.238863	8.710444	4.505072
99	1	0	2.367601	7.116978	3.570841
100	6	0	0.000000	9.095093	5.022928
101	1	0	-2.035376	8.493400	5.410789
102	1	0	2.073851	9.405517	4.513661
103	6	0	-0.337001	-6.535214	3.965785
104	6	0	0.903820	-6.916517	4.481563
105	6	0	-1.409975	-7.430515	3.975566
106	6	0	1.069729	-8.197532	5.010514
107	1	0	1.726791	-6.208432	4.464029
108	6	0	-1.238863	-8.710444	4.505072
109	1	0	-2.367601	-7.116978	3.570841
110	6	0	0.000000	-9.095093	5.022928
111	1	0	2.035376	-8.493400	5.410789
112	1	0	-2.073851	-9.405517	4.513661
113	1	0	-0.131336	10.091866	5.434722
114	1	0	2.063084	1.382672	6.750682
115	1	0	-2.063084	-1.382672	6.750682
116	1	0	0.131336	-10.091866	5.434722
117	6	0	-0.594341	7.137575	-0.999598
118	6	0	-0.142038	8.437651	-0.726307
119	6	0	-1.440877	6.975777	-2.111675
120	6	0	-0.514789	9.520421	-1.522698
121	1	0	0.535301	8.605149	0.106652
122	6	0	-1.808415	8.059032	-2.901522
123	1	0	-1.839746	5.992266	-2.344138
124	6	0	-1.357151	9.363280	-2.631725

125	1	0	-0.125874	10.500227	-1.267404
126	1	0	-2.472450	7.880596	-3.743108
127	6	0	0.594341	-7.137575	-0.999598
128	6	0	0.142038	-8.437651	-0.726307
129	6	0	1.440877	-6.975777	-2.111675
130	6	0	0.514789	-9.520421	-1.522698
131	1	0	-0.535301	-8.605149	0.106652
132	6	0	1.808415	-8.059032	-2.901522
133	1	0	1.839746	-5.992266	-2.344138
134	6	0	1.357151	-9.363280	-2.631725
135	1	0	0.125874	-10.500227	-1.267404
136	1	0	2.472450	-7.880596	-3.743108
137	6	0	1.790976	-10.532857	-3.532291
138	6	0	1.189168	-11.876187	-3.078186
139	6	0	1.329152	-10.265000	-4.984704
140	6	0	3.332870	-10.661675	-3.502313
141	1	0	1.504100	-12.141667	-2.062533
142	1	0	0.093566	-11.861980	-3.104180
143	1	0	1.524867	-12.676355	-3.747551
144	1	0	1.757106	-9.339734	-5.384403
145	1	0	1.638979	-11.086931	-5.641955
146	1	0	0.237909	-10.178778	-5.039070
147	1	0	3.659971	-11.487395	-4.146339
148	1	0	3.824914	-9.749230	-3.854968
149	1	0	3.689422	-10.861423	-2.485376
150	6	0	-1.790976	10.532857	-3.532291
151	6	0	-1.189168	11.876187	-3.078186
152	6	0	-1.329152	10.265000	-4.984704
153	6	0	-3.332870	10.661675	-3.502313
154	1	0	-1.504100	12.141667	-2.062533
155	1	0	-0.093566	11.861980	-3.104180
156	1	0	-1.524867	12.676355	-3.747551
157	1	0	-1.757106	9.339734	-5.384403
158	1	0	-1.638979	11.086931	-5.641955
159	1	0	-0.237909	10.178778	-5.039070
160	1	0	-3.659971	11.487395	-4.146339
161	1	0	-3.824914	9.749230	-3.854968
162	1	0	-3.689422	10.861423	-2.485376

ω-DABNA-4CzP (S_0, C_2 symmetry, Gaussian)
E(B3LYP/6–31G(d)) = −4180.5435047 hartree

Center Number	Atomic Number	Atomic Type	Coordinates (Angstroms) X	Y	Z
1	5	0	0.000000	0.000000	-1.382093
2	7	0	-0.885934	-2.262459	0.129920
3	7	0	0.885934	2.262459	0.129920
4	6	0	0.461701	1.131552	0.854486
5	6	0	0.000000	0.000000	0.134480
6	6	0	-0.461701	-1.131552	0.854486
7	6	0	-0.501729	-1.142306	2.277374
8	6	0	0.000000	0.000000	2.918936
9	6	0	0.501729	1.142306	2.277374
10	1	0	0.000000	0.000000	4.002762
11	6	0	1.356899	2.126774	-1.211033
12	6	0	2.349741	3.008551	-1.684279
13	6	0	0.893060	1.070882	-2.028522
14	6	0	2.863115	2.913787	-2.975831

15	1	0	2.733430	3.762649	-1.012113
16	6	0	1.425921	0.991489	-3.333671
17	6	0	2.367057	1.888018	-3.807521
18	1	0	2.744354	1.776960	-4.819976
19	6	0	-1.356899	-2.126774	-1.211033
20	6	0	-0.893060	-1.070882	-2.028522
21	6	0	-2.349741	-3.008551	-1.684279
22	6	0	-1.425921	-0.991489	-3.333671
23	6	0	-2.863115	-2.913787	-2.975831
24	1	0	-2.733430	-3.762649	-1.012113
25	6	0	-2.367057	-1.888018	-3.807521
26	1	0	-2.744354	-1.776960	-4.819976
27	6	0	0.826417	3.550769	0.737067
28	6	0	0.980509	3.655590	2.139741
29	6	0	0.579235	4.687197	-0.036265
30	6	0	0.990368	4.944257	2.731696
31	6	0	0.549816	5.954381	0.566322
32	1	0	0.426089	4.601836	-1.104081
33	6	0	0.774299	6.085536	1.940639
34	1	0	0.730048	7.067984	2.390651
35	6	0	-0.826417	-3.550769	0.737067
36	6	0	-0.980509	-3.655590	2.139741
37	6	0	-0.579235	-4.687197	-0.036265
38	6	0	-0.990368	-4.944257	2.731696
39	6	0	-0.549816	-5.954381	0.566322
40	1	0	-0.426089	-4.601836	-1.104081
41	6	0	-0.774299	-6.085536	1.940639
42	1	0	-0.730048	-7.067984	2.390651
43	1	0	1.107908	0.173498	-3.974007
44	1	0	-1.107908	-0.173498	-3.974007
45	6	0	3.951889	3.867177	-3.500697
46	6	0	3.407505	4.626957	-4.734370
47	6	0	4.386349	4.907277	-2.450781
48	6	0	5.198966	3.048914	-3.912740
49	1	0	3.114874	3.943853	-5.538568
50	1	0	2.529699	5.227103	-4.468607
51	1	0	4.173435	5.303542	-5.132741
52	1	0	4.816394	4.433729	-1.560892
53	1	0	5.153786	5.562267	-2.878477
54	1	0	3.551710	5.541617	-2.131677
55	1	0	5.980642	3.715346	-4.297456
56	1	0	5.609307	2.502651	-3.055860
57	1	0	4.969173	2.318591	-4.695364
58	6	0	-3.951889	-3.867177	-3.500697
59	6	0	-3.407505	-4.626957	-4.734370
60	6	0	-4.386349	-4.907277	-2.450781
61	6	0	-5.198966	-3.048914	-3.912740
62	1	0	-3.114874	-3.943853	-5.538568
63	1	0	-2.529699	-5.227103	-4.468607
64	1	0	-4.173435	-5.303542	-5.132741
65	1	0	-4.816394	-4.433729	-1.560892
66	1	0	-5.153786	-5.562267	-2.878477
67	1	0	-3.551710	-5.541617	-2.131677
68	1	0	-5.980642	-3.715346	-4.297456
69	1	0	-5.609307	-2.502651	-3.055860
70	1	0	-4.969173	-2.318591	-4.695364
71	7	0	1.186068	5.062064	4.115646
72	7	0	-1.186068	-5.062064	4.115646
73	6	0	1.459116	3.967121	4.961348
74	6	0	1.801345	4.207289	6.310789

75	6	0	1.392414	2.629349	4.474815
76	6	0	2.107183	3.151389	7.156419
77	1	0	1.841287	5.218930	6.694277
78	6	0	1.738591	1.593391	5.371305
79	6	0	2.092688	1.831055	6.690348
80	1	0	2.374901	3.363702	8.188545
81	1	0	1.748125	0.573686	4.999379
82	6	0	-1.459116	-3.967121	4.961348
83	6	0	-1.801345	-4.207289	6.310789
84	6	0	-1.392414	-2.629349	4.474815
85	6	0	-2.107183	-3.151389	7.156419
86	1	0	-1.841287	-5.218930	6.694277
87	6	0	-1.738591	-1.593391	5.371305
88	6	0	-2.092688	-1.831055	6.690348
89	1	0	-2.374901	-3.363702	8.188545
90	1	0	-1.748125	-0.573686	4.999379
91	5	0	0.996747	2.408680	3.003314
92	5	0	-0.996747	-2.408680	3.003314
93	6	0	1.154231	6.383286	4.691716
94	6	0	-0.040083	6.883147	5.216825
95	6	0	2.314359	7.162669	4.719893
96	6	0	-0.072455	8.163606	5.771551
97	1	0	-0.932656	6.265470	5.186328
98	6	0	2.277615	8.442834	5.274800
99	1	0	3.235074	6.758777	4.309316
100	6	0	1.084981	8.944436	5.801018
101	1	0	-1.002236	8.550388	6.179253
102	1	0	3.180727	9.046461	5.297337
103	6	0	-1.154231	-6.383286	4.691716
104	6	0	0.040083	-6.883147	5.216825
105	6	0	-2.314359	-7.162669	4.719893
106	6	0	0.072455	-8.163606	5.771551
107	1	0	0.932656	-6.265470	5.186328
108	6	0	-2.277615	-8.442834	5.274800
109	1	0	-3.235074	-6.758777	4.309316
110	6	0	-1.084981	-8.944436	5.801018
111	1	0	1.002236	-8.550388	6.179253
112	1	0	-3.180727	-9.046461	5.297337
113	1	0	1.057909	9.940924	6.233107
114	1	0	2.356616	1.009490	7.350089
115	1	0	-2.356616	-1.009490	7.350089
116	1	0	-1.057909	-9.940924	6.233107
117	6	0	0.278446	7.159687	-0.259430
118	6	0	0.937577	8.375403	-0.009146
119	6	0	-0.645821	7.123181	-1.317595
120	6	0	0.694300	9.504368	-0.786081
121	1	0	1.679474	8.427410	0.782263
122	6	0	-0.909901	8.252396	-2.087306
123	1	0	-1.195474	6.207854	-1.516101
124	6	0	-0.236824	9.453531	-1.830929
125	1	0	1.239123	10.424796	-0.600535
126	1	0	-1.653498	8.214760	-2.877264
127	6	0	-0.278446	-7.159687	-0.259430
128	6	0	-0.937577	-8.375403	-0.009146
129	6	0	0.645821	-7.123181	-1.317595
130	6	0	-0.694300	-9.504368	-0.786081
131	1	0	-1.679474	-8.427410	0.782263
132	6	0	0.909901	-8.252396	-2.087306
133	1	0	1.195474	-6.207854	-1.516101
134	6	0	0.236824	-9.453531	-1.830929

135	1	0	-1.239123	-10.424796	-0.600535
136	1	0	1.653498	-8.214760	-2.877264
137	7	0	-0.494678	10.604960	-2.620122
138	7	0	0.494678	-10.604960	-2.620122
139	6	0	-0.404720	10.679281	-4.015558
140	6	0	-0.735194	11.995023	-4.429477
141	6	0	-0.025072	9.697069	-4.934674
142	6	0	-0.702337	12.318620	-5.790944
143	6	0	0.000000	10.045891	-6.283358
144	1	0	0.246773	8.697775	-4.611281
145	6	0	-0.338306	11.340957	-6.712204
146	1	0	-0.954006	13.322641	-6.122787
147	1	0	0.290371	9.298411	-7.016752
148	1	0	-0.310769	11.579869	-7.771539
149	6	0	0.404720	-10.679281	-4.015558
150	6	0	0.735194	-11.995023	-4.429477
151	6	0	0.025072	-9.697069	-4.934674
152	6	0	0.702337	-12.318620	-5.790944
153	6	0	0.000000	-10.045891	-6.283358
154	1	0	-0.246773	-8.697775	-4.611281
155	6	0	0.338306	-11.340957	-6.712204
156	1	0	0.954006	-13.322641	-6.122787
157	1	0	-0.290371	-9.298411	-7.016752
158	1	0	0.310769	-11.579869	-7.771539
159	6	0	-0.880794	11.860742	-2.135932
160	6	0	-1.040479	12.748023	-3.230950
161	6	0	-1.132762	12.267133	-0.822520
162	6	0	-1.439707	14.068890	-2.995918
163	6	0	-1.529646	13.586784	-0.616757
164	1	0	-1.029944	11.578536	0.009571
165	6	0	-1.678883	14.483269	-1.688921
166	1	0	-1.565678	14.760112	-3.825287
167	1	0	-1.730384	13.926079	0.395948
168	1	0	-1.988108	15.506268	-1.494222
169	6	0	0.880794	-11.860742	-2.135932
170	6	0	1.040479	-12.748023	-3.230950
171	6	0	1.132762	-12.267133	-0.822520
172	6	0	1.439707	-14.068890	-2.995918
173	6	0	1.529646	-13.586784	-0.616757
174	1	0	1.029944	-11.578536	0.009571
175	6	0	1.678883	-14.483269	-1.688921
176	1	0	1.565678	-14.760112	-3.825287
177	1	0	1.730384	-13.926079	0.395948
178	1	0	1.988108	-15.506268	-1.494222

ω-DABNA-4CzP (S_1, C_2 symmetry, Gaussian)
E(B3LYP/6–31G(d)) = −4180.54221169 hartree

Center Number	Atomic Number	Atomic Type	Coordinates (Angstroms) X	Y	Z
1	5	0	0.000000	0.000000	-1.330942
2	7	0	-0.793639	-2.291761	0.182570
3	7	0	0.793639	2.291761	0.182570
4	6	0	0.409677	1.147937	0.912232
5	6	0	0.000000	0.000000	0.184929
6	6	0	-0.409677	-1.147937	0.912232
7	6	0	-0.445199	-1.166721	2.340215
8	6	0	0.000000	0.000000	2.991525

9	6	0	0.445199	1.166721	2.340215
10	1	0	0.000000	0.000000	4.073238
11	6	0	1.293107	2.165657	-1.150406
12	6	0	2.275575	3.070528	-1.599628
13	6	0	0.855374	1.100824	-1.975658
14	6	0	2.793790	3.010029	-2.892605
15	1	0	2.645698	3.815293	-0.909118
16	6	0	1.389470	1.061467	-3.281993
17	6	0	2.310855	1.989741	-3.738796
18	1	0	2.687489	1.903769	-4.754268
19	6	0	-1.293107	-2.165657	-1.150406
20	6	0	-0.855374	-1.100824	-1.975658
21	6	0	-2.275575	-3.070528	-1.599628
22	6	0	-1.389470	-1.061467	-3.281993
23	6	0	-2.793790	-3.010029	-2.892605
24	1	0	-2.645698	-3.815293	-0.909118
25	6	0	-2.310855	-1.989741	-3.738796
26	1	0	-2.687489	-1.903769	-4.754268
27	6	0	0.703184	3.576235	0.775381
28	6	0	0.840152	3.692158	2.179883
29	6	0	0.430070	4.700081	-0.015074
30	6	0	0.824035	4.994958	2.745705
31	6	0	0.363076	5.978505	0.562263
32	1	0	0.280573	4.588273	-1.080995
33	6	0	0.584085	6.122025	1.942663
34	1	0	0.509044	7.104902	2.386919
35	6	0	-0.703184	-3.576235	0.775381
36	6	0	-0.840152	-3.692158	2.179883
37	6	0	-0.430070	-4.700081	-0.015074
38	6	0	-0.824035	-4.994958	2.745705
39	6	0	-0.363076	-5.978505	0.562263
40	1	0	-0.280573	-4.588273	-1.080995
41	6	0	-0.584085	-6.122025	1.942663
42	1	0	-0.509044	-7.104902	2.386919
43	1	0	1.092054	0.249558	-3.939677
44	1	0	-1.092054	-0.249558	-3.939677
45	6	0	3.865792	3.990045	-3.400729
46	6	0	3.308601	4.764930	-4.619250
47	6	0	4.283339	5.016317	-2.330500
48	6	0	5.126190	3.201196	-3.829440
49	1	0	3.024814	4.092449	-5.435616
50	1	0	2.422299	5.346709	-4.341099
51	1	0	4.063556	5.460183	-5.006500
52	1	0	4.721261	4.532137	-1.450119
53	1	0	5.039803	5.692409	-2.744669
54	1	0	3.438127	5.630182	-1.999179
55	1	0	5.895974	3.887229	-4.203741
56	1	0	5.547176	2.646432	-2.983255
57	1	0	4.906998	2.481164	-4.624679
58	6	0	-3.865792	-3.990045	-3.400729
59	6	0	-3.308601	-4.764930	-4.619250
60	6	0	-4.283339	-5.016317	-2.330500
61	6	0	-5.126190	-3.201196	-3.829440
62	1	0	-3.024814	-4.092449	-5.435616
63	1	0	-2.422299	-5.346709	-4.341099
64	1	0	-4.063556	-5.460183	-5.006500
65	1	0	-4.721261	-4.532137	-1.450119
66	1	0	-5.039803	-5.692409	-2.744669
67	1	0	-3.438127	-5.630182	-1.999179
68	1	0	-5.895974	-3.887229	-4.203741

69	1	0	-5.547176	-2.646432	-2.983255
70	1	0	-4.906998	-2.481164	-4.624679
71	7	0	1.019585	5.135595	4.127118
72	7	0	-1.019585	-5.135595	4.127118
73	6	0	1.298737	4.061428	4.996428
74	6	0	1.622906	4.335167	6.343868
75	6	0	1.258021	2.711883	4.529281
76	6	0	1.934631	3.300804	7.215249
77	1	0	1.643546	5.354747	6.706708
78	6	0	1.606408	1.701813	5.451936
79	6	0	1.941141	1.973196	6.771916
80	1	0	2.185602	3.535795	8.246407
81	1	0	1.634320	0.674643	5.103592
82	6	0	-1.298737	-4.061428	4.996428
83	6	0	-1.622906	-4.335167	6.343868
84	6	0	-1.258021	-2.711883	4.529281
85	6	0	-1.934631	-3.300804	7.215249
86	1	0	-1.643546	-5.354747	6.706708
87	6	0	-1.606408	-1.701813	5.451936
88	6	0	-1.941141	-1.973196	6.771916
89	1	0	-2.185602	-3.535795	8.246407
90	1	0	-1.634320	-0.674643	5.103592
91	5	0	0.890329	2.462466	3.052365
92	5	0	-0.890329	-2.462466	3.052365
93	6	0	0.988404	6.469481	4.677182
94	6	0	-0.203413	6.974250	5.202422
95	6	0	2.146536	7.251592	4.677154
96	6	0	-0.234486	8.265734	5.731188
97	1	0	-1.093968	6.353112	5.192729
98	6	0	2.110045	8.542503	5.206465
99	1	0	3.064470	6.842494	4.265779
100	6	0	0.920578	9.050574	5.733761
101	1	0	-1.161834	8.657772	6.139245
102	1	0	3.011120	9.149455	5.207891
103	6	0	-0.988404	-6.469481	4.677182
104	6	0	0.203413	-6.974250	5.202422
105	6	0	-2.146536	-7.251592	4.677154
106	6	0	0.234486	-8.265734	5.731188
107	1	0	1.093968	-6.353112	5.192729
108	6	0	-2.110045	-8.542503	5.206465
109	1	0	-3.064470	-6.842494	4.265779
110	6	0	-0.920578	-9.050574	5.733761
111	1	0	1.161834	-8.657772	6.139245
112	1	0	-3.011120	-9.149455	5.207891
113	1	0	0.894088	10.055557	6.145595
114	1	0	2.204743	1.166946	7.450747
115	1	0	-2.204743	-1.166946	7.450747
116	1	0	-0.894088	-10.055557	6.145595
117	6	0	0.070846	7.160745	-0.279050
118	6	0	0.634657	8.418503	0.009035
119	6	0	-0.781658	7.073164	-1.396426
120	6	0	0.370711	9.531193	-0.782651
121	1	0	1.324156	8.517368	0.842198
122	6	0	-1.064228	8.184751	-2.182950
123	1	0	-1.266165	6.129442	-1.628569
124	6	0	-0.486469	9.426535	-1.886011
125	1	0	0.844622	10.483040	-0.563392
126	1	0	-1.752814	8.102112	-3.018165
127	6	0	-0.070846	-7.160745	-0.279050
128	6	0	-0.634657	-8.418503	0.009035

129	6	0	0.781658	-7.073164	-1.396426
130	6	0	-0.370711	-9.531193	-0.782651
131	1	0	-1.324156	-8.517368	0.842198
132	6	0	1.064228	-8.184751	-2.182950
133	1	0	1.266165	-6.129442	-1.628569
134	6	0	0.486469	-9.426535	-1.886011
135	1	0	-0.844622	-10.483040	-0.563392
136	1	0	1.752814	-8.102112	-3.018165
137	7	0	-0.765431	10.561887	-2.690996
138	7	0	0.765431	-10.561887	-2.690996
139	6	0	-0.589804	10.646955	-4.077442
140	6	0	-0.975037	11.940665	-4.513177
141	6	0	-0.090326	9.693482	-4.969232
142	6	0	-0.874545	12.270011	-5.869933
143	6	0	0.000000	10.047452	-6.313717
144	1	0	0.222185	8.712322	-4.627621
145	6	0	-0.390776	11.320093	-6.764795
146	1	0	-1.166917	13.257278	-6.218333
147	1	0	0.383753	9.322105	-7.026144
148	1	0	-0.309826	11.563619	-7.820355
149	6	0	0.589804	-10.646955	-4.077442
150	6	0	0.975037	-11.940665	-4.513177
151	6	0	0.090326	-9.693482	-4.969232
152	6	0	0.874545	-12.270011	-5.869933
153	6	0	0.000000	-10.047452	-6.313717
154	1	0	-0.222185	-8.712322	-4.627621
155	6	0	0.390776	-11.320093	-6.764795
156	1	0	1.166917	-13.257278	-6.218333
157	1	0	-0.383753	-9.322105	-7.026144
158	1	0	0.309826	-11.563619	-7.820355
159	6	0	-1.260431	11.789098	-2.234052
160	6	0	-1.403982	12.668855	-3.337477
161	6	0	-1.622183	12.174136	-0.939899
162	6	0	-1.899445	13.961316	-3.129321
163	6	0	-2.113279	13.465620	-0.760783
164	1	0	-1.530640	11.489819	-0.102986
165	6	0	-2.248305	14.355129	-1.840727
166	1	0	-2.014389	14.646406	-3.965379
167	1	0	-2.400323	13.787996	0.236574
168	1	0	-2.632821	15.356134	-1.666862
169	6	0	1.260431	-11.789098	-2.234052
170	6	0	1.403982	-12.668855	-3.337477
171	6	0	1.622183	-12.174136	-0.939899
172	6	0	1.899445	-13.961316	-3.129321
173	6	0	2.113279	-13.465620	-0.760783
174	1	0	1.530640	-11.489819	-0.102986
175	6	0	2.248305	-14.355129	-1.840727
176	1	0	2.014389	-14.646406	-3.965379
177	1	0	2.400323	-13.787996	0.236574
178	1	0	2.632821	-15.356134	-1.666862

ω-DABNA-4CNP (S_0, C_2 symmetry, Gaussian)
E(B3LYP/6–31G(d)) = −3332.48639743 hartree

Center Number	Atomic Number	Atomic Type	Coordinates (Angstroms) X	Y	Z
1	5	0	0.000000	0.000000	-2.339377
2	7	0	-0.597648	-2.354661	-0.827282

3	7	0	0.597648	2.354661	-0.827282
4	6	0	0.316879	1.179980	-0.102393
5	6	0	0.000000	0.000000	-0.822392
6	6	0	-0.316879	-1.179980	-0.102393
7	6	0	-0.354079	-1.196170	1.320600
8	6	0	0.000000	0.000000	1.962487
9	6	0	0.354079	1.196170	1.320600
10	1	0	0.000000	0.000000	3.046327
11	6	0	1.082651	2.278506	-2.168634
12	6	0	1.958637	3.276454	-2.641280
13	6	0	0.753132	1.173322	-2.985807
14	6	0	2.480520	3.245697	-3.932811
15	1	0	2.246727	4.072148	-1.969142
16	6	0	1.292360	1.160037	-4.290644
17	6	0	2.115618	2.166062	-4.764105
18	1	0	2.504429	2.102209	-5.776192
19	6	0	-1.082651	-2.278506	-2.168634
20	6	0	-0.753132	-1.173322	-2.985807
21	6	0	-1.958637	-3.276454	-2.641280
22	6	0	-1.292360	-1.160037	-4.290644
23	6	0	-2.480520	-3.245697	-3.932811
24	1	0	-2.246727	-4.072148	-1.969142
25	6	0	-2.115618	-2.166062	-4.764105
26	1	0	-2.504429	-2.102209	-5.776192
27	6	0	0.375527	3.624635	-0.221567
28	6	0	0.510737	3.749682	1.181353
29	6	0	-0.013611	4.720026	-0.996531
30	6	0	0.355824	5.029019	1.773739
31	6	0	-0.203795	5.972402	-0.393062
32	1	0	-0.153305	4.614668	-2.064379
33	6	0	0.000000	6.133947	0.981048
34	1	0	-0.168269	7.103080	1.430530
35	6	0	-0.375527	-3.624635	-0.221567
36	6	0	-0.510737	-3.749682	1.181353
37	6	0	0.013611	-4.720026	-0.996531
38	6	0	-0.355824	-5.029019	1.773739
39	6	0	0.203795	-5.972402	-0.393062
40	1	0	0.153305	-4.614668	-2.064379
41	6	0	0.000000	-6.133947	0.981048
42	1	0	0.168269	-7.103080	1.430530
43	1	0	1.078457	0.308921	-4.930979
44	1	0	-1.078457	-0.308921	-4.930979
45	6	0	3.443693	4.325553	-4.457859
46	6	0	2.812504	5.008914	-5.694853
47	6	0	3.743228	5.413931	-3.409683
48	6	0	4.783610	3.666791	-4.864547
49	1	0	2.608827	4.292955	-6.497827
50	1	0	1.866613	5.497006	-5.433532
51	1	0	3.490081	5.773686	-6.093501
52	1	0	4.227534	5.000063	-2.517873
53	1	0	4.423543	6.158557	-3.837955
54	1	0	2.835261	5.939981	-3.093678
55	1	0	5.477413	4.423886	-5.249800
56	1	0	5.256484	3.178047	-4.005145
57	1	0	4.647966	2.911529	-5.645435
58	6	0	-3.443693	-4.325553	-4.457859
59	6	0	-2.812504	-5.008914	-5.694853
60	6	0	-3.743228	-5.413931	-3.409683
61	6	0	-4.783610	-3.666791	-4.864547
62	1	0	-2.608827	-4.292955	-6.497827

63	1	0	-1.866613	-5.497006	-5.433532
64	1	0	-3.490081	-5.773686	-6.093501
65	1	0	-4.227534	-5.000063	-2.517873
66	1	0	-4.423543	-6.158557	-3.837955
67	1	0	-2.835261	-5.939981	-3.093678
68	1	0	-5.477413	-4.423886	-5.249800
69	1	0	-5.256484	-3.178047	-4.005145
70	1	0	-4.647966	-2.911529	-5.645435
71	7	0	0.531752	5.171393	3.156927
72	7	0	-0.531752	-5.171393	3.156927
73	6	0	0.942249	4.120438	4.003440
74	6	0	1.249563	4.403266	5.352848
75	6	0	1.047601	2.785054	3.517456
76	6	0	1.687423	3.395503	6.199094
77	1	0	1.159209	5.411843	5.735755
78	6	0	1.522528	1.801963	4.414758
79	6	0	1.842239	2.083837	5.733702
80	1	0	1.925092	3.640649	7.231229
81	1	0	1.662588	0.791653	4.043446
82	6	0	-0.942249	-4.120438	4.003440
83	6	0	-1.249563	-4.403266	5.352848
84	6	0	-1.047601	-2.785054	3.517456
85	6	0	-1.687423	-3.395503	6.199094
86	1	0	-1.159209	-5.411843	5.735755
87	6	0	-1.522528	-1.801963	4.414758
88	6	0	-1.842239	-2.083837	5.733702
89	1	0	-1.925092	-3.640649	7.231229
90	1	0	-1.662588	-0.791653	4.043446
91	5	0	0.684455	2.514814	2.046444
92	5	0	-0.684455	-2.514814	2.046444
93	6	0	0.325665	6.477317	3.733018
94	6	0	-0.926332	6.816545	4.251968
95	6	0	1.374191	7.401224	3.766288
96	6	0	-1.128505	8.081951	4.805333
97	1	0	-1.730333	6.087440	4.217973
98	6	0	1.167554	8.665704	4.320027
99	1	0	2.342078	7.121051	3.361089
100	6	0	-0.083113	9.007273	4.839705
101	1	0	-2.102808	8.344128	5.208049
102	1	0	1.983931	9.382096	4.346906
103	6	0	-0.325665	-6.477317	3.733018
104	6	0	0.926332	-6.816545	4.251968
105	6	0	-1.374191	-7.401224	3.766288
106	6	0	1.128505	-8.081951	4.805333
107	1	0	1.730333	-6.087440	4.217973
108	6	0	-1.167554	-8.665704	4.320027
109	1	0	-2.342078	-7.121051	3.361089
110	6	0	0.083113	-9.007273	4.839705
111	1	0	2.102808	-8.344128	5.208049
112	1	0	-1.983931	-9.382096	4.346906
113	1	0	-0.242388	9.991797	5.270556
114	1	0	2.208704	1.303310	6.394212
115	1	0	-2.208704	-1.303310	6.394212
116	1	0	0.242388	-9.991797	5.270556
117	6	0	-0.626032	7.132778	-1.219241
118	6	0	-0.121829	8.421560	-0.967606
119	6	0	-1.541496	6.974515	-2.275434
120	6	0	-0.512582	9.511174	-1.736234
121	1	0	0.603547	8.564980	-0.172821
122	6	0	-1.940300	8.055995	-3.051453

123	1	0	-1.964438	5.994617	-2.473688
124	6	0	-1.427639	9.337199	-2.787877
125	1	0	-0.108070	10.498068	-1.535196
126	1	0	-2.654561	7.919315	-3.857118
127	6	0	0.626032	-7.132778	-1.219241
128	6	0	0.121829	-8.421560	-0.967606
129	6	0	1.541496	-6.974515	-2.275434
130	6	0	0.512582	-9.511174	-1.736234
131	1	0	-0.603547	-8.564980	-0.172821
132	6	0	1.940300	-8.055995	-3.051453
133	1	0	1.964438	-5.994617	-2.473688
134	6	0	1.427639	-9.337199	-2.787877
135	1	0	0.108070	-10.498068	-1.535196
136	1	0	2.654561	-7.919315	-3.857118
137	6	0	1.834034	-10.457146	-3.584189
138	7	0	2.163177	-11.367343	-4.230234
139	6	0	-1.834034	10.457146	-3.584189
140	7	0	-2.163177	11.367343	-4.230234

ω-DABNA-4CNP (S_1, C_2 symmetry, Gaussian)
E(B3LYP/6–31G(d)) = −3332.48447957 hartree

Center Number	Atomic Number	Atomic Type	Coordinates (Angstroms) X	Y	Z
1	5	0	0.000000	0.000000	-2.322995
2	7	0	-0.544102	-2.363636	-0.808919
3	7	0	0.544102	2.363636	-0.808919
4	6	0	0.284416	1.186535	-0.084317
5	6	0	0.000000	0.000000	-0.807966
6	6	0	-0.284416	-1.186535	-0.084317
7	6	0	-0.323174	-1.205927	1.344418
8	6	0	0.000000	0.000000	1.991275
9	6	0	0.323174	1.205927	1.344418
10	1	0	0.000000	0.000000	3.073450
11	6	0	1.038384	2.299022	-2.144482
12	6	0	1.899144	3.317366	-2.606278
13	6	0	0.726679	1.189475	-2.967815
14	6	0	2.415441	3.311004	-3.899824
15	1	0	2.180579	4.105427	-1.922575
16	6	0	1.257145	1.205366	-4.275373
17	6	0	2.058860	2.234339	-4.739875
18	1	0	2.441380	2.189058	-5.755374
19	6	0	-1.038384	-2.299022	-2.144482
20	6	0	-0.726679	-1.189475	-2.967815
21	6	0	-1.899144	-3.317366	-2.606278
22	6	0	-1.257145	-1.205366	-4.275373
23	6	0	-2.415441	-3.311004	-3.899824
24	1	0	-2.180579	-4.105427	-1.922575
25	6	0	-2.058860	-2.234339	-4.739875
26	1	0	-2.441380	-2.189058	-5.755374
27	6	0	0.314258	3.633657	-0.205907
28	6	0	0.459510	3.756805	1.197264
29	6	0	-0.097325	4.715242	-0.985263
30	6	0	0.308074	5.048459	1.768767
31	6	0	-0.293449	5.984439	-0.404023
32	1	0	-0.248766	4.588316	-2.048973
33	6	0	-0.064917	6.144105	0.973973
34	1	0	-0.240379	7.109605	1.427191

35	6	0	-0.314258	-3.633657	-0.205907
36	6	0	-0.459510	-3.756805	1.197264
37	6	0	0.097325	-4.715242	-0.985263
38	6	0	-0.308074	-5.048459	1.768767
39	6	0	0.293449	-5.984439	-0.404023
40	1	0	0.248766	-4.588316	-2.048973
41	6	0	0.064917	-6.144105	0.973973
42	1	0	0.240379	-7.109605	1.427191
43	1	0	1.056751	0.360059	-4.927320
44	1	0	-1.056751	-0.360059	-4.927320
45	6	0	3.358787	4.410671	-4.418044
46	6	0	2.706328	5.102070	-5.639586
47	6	0	3.651236	5.487749	-3.356313
48	6	0	4.704087	3.776517	-4.845638
49	1	0	2.504665	4.394531	-6.450661
50	1	0	1.757093	5.574650	-5.362767
51	1	0	3.370183	5.880729	-6.034043
52	1	0	4.148533	5.067751	-2.474432
53	1	0	4.318005	6.247527	-3.778832
54	1	0	2.738930	5.997373	-3.026363
55	1	0	5.383227	4.548918	-5.226200
56	1	0	5.191954	3.282660	-3.997609
57	1	0	4.572702	3.030749	-5.636456
58	6	0	-3.358787	-4.410671	-4.418044
59	6	0	-2.706328	-5.102070	-5.639586
60	6	0	-3.651236	-5.487749	-3.356313
61	6	0	-4.704087	-3.776517	-4.845638
62	1	0	-2.504665	-4.394531	-6.450661
63	1	0	-1.757093	-5.574650	-5.362767
64	1	0	-3.370183	-5.880729	-6.034043
65	1	0	-4.148533	-5.067751	-2.474432
66	1	0	-4.318005	-6.247527	-3.778832
67	1	0	-2.738930	-5.997373	-3.026363
68	1	0	-5.383227	-4.548918	-5.226200
69	1	0	-5.191954	-3.282660	-3.997609
70	1	0	-4.572702	-3.030749	-5.636456
71	7	0	0.502697	5.204139	3.148395
72	7	0	-0.502697	-5.204139	3.148395
73	6	0	0.898167	4.161840	4.009189
74	6	0	1.204668	4.462365	5.355632
75	6	0	0.991879	2.815949	3.537568
76	6	0	1.627126	3.462420	6.219349
77	1	0	1.124903	5.477618	5.722160
78	6	0	1.448697	1.843244	4.453084
79	6	0	1.764731	2.143030	5.771053
80	1	0	1.862527	3.718248	7.249114
81	1	0	1.579393	0.825258	4.100883
82	6	0	-0.898167	-4.161840	4.009189
83	6	0	-1.204668	-4.462365	5.355632
84	6	0	-0.991879	-2.815949	3.537568
85	6	0	-1.627126	-3.462420	6.219349
86	1	0	-1.124903	-5.477618	5.722160
87	6	0	-1.448697	-1.843244	4.453084
88	6	0	-1.764731	-2.143030	5.771053
89	1	0	-1.862527	-3.718248	7.249114
90	1	0	-1.579393	-0.825258	4.100883
91	5	0	0.641705	2.537811	2.062909
92	5	0	-0.641705	-2.537811	2.062909
93	6	0	0.334455	6.525798	3.705480
94	6	0	-0.899030	6.896658	4.245684

95	6	0	1.401529	7.427929	3.695032
96	6	0	-1.063480	8.175973	4.779271
97	1	0	-1.717508	6.183144	4.243339
98	6	0	1.231421	8.706020	4.229458
99	1	0	2.353518	7.122011	3.271535
100	6	0	0.000000	9.081028	4.771651
101	1	0	-2.023155	8.464747	5.198337
102	1	0	2.061295	9.407044	4.222795
103	6	0	-0.334455	-6.525798	3.705480
104	6	0	0.899030	-6.896658	4.245684
105	6	0	-1.401529	-7.427929	3.695032
106	6	0	1.063480	-8.175973	4.779271
107	1	0	1.717508	-6.183144	4.243339
108	6	0	-1.231421	-8.706020	4.229458
109	1	0	-2.353518	-7.122011	3.271535
110	6	0	0.000000	-9.081028	4.771651
111	1	0	2.023155	-8.464747	5.198337
112	1	0	-2.061295	-9.407044	4.222795
113	1	0	-0.130530	10.076427	5.186679
114	1	0	2.115371	1.366246	6.444655
115	1	0	-2.115371	-1.366246	6.444655
116	1	0	0.130530	-10.076427	5.186679
117	6	0	-0.720176	7.124889	-1.235580
118	6	0	-0.343861	8.447590	-0.911104
119	6	0	-1.521093	6.939588	-2.384520
120	6	0	-0.740086	9.525186	-1.687798
121	1	0	0.296966	8.627015	-0.053503
122	6	0	-1.923963	8.009888	-3.167926
123	1	0	-1.861310	5.942739	-2.647145
124	6	0	-1.538303	9.322538	-2.830676
125	1	0	-0.428295	10.531464	-1.425826
126	1	0	-2.549677	7.843928	-4.039268
127	6	0	0.720176	-7.124889	-1.235580
128	6	0	0.343861	-8.447590	-0.911104
129	6	0	1.521093	-6.939588	-2.384520
130	6	0	0.740086	-9.525186	-1.687798
131	1	0	-0.296966	-8.627015	-0.053503
132	6	0	1.923963	-8.009888	-3.167926
133	1	0	1.861310	-5.942739	-2.647145
134	6	0	1.538303	-9.322538	-2.830676
135	1	0	0.428295	-10.531464	-1.425826
136	1	0	2.549677	-7.843928	-4.039268
137	6	0	1.949569	-10.430015	-3.633977
138	7	0	2.283656	-11.333904	-4.289248
139	6	0	-1.949569	10.430015	-3.633977
140	7	0	-2.283656	11.333904	-4.289248

ω-DABNA (S_0, C_2 symmetry, Gaussian)
E(M06–2X/6–31G(d)) = −3719.28973054 hartree

Center Number	Atomic Number	Atomic Type	Coordinates (Angstroms) X	Y	Z
1	5	0	0.000000	0.000000	-2.155731
2	7	0	-0.412687	-2.389376	-0.645740
3	7	0	0.412687	2.389376	-0.645740
4	6	0	0.223659	1.199648	0.076166
5	6	0	0.000000	0.000000	-0.640433
6	6	0	-0.223659	-1.199648	0.076166

7	6	0	-0.257411	-1.215677	1.493858
8	6	0	0.000000	0.000000	2.133670
9	6	0	0.257411	1.215677	1.493858
10	1	0	0.000000	0.000000	3.218355
11	6	0	0.879019	2.353795	-1.989244
12	6	0	1.641195	3.431445	-2.476039
13	6	0	0.640962	1.235204	-2.806921
14	6	0	2.086428	3.477042	-3.787589
15	1	0	1.877474	4.243544	-1.803544
16	6	0	1.146641	1.276044	-4.121821
17	6	0	1.825422	2.370179	-4.617316
18	1	0	2.178099	2.364256	-5.644927
19	6	0	-0.879019	-2.353795	-1.989244
20	6	0	-0.640962	-1.235204	-2.806921
21	6	0	-1.641195	-3.431445	-2.476039
22	6	0	-1.146641	-1.276044	-4.121821
23	6	0	-2.086428	-3.477042	-3.787589
24	1	0	-1.877474	-4.243544	-1.803544
25	6	0	-1.825422	-2.370179	-4.617316
26	1	0	-2.178099	-2.364256	-5.644927
27	6	0	0.114347	3.640911	-0.040578
28	6	0	0.231877	3.769287	1.359322
29	6	0	-0.295613	4.716665	-0.823303
30	6	0	0.000000	5.035638	1.943591
31	6	0	-0.528594	5.959320	-0.220830
32	1	0	-0.419173	4.614301	-1.892820
33	6	0	-0.393898	6.124492	1.158571
34	1	0	-0.597909	7.092304	1.595720
35	6	0	-0.114347	-3.640911	-0.040578
36	6	0	-0.231877	-3.769287	1.359322
37	6	0	0.295613	-4.716665	-0.823303
38	6	0	0.000000	-5.035638	1.943591
39	6	0	0.528594	-5.959320	-0.220830
40	1	0	0.419173	-4.614301	-1.892820
41	6	0	0.393898	-6.124492	1.158571
42	1	0	0.597909	-7.092304	1.595720
43	1	0	1.012587	0.402793	-4.754189
44	1	0	-1.012587	-0.402793	-4.754189
45	7	0	-0.886874	7.065584	-1.017973
46	7	0	0.886874	-7.065584	-1.017973
47	6	0	-0.461488	8.374951	-0.675418
48	6	0	0.819777	8.607259	-0.162159
49	6	0	-1.325273	9.456730	-0.874209
50	6	0	1.216365	9.899264	0.163759
51	1	0	1.494591	7.769352	-0.016706
52	6	0	-0.912403	10.747501	-0.564103
53	1	0	-2.317667	9.273278	-1.274564
54	6	0	0.356488	10.976667	-0.037452
55	1	0	2.212625	10.064593	0.563128
56	1	0	-1.593956	11.577349	-0.724202
57	1	0	0.672760	11.984391	0.211546
58	6	0	0.461488	-8.374951	-0.675418
59	6	0	-0.819777	-8.607259	-0.162159
60	6	0	1.325273	-9.456730	-0.874209
61	6	0	-1.216365	-9.899264	0.163759
62	1	0	-1.494591	-7.769352	-0.016706
63	6	0	0.912403	-10.747501	-0.564103
64	1	0	2.317667	-9.273278	-1.274564
65	6	0	-0.356488	-10.976667	-0.037452
66	1	0	-2.212625	-10.064593	0.563128

67	1	0	1.593956	-11.577349	-0.724202
68	1	0	-0.672760	-11.984391	0.211546
69	6	0	-1.535138	6.869775	-2.266143
70	6	0	-2.666135	6.052278	-2.349953
71	6	0	-1.046034	7.489978	-3.419892
72	6	0	-3.288526	5.848469	-3.576230
73	1	0	-3.040648	5.574652	-1.449627
74	6	0	-1.686228	7.295269	-4.639844
75	1	0	-0.167666	8.124973	-3.349560
76	6	0	-2.805495	6.470696	-4.725566
77	1	0	-4.162699	5.207124	-3.630361
78	1	0	-1.297709	7.781918	-5.529404
79	1	0	-3.297514	6.314129	-5.679934
80	6	0	1.535138	-6.869775	-2.266143
81	6	0	2.666135	-6.052278	-2.349953
82	6	0	1.046034	-7.489978	-3.419892
83	6	0	3.288526	-5.848469	-3.576230
84	1	0	3.040648	-5.574652	-1.449627
85	6	0	1.686228	-7.295269	-4.639844
86	1	0	0.167666	-8.124973	-3.349560
87	6	0	2.805495	-6.470696	-4.725566
88	1	0	4.162699	-5.207124	-3.630361
89	1	0	1.297709	-7.781918	-5.529404
90	1	0	3.297514	-6.314129	-5.679934
91	6	0	2.831999	4.691884	-4.343848
92	6	0	2.016870	5.272735	-5.512942
93	6	0	3.008606	5.795901	-3.295317
94	6	0	4.220568	4.264551	-4.845898
95	1	0	1.884250	4.540716	-6.315434
96	1	0	1.024145	5.587323	-5.172376
97	1	0	2.531476	6.144636	-5.932944
98	1	0	3.611464	5.455874	-2.446474
99	1	0	3.521863	6.650398	-3.748329
100	1	0	2.041756	6.147526	-2.914796
101	1	0	4.756434	5.128032	-5.255899
102	1	0	4.816204	3.845772	-4.028307
103	1	0	4.149366	3.508300	-5.633538
104	6	0	-2.831999	-4.691884	-4.343848
105	6	0	-2.016870	-5.272735	-5.512942
106	6	0	-3.008606	-5.795901	-3.295317
107	6	0	-4.220568	-4.264551	-4.845898
108	1	0	-1.884250	-4.540716	-6.315434
109	1	0	-1.024145	-5.587323	-5.172376
110	1	0	-2.531476	-6.144636	-5.932944
111	1	0	-3.611464	-5.455874	-2.446474
112	1	0	-3.521863	-6.650398	-3.748329
113	1	0	-2.041756	-6.147526	-2.914796
114	1	0	-4.756434	-5.128032	-5.255899
115	1	0	-4.816204	-3.845772	-4.028307
116	1	0	-4.149366	-3.508300	-5.633538
117	7	0	0.146531	5.184466	3.324263
118	7	0	-0.146531	-5.184466	3.324263
119	6	0	0.596285	4.165876	4.179829
120	6	0	0.864465	4.475679	5.529015
121	6	0	0.788062	2.843854	3.706135
122	6	0	1.341405	3.499681	6.385191
123	1	0	0.713529	5.481397	5.901566
124	6	0	1.303064	1.895168	4.611173
125	6	0	1.579059	2.199131	5.931828
126	1	0	1.547061	3.762383	7.418902

127	1	0	1.515134	0.895376	4.244709
128	1	0	1.977849	1.445118	6.602309
129	6	0	-0.596285	-4.165876	4.179829
130	6	0	-0.864465	-4.475679	5.529015
131	6	0	-0.788062	-2.843854	3.706135
132	6	0	-1.341405	-3.499681	6.385191
133	1	0	-0.713529	-5.481397	5.901566
134	6	0	-1.303064	-1.895168	4.611173
135	6	0	-1.579059	-2.199131	5.931828
136	1	0	-1.547061	-3.762383	7.418902
137	1	0	-1.515134	-0.895376	4.244709
138	1	0	-1.977849	-1.445118	6.602309
139	5	0	0.474739	2.553673	2.224817
140	5	0	-0.474739	-2.553673	2.224817
141	6	0	-0.098009	6.484741	3.877988
142	6	0	-1.330222	6.766486	4.460819
143	6	0	0.892061	7.462619	3.808774
144	6	0	-1.571258	8.035238	4.981620
145	1	0	-2.085104	5.987069	4.499521
146	6	0	0.643531	8.732144	4.321829
147	1	0	1.842307	7.217770	3.343106
148	6	0	-0.586910	9.018262	4.910033
149	1	0	-2.530897	8.257236	5.437510
150	1	0	1.410033	9.498180	4.260936
151	1	0	-0.779353	10.008661	5.310183
152	6	0	0.098009	-6.484741	3.877988
153	6	0	1.330222	-6.766486	4.460819
154	6	0	-0.892061	-7.462619	3.808774
155	6	0	1.571258	-8.035238	4.981620
156	1	0	2.085104	-5.987069	4.499521
157	6	0	-0.643531	-8.732144	4.321829
158	1	0	-1.842307	-7.217770	3.343106
159	6	0	0.586910	-9.018262	4.910033
160	1	0	2.530897	-8.257236	5.437510
161	1	0	-1.410033	-9.498180	4.260936
162	1	0	0.779353	-10.008661	5.310183

ω-DABNA-4TBP (S_0, C_2 symmetry, Gaussian)
E(M06–2X/6–31G(d)) = −3461.08288738 hartree

Center Number	Atomic Number	Atomic Type	Coordinates (Angstroms) X	Y	Z
1	5	0	0.000000	0.000000	-2.065720
2	7	0	-0.562710	-2.356677	-0.555064
3	7	0	0.562710	2.356677	-0.555064
4	6	0	0.301317	1.181976	0.165441
5	6	0	0.000000	0.000000	-0.550939
6	6	0	-0.301317	-1.181976	0.165441
7	6	0	-0.342347	-1.194261	1.583675
8	6	0	0.000000	0.000000	2.224590
9	6	0	0.342347	1.194261	1.583675
10	1	0	0.000000	0.000000	3.309641
11	6	0	1.027307	2.294128	-1.897941
12	6	0	1.865521	3.317329	-2.378775
13	6	0	0.719774	1.191814	-2.715061
14	6	0	2.346006	3.313850	-3.679410
15	1	0	2.146785	4.116043	-1.706513
16	6	0	1.240514	1.191989	-4.024343

17	6	0	2.012356	2.227052	-4.509961
18	1	0	2.385525	2.186512	-5.529450
19	6	0	-1.027307	-2.294128	-1.897941
20	6	0	-0.719774	-1.191814	-2.715061
21	6	0	-1.865521	-3.317329	-2.378775
22	6	0	-1.240514	-1.191989	-4.024343
23	6	0	-2.346006	-3.313850	-3.679410
24	1	0	-2.146785	-4.116043	-1.706513
25	6	0	-2.012356	-2.227052	-4.509961
26	1	0	-2.385525	-2.186512	-5.529450
27	6	0	0.346976	3.623180	0.053772
28	6	0	0.490427	3.746317	1.450822
29	6	0	-0.032910	4.721596	-0.715234
30	6	0	0.341180	5.018938	2.045471
31	6	0	-0.199210	5.971244	-0.109662
32	1	0	-0.177826	4.625777	-1.784313
33	6	0	0.000000	6.129746	1.260707
34	1	0	-0.157876	7.102584	1.708950
35	6	0	-0.346976	-3.623180	0.053772
36	6	0	-0.490427	-3.746317	1.450822
37	6	0	0.032910	-4.721596	-0.715234
38	6	0	-0.341180	-5.018938	2.045471
39	6	0	0.199210	-5.971244	-0.109662
40	1	0	0.177826	-4.625777	-1.784313
41	6	0	0.000000	-6.129746	1.260707
42	1	0	0.157876	-7.102584	1.708950
43	1	0	1.048013	0.329810	-4.657060
44	1	0	-1.048013	-0.329810	-4.657060
45	6	0	3.224994	4.443171	-4.221482
46	6	0	2.518042	5.086069	-5.427253
47	6	0	3.479308	5.535962	-3.177749
48	6	0	4.580150	3.869916	-4.667939
49	1	0	2.340760	4.360007	-6.226430
50	1	0	1.551578	5.506085	-5.129547
51	1	0	3.133431	5.895088	-5.836622
52	1	0	4.017335	5.145480	-2.307505
53	1	0	4.092409	6.327206	-3.621201
54	1	0	2.544570	5.990887	-2.830712
55	1	0	5.215964	4.670102	-5.063201
56	1	0	5.099025	3.401948	-3.825167
57	1	0	4.461614	3.116204	-5.452217
58	6	0	-3.224994	-4.443171	-4.221482
59	6	0	-2.518042	-5.086069	-5.427253
60	6	0	-3.479308	-5.535962	-3.177749
61	6	0	-4.580150	-3.869916	-4.667939
62	1	0	-2.340760	-4.360007	-6.226430
63	1	0	-1.551578	-5.506085	-5.129547
64	1	0	-3.133431	-5.895088	-5.836622
65	1	0	-4.017335	-5.145480	-2.307505
66	1	0	-4.092409	-6.327206	-3.621201
67	1	0	-2.544570	-5.990887	-2.830712
68	1	0	-5.215964	-4.670102	-5.063201
69	1	0	-5.099025	-3.401948	-3.825167
70	1	0	-4.461614	-3.116204	-5.452217
71	7	0	0.510525	5.150971	3.425517
72	7	0	-0.510525	-5.150971	3.425517
73	6	0	0.901021	4.102332	4.272443
74	6	0	1.188348	4.383767	5.624399
75	6	0	1.008417	2.773897	3.789769
76	6	0	1.607996	3.373722	6.470547

77	1	0	1.094987	5.392620	6.007089
78	6	0	1.467666	1.787792	4.685299
79	6	0	1.767102	2.064494	6.006272
80	1	0	1.829962	3.614708	7.506164
81	1	0	1.615088	0.779715	4.309739
82	6	0	-0.901021	-4.102332	4.272443
83	6	0	-1.188348	-4.383767	5.624399
84	6	0	-1.008417	-2.773897	3.789769
85	6	0	-1.607996	-3.373722	6.470547
86	1	0	-1.094987	-5.392620	6.007089
87	6	0	-1.467666	-1.787792	4.685299
88	6	0	-1.767102	-2.064494	6.006272
89	1	0	-1.829962	-3.614708	7.506164
90	1	0	-1.615088	-0.779715	4.309739
91	5	0	0.662140	2.510896	2.312988
92	5	0	-0.662140	-2.510896	2.312988
93	6	0	0.343297	6.457694	3.993017
94	6	0	-0.890667	6.828393	4.519703
95	6	0	1.414924	7.347772	4.005567
96	6	0	-1.052294	8.099534	5.064886
97	1	0	-1.709389	6.115725	4.495430
98	6	0	1.248478	8.618041	4.549855
99	1	0	2.366404	7.032081	3.587907
100	6	0	0.015589	8.993986	5.079776
101	1	0	-2.013349	8.391475	5.476069
102	1	0	2.081789	9.313451	4.561685
103	6	0	-0.343297	-6.457694	3.993017
104	6	0	0.890667	-6.828393	4.519703
105	6	0	-1.414924	-7.347772	4.005567
106	6	0	1.052294	-8.099534	5.064886
107	1	0	1.709389	-6.115725	4.495430
108	6	0	-1.248478	-8.618041	4.549855
109	1	0	-2.366404	-7.032081	3.587907
110	6	0	-0.015589	-8.993986	5.079776
111	1	0	2.013349	-8.391475	5.476069
112	1	0	-2.081789	-9.313451	4.561685
113	1	0	-0.112889	9.984687	5.504170
114	1	0	2.122260	1.283104	6.669832
115	1	0	-2.122260	-1.283104	6.669832
116	1	0	0.112889	-9.984687	5.504170
117	6	0	-0.582907	7.140654	-0.940224
118	6	0	-0.050718	8.407058	-0.691450
119	6	0	-1.483802	7.009197	-2.004436
120	6	0	-0.403851	9.503208	-1.474815
121	1	0	0.668634	8.534797	0.112908
122	6	0	-1.833358	8.104710	-2.780278
123	1	0	-1.933791	6.041505	-2.207912
124	6	0	-1.302470	9.378974	-2.536439
125	1	0	0.043369	10.464666	-1.247029
126	1	0	-2.543865	7.962975	-3.590574
127	6	0	0.582907	-7.140654	-0.940224
128	6	0	0.050718	-8.407058	-0.691450
129	6	0	1.483802	-7.009197	-2.004436
130	6	0	0.403851	-9.503208	-1.474815
131	1	0	-0.668634	-8.534797	0.112908
132	6	0	1.833358	-8.104710	-2.780278
133	1	0	1.933791	-6.041505	-2.207912
134	6	0	1.302470	-9.378974	-2.536439
135	1	0	-0.043369	-10.464666	-1.247029
136	1	0	2.543865	-7.962975	-3.590574

137	6	0	1.718200	-10.556863	-3.420634
138	6	0	1.033347	-11.862036	-3.003255
139	6	0	1.338555	-10.257323	-4.880611
140	6	0	3.239989	-10.756376	-3.320285
141	1	0	1.289707	-12.140686	-1.975655
142	1	0	-0.056539	-11.787570	-3.080114
143	1	0	1.360308	-12.673613	-3.661240
144	1	0	1.836243	-9.355460	-5.249693
145	1	0	1.632703	-11.092289	-5.526507
146	1	0	0.258009	-10.110361	-4.977747
147	1	0	3.553249	-11.596826	-3.949885
148	1	0	3.785867	-9.867401	-3.650370
149	1	0	3.535487	-10.969866	-2.287976
150	6	0	-1.718200	10.556863	-3.420634
151	6	0	-1.033347	11.862036	-3.003255
152	6	0	-1.338555	10.257323	-4.880611
153	6	0	-3.239989	10.756376	-3.320285
154	1	0	-1.289707	12.140686	-1.975655
155	1	0	0.056539	11.787570	-3.080114
156	1	0	-1.360308	12.673613	-3.661240
157	1	0	-1.836243	9.355460	-5.249693
158	1	0	-1.632703	11.092289	-5.526507
159	1	0	-0.258009	10.110361	-4.977747
160	1	0	-3.553249	11.596826	-3.949885
161	1	0	-3.785867	9.867401	-3.650370
162	1	0	-3.535487	10.969866	-2.287976

ω-DABNA-4CzP (S_0, C_2 symmetry, Gaussian)
E(M06–2X/6–31G(d)) = −4178.87232596 hartree

Center Number	Atomic Number	Atomic Type	Coordinates (Angstroms) X	Y	Z
1	5	0	0.000000	0.000000	-1.292367
2	7	0	-0.734618	-2.312352	0.214643
3	7	0	0.734618	2.312352	0.214643
4	6	0	0.383414	1.154400	0.940908
5	6	0	0.000000	0.000000	0.224292
6	6	0	-0.383414	-1.154400	0.940908
7	6	0	-0.423913	-1.171058	2.360033
8	6	0	0.000000	0.000000	3.001210
9	6	0	0.423913	1.171058	2.360033
10	1	0	0.000000	0.000000	4.085369
11	6	0	1.189317	2.218614	-1.122383
12	6	0	2.081488	3.194899	-1.609319
13	6	0	0.804021	1.138518	-1.941698
14	6	0	2.538264	3.175316	-2.917735
15	1	0	2.424513	3.965144	-0.931676
16	6	0	1.307786	1.118793	-3.256271
17	6	0	2.129477	2.113564	-3.748178
18	1	0	2.482345	2.059198	-4.774127
19	6	0	-1.189317	-2.218614	-1.122383
20	6	0	-0.804021	-1.138518	-1.941698
21	6	0	-2.081488	-3.194899	-1.609319
22	6	0	-1.307786	-1.118793	-3.256271
23	6	0	-2.538264	-3.175316	-2.917735
24	1	0	-2.424513	-3.965144	-0.931676
25	6	0	-2.129477	-2.113564	-3.748178
26	1	0	-2.482345	-2.059198	-4.774127

27	6	0	0.591111	3.586470	0.817229
28	6	0	0.735838	3.700209	2.218382
29	6	0	0.274840	4.703603	0.037583
30	6	0	0.686761	4.997807	2.783808
31	6	0	0.212547	5.980493	0.608574
32	1	0	0.112345	4.590184	-1.027786
33	6	0	0.433575	6.126713	1.986506
34	1	0	0.348519	7.109032	2.433456
35	6	0	-0.591111	-3.586470	0.817229
36	6	0	-0.735838	-3.700209	2.218382
37	6	0	-0.274840	-4.703603	0.037583
38	6	0	-0.686761	-4.997807	2.783808
39	6	0	-0.212547	-5.980493	0.608574
40	1	0	-0.112345	-4.590184	-1.027786
41	6	0	-0.433575	-6.126713	1.986506
42	1	0	-0.348519	-7.109032	2.433456
43	1	0	1.057791	0.274933	-3.893420
44	1	0	-1.057791	-0.274933	-3.893420
45	6	0	3.462010	4.262739	-3.470374
46	6	0	2.747186	4.968093	-4.636344
47	6	0	3.814675	5.318280	-2.417089
48	6	0	4.765712	3.625124	-3.978134
49	1	0	2.496453	4.268260	-5.439345
50	1	0	1.818615	5.436532	-4.292216
51	1	0	3.391261	5.748743	-5.056296
52	1	0	4.354219	4.879486	-1.571081
53	1	0	4.460426	6.079514	-2.866343
54	1	0	2.920438	5.822659	-2.033349
55	1	0	5.430765	4.397602	-4.379917
56	1	0	5.287332	3.110272	-3.164960
57	1	0	4.577833	2.897417	-4.773228
58	6	0	-3.462010	-4.262739	-3.470374
59	6	0	-2.747186	-4.968093	-4.636344
60	6	0	-3.814675	-5.318280	-2.417089
61	6	0	-4.765712	-3.625124	-3.978134
62	1	0	-2.496453	-4.268260	-5.439345
63	1	0	-1.818615	-5.436532	-4.292216
64	1	0	-3.391261	-5.748743	-5.056296
65	1	0	-4.354219	-4.879486	-1.571081
66	1	0	-4.460426	-6.079514	-2.866343
67	1	0	-2.920438	-5.822659	-2.033349
68	1	0	-5.430765	-4.397602	-4.379917
69	1	0	-5.287332	-3.110272	-3.164960
70	1	0	-4.577833	-2.897417	-4.773228
71	7	0	0.870131	5.136965	4.156939
72	7	0	-0.870131	-5.136965	4.156939
73	6	0	1.152535	4.075726	5.034148
74	6	0	1.439836	4.367179	6.382607
75	6	0	1.152280	2.730175	4.568913
76	6	0	1.750026	3.345536	7.264396
77	1	0	1.429775	5.389760	6.738643
78	6	0	1.504078	1.733663	5.500823
79	6	0	1.796081	2.019531	6.824044
80	1	0	1.971605	3.587625	8.299096
81	1	0	1.574088	0.706785	5.156609
82	6	0	-1.152535	-4.075726	5.034148
83	6	0	-1.439836	-4.367179	6.382607
84	6	0	-1.152280	-2.730175	4.568913
85	6	0	-1.750026	-3.345536	7.264396
86	1	0	-1.429775	-5.389760	6.738643

87	6	0	-1.504078	-1.733663	5.500823
88	6	0	-1.796081	-2.019531	6.824044
89	1	0	-1.971605	-3.587625	8.299096
90	1	0	-1.574088	-0.706785	5.156609
91	5	0	0.820336	2.474324	3.087525
92	5	0	-0.820336	-2.474324	3.087525
93	6	0	0.827164	6.467673	4.698887
94	6	0	-0.366708	6.958124	5.218585
95	6	0	1.976949	7.252640	4.687111
96	6	0	-0.408458	8.251106	5.733711
97	1	0	-1.247002	6.322715	5.212740
98	6	0	1.928524	8.544998	5.202142
99	1	0	2.893692	6.841557	4.275108
100	6	0	0.737034	9.043998	5.725159
101	1	0	-1.337158	8.639116	6.139472
102	1	0	2.821780	9.161353	5.196013
103	6	0	-0.827164	-6.467673	4.698887
104	6	0	0.366708	-6.958124	5.218585
105	6	0	-1.976949	-7.252640	4.687111
106	6	0	0.408458	-8.251106	5.733711
107	1	0	1.247002	-6.322715	5.212740
108	6	0	-1.928524	-8.544998	5.202142
109	1	0	-2.893692	-6.841557	4.275108
110	6	0	-0.737034	-9.043998	5.725159
111	1	0	1.337158	-8.639116	6.139472
112	1	0	-2.821780	-9.161353	5.196013
113	1	0	0.701527	10.051834	6.126256
114	1	0	2.063342	1.223040	7.511269
115	1	0	-2.063342	-1.223040	7.511269
116	1	0	-0.701527	-10.051834	6.126256
117	6	0	-0.058316	7.160260	-0.238707
118	6	0	0.513214	8.406748	0.057103
119	6	0	-0.879053	7.068628	-1.373029
120	6	0	0.288883	9.512658	-0.751489
121	1	0	1.180066	8.500457	0.909248
122	6	0	-1.121171	8.172855	-2.177892
123	1	0	-1.368422	6.127723	-1.606045
124	6	0	-0.531748	9.402160	-1.875350
125	1	0	0.767061	10.463119	-0.534329
126	1	0	-1.784281	8.098213	-3.034525
127	6	0	0.058316	-7.160260	-0.238707
128	6	0	-0.513214	-8.406748	0.057103
129	6	0	0.879053	-7.068628	-1.373029
130	6	0	-0.288883	-9.512658	-0.751489
131	1	0	-1.180066	-8.500457	0.909248
132	6	0	1.121171	-8.172855	-2.177892
133	1	0	1.368422	-6.127723	-1.606045
134	6	0	0.531748	-9.402160	-1.875350
135	1	0	-0.767061	-10.463119	-0.534329
136	1	0	1.784281	-8.098213	-3.034525
137	7	0	-0.761678	10.524858	-2.704022
138	7	0	0.761678	-10.524858	-2.704022
139	6	0	-0.576142	10.566943	-4.083849
140	6	0	-0.902836	11.858048	-4.552284
141	6	0	-0.110325	9.570907	-4.944257
142	6	0	-0.783370	12.149776	-5.912706
143	6	0	0.000000	9.887548	-6.291305
144	1	0	0.163554	8.589075	-4.572450
145	6	0	-0.336590	11.160716	-6.776744
146	1	0	-1.033178	13.139213	-6.284989

147	1	0	0.358260	9.131362	-6.982966
148	1	0	-0.240301	11.371823	-7.836675
149	6	0	0.576142	-10.566943	-4.083849
150	6	0	0.902836	-11.858048	-4.552284
151	6	0	0.110325	-9.570907	-4.944257
152	6	0	0.783370	-12.149776	-5.912706
153	6	0	0.000000	-9.887548	-6.291305
154	1	0	-0.163554	-8.589075	-4.572450
155	6	0	0.336590	-11.160716	-6.776744
156	1	0	1.033178	-13.139213	-6.284989
157	1	0	-0.358260	-9.131362	-6.982966
158	1	0	0.240301	-11.371823	-7.836675
159	6	0	-1.203406	11.774977	-2.278338
160	6	0	-1.304930	12.631719	-3.395920
161	6	0	-1.553741	12.199025	-0.995170
162	6	0	-1.744334	13.945628	-3.220923
163	6	0	-1.988271	13.509368	-0.849250
164	1	0	-1.497584	11.525862	-0.146211
165	6	0	-2.079692	14.379542	-1.946645
166	1	0	-1.825652	14.614287	-4.073132
167	1	0	-2.267084	13.865752	0.137595
168	1	0	-2.421743	15.398014	-1.794879
169	6	0	1.203406	-11.774977	-2.278338
170	6	0	1.304930	-12.631719	-3.395920
171	6	0	1.553741	-12.199025	-0.995170
172	6	0	1.744334	-13.945628	-3.220923
173	6	0	1.988271	-13.509368	-0.849250
174	1	0	1.497584	-11.525862	-0.146211
175	6	0	2.079692	-14.379542	-1.946645
176	1	0	1.825652	-14.614287	-4.073132
177	1	0	2.267084	-13.865752	0.137595
178	1	0	2.421743	-15.398014	-1.794879

ω-DABNA-4CNP (S_0, C_2 symmetry, Gaussian)
E(M06–2X/6–31G(d)) = −3331.16063836 hartree

Center Number	Atomic Number	Atomic Type	Coordinates (Angstroms) X	Y	Z
1	5	0	0.000000	0.000000	-2.326312
2	7	0	-0.569942	-2.354198	-0.815462
3	7	0	0.569942	2.354198	-0.815462
4	6	0	0.304104	1.180622	-0.094244
5	6	0	0.000000	0.000000	-0.810767
6	6	0	-0.304104	-1.180622	-0.094244
7	6	0	-0.342835	-1.193880	1.323985
8	6	0	0.000000	0.000000	1.965386
9	6	0	0.342835	1.193880	1.323985
10	1	0	0.000000	0.000000	3.050353
11	6	0	1.037553	2.289197	-2.158373
12	6	0	1.883147	3.307058	-2.636327
13	6	0	0.724795	1.188722	-2.975437
14	6	0	2.368113	3.299805	-3.935769
15	1	0	2.169098	4.103413	-1.962952
16	6	0	1.248413	1.185381	-4.283477
17	6	0	2.028811	2.215225	-4.766723
18	1	0	2.404932	2.171701	-5.784883
19	6	0	-1.037553	-2.289197	-2.158373
20	6	0	-0.724795	-1.188722	-2.975437

21	6	0	-1.883147	-3.307058	-2.636327
22	6	0	-1.248413	-1.185381	-4.283477
23	6	0	-2.368113	-3.299805	-3.935769
24	1	0	-2.169098	-4.103413	-1.962952
25	6	0	-2.028811	-2.215225	-4.766723
26	1	0	-2.404932	-2.171701	-5.784883
27	6	0	0.353370	3.619885	-0.209612
28	6	0	0.487562	3.745966	1.187978
29	6	0	-0.023561	4.717752	-0.982655
30	6	0	0.335508	5.018556	1.782583
31	6	0	-0.192904	5.965416	-0.375856
32	1	0	-0.162482	4.619878	-2.052373
33	6	0	0.000000	6.129438	0.994209
34	1	0	-0.159698	7.102404	1.441550
35	6	0	-0.353370	-3.619885	-0.209612
36	6	0	-0.487562	-3.745966	1.187978
37	6	0	0.023561	-4.717752	-0.982655
38	6	0	-0.335508	-5.018556	1.782583
39	6	0	0.192904	-5.965416	-0.375856
40	1	0	0.162482	-4.619878	-2.052373
41	6	0	0.000000	-6.129438	0.994209
42	1	0	0.159698	-7.102404	1.441550
43	1	0	1.051916	0.324697	-4.916931
44	1	0	-1.051916	-0.324697	-4.916931
45	6	0	3.259563	4.420463	-4.475191
46	6	0	2.567216	5.065055	-5.688500
47	6	0	3.515197	5.514175	-3.432818
48	6	0	4.613358	3.834414	-4.909351
49	1	0	2.391643	4.338599	-6.487561
50	1	0	1.601090	5.492893	-5.400967
51	1	0	3.191502	5.868568	-6.094709
52	1	0	4.045295	5.122815	-2.558100
53	1	0	4.135803	6.300303	-3.874520
54	1	0	2.581082	5.976155	-3.093119
55	1	0	5.258394	4.628077	-5.302428
56	1	0	5.122203	3.364958	-4.061322
57	1	0	4.494424	3.079402	-5.692248
58	6	0	-3.259563	-4.420463	-4.475191
59	6	0	-2.567216	-5.065055	-5.688500
60	6	0	-3.515197	-5.514175	-3.432818
61	6	0	-4.613358	-3.834414	-4.909351
62	1	0	-2.391643	-4.338599	-6.487561
63	1	0	-1.601090	-5.492893	-5.400967
64	1	0	-3.191502	-5.868568	-6.094709
65	1	0	-4.045295	-5.122815	-2.558100
66	1	0	-4.135803	-6.300303	-3.874520
67	1	0	-2.581082	-5.976155	-3.093119
68	1	0	-5.258394	-4.628077	-5.302428
69	1	0	-5.122203	-3.364958	-4.061322
70	1	0	-4.494424	-3.079402	-5.692248
71	7	0	0.497815	5.152608	3.161254
72	7	0	-0.497815	-5.152608	3.161254
73	6	0	0.886563	4.104817	4.011239
74	6	0	1.164702	4.388447	5.364336
75	6	0	1.000135	2.776644	3.529895
76	6	0	1.582901	3.380239	6.213290
77	1	0	1.065232	5.397097	5.745899
78	6	0	1.457775	1.792216	4.428522
79	6	0	1.749185	2.071195	5.750547
80	1	0	1.798114	3.622327	7.249970

81	1	0	1.610693	0.784386	4.054565
82	6	0	-0.886563	-4.104817	4.011239
83	6	0	-1.164702	-4.388447	5.364336
84	6	0	-1.000135	-2.776644	3.529895
85	6	0	-1.582901	-3.380239	6.213290
86	1	0	-1.065232	-5.397097	5.745899
87	6	0	-1.457775	-1.792216	4.428522
88	6	0	-1.749185	-2.071195	5.750547
89	1	0	-1.798114	-3.622327	7.249970
90	1	0	-1.610693	-0.784386	4.054565
91	5	0	0.659444	2.510886	2.053264
92	5	0	-0.659444	-2.510886	2.053264
93	6	0	0.327717	6.461033	3.725795
94	6	0	-0.912079	6.836516	4.234955
95	6	0	1.402301	7.347311	3.749783
96	6	0	-1.076973	8.110202	4.773396
97	1	0	-1.732706	6.126320	4.202763
98	6	0	1.232487	8.619842	4.287884
99	1	0	2.358829	7.027044	3.347261
100	6	0	-0.006520	9.001386	4.799404
101	1	0	-2.042373	8.406531	5.170870
102	1	0	2.067849	9.312480	4.309551
103	6	0	-0.327717	-6.461033	3.725795
104	6	0	0.912079	-6.836516	4.234955
105	6	0	-1.402301	-7.347311	3.749783
106	6	0	1.076973	-8.110202	4.773396
107	1	0	1.732706	-6.126320	4.202763
108	6	0	-1.232487	-8.619842	4.287884
109	1	0	-2.358829	-7.027044	3.347261
110	6	0	0.006520	-9.001386	4.799404
111	1	0	2.042373	-8.406531	5.170870
112	1	0	-2.067849	-9.312480	4.309551
113	1	0	-0.137627	9.993848	5.218668
114	1	0	2.103706	1.291761	6.416651
115	1	0	-2.103706	-1.291761	6.416651
116	1	0	0.137627	-9.993848	5.218668
117	6	0	-0.577341	7.133636	-1.207719
118	6	0	-0.030644	8.398742	-0.955883
119	6	0	-1.488223	6.993325	-2.262868
120	6	0	-0.380117	9.494124	-1.731877
121	1	0	0.693044	8.516761	-0.155227
122	6	0	-1.845965	8.081362	-3.045401
123	1	0	-1.938433	6.024276	-2.454409
124	6	0	-1.291004	9.337991	-2.781666
125	1	0	0.052406	10.469847	-1.537887
126	1	0	-2.558826	7.968989	-3.855276
127	6	0	0.577341	-7.133636	-1.207719
128	6	0	0.030644	-8.398742	-0.955883
129	6	0	1.488223	-6.993325	-2.262868
130	6	0	0.380117	-9.494124	-1.731877
131	1	0	-0.693044	-8.516761	-0.155227
132	6	0	1.845965	-8.081362	-3.045401
133	1	0	1.938433	-6.024276	-2.454409
134	6	0	1.291004	-9.337991	-2.781666
135	1	0	-0.052406	-10.469847	-1.537887
136	1	0	2.558826	-7.968989	-3.855276
137	6	0	1.656667	-10.470643	-3.590189
138	7	0	1.949859	-11.382411	-4.240019
139	6	0	-1.656667	10.470643	-3.590189
140	7	0	-1.949859	11.382411	-4.240019

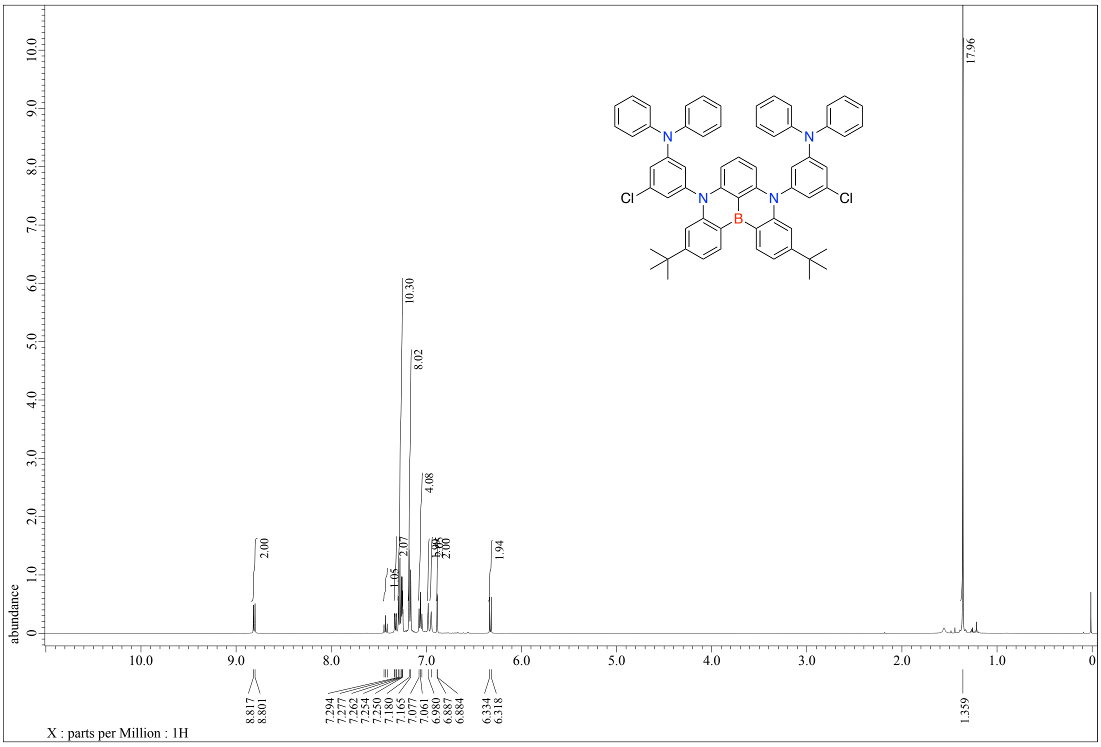

Figure S23. ^{1}H NMR spectrum of **b** in $CDCl_3$ at 25 °C.

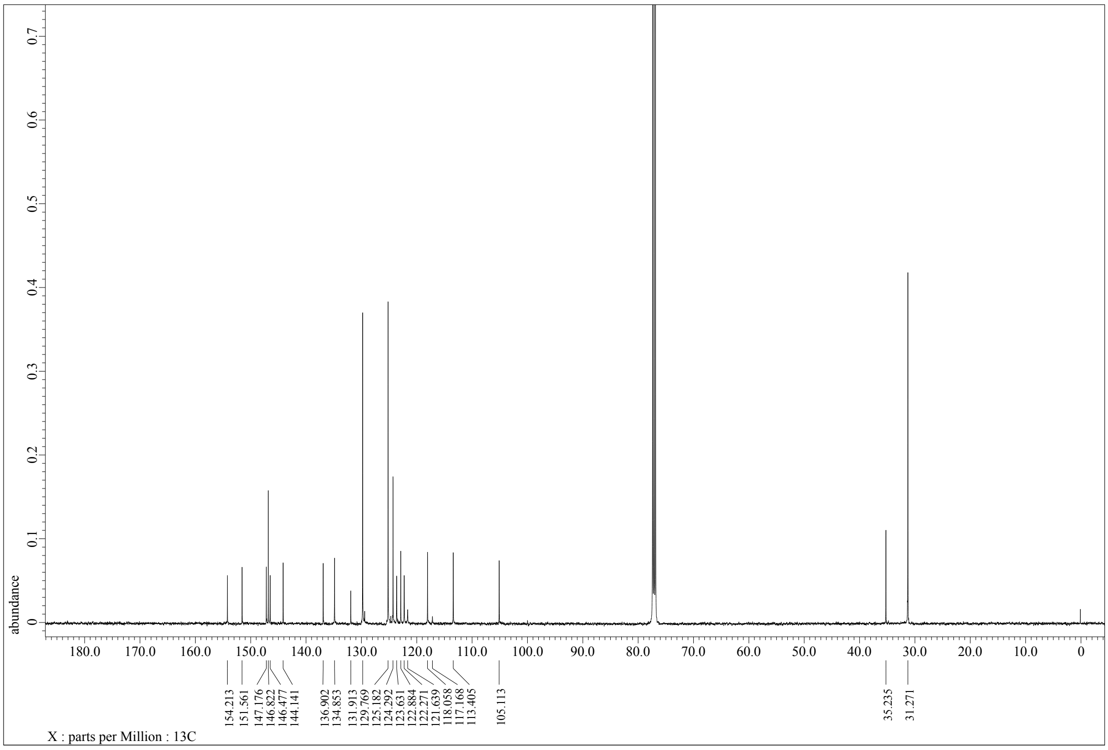

Figure S24. ^{13}C NMR spectrum of **b** in $CDCl_3$ at 25 °C.

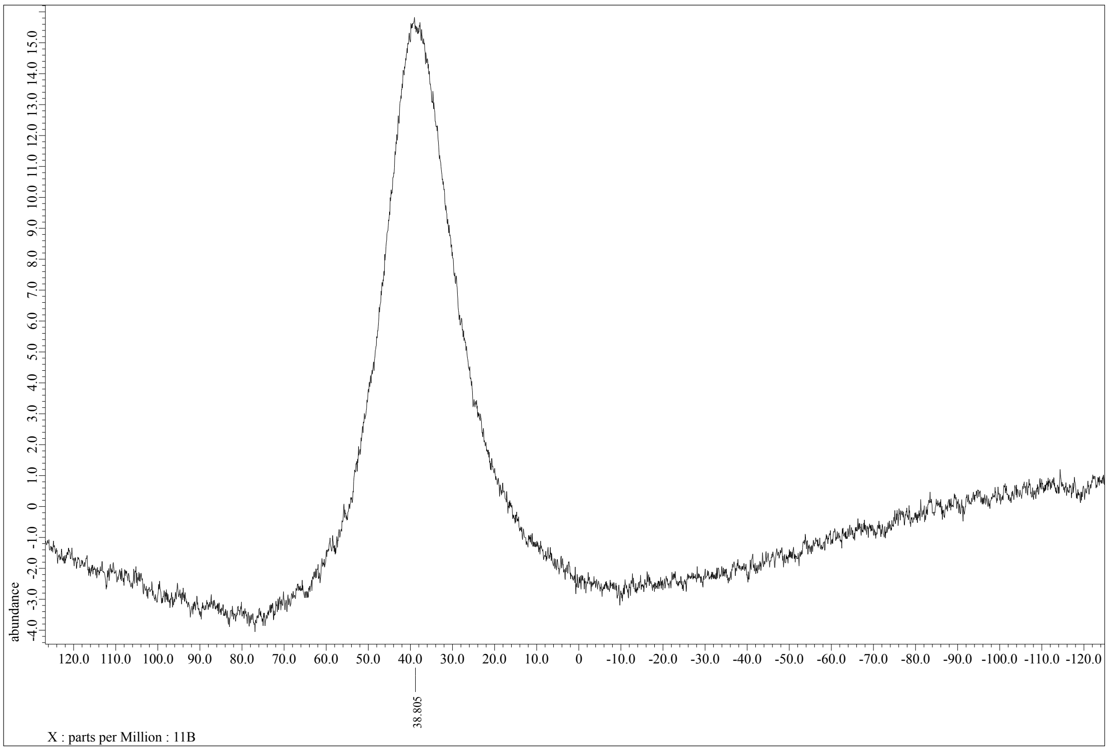

Figure S25. ^{11}B NMR spectrum of **b** in $CDCl_3$ at 25 °C.

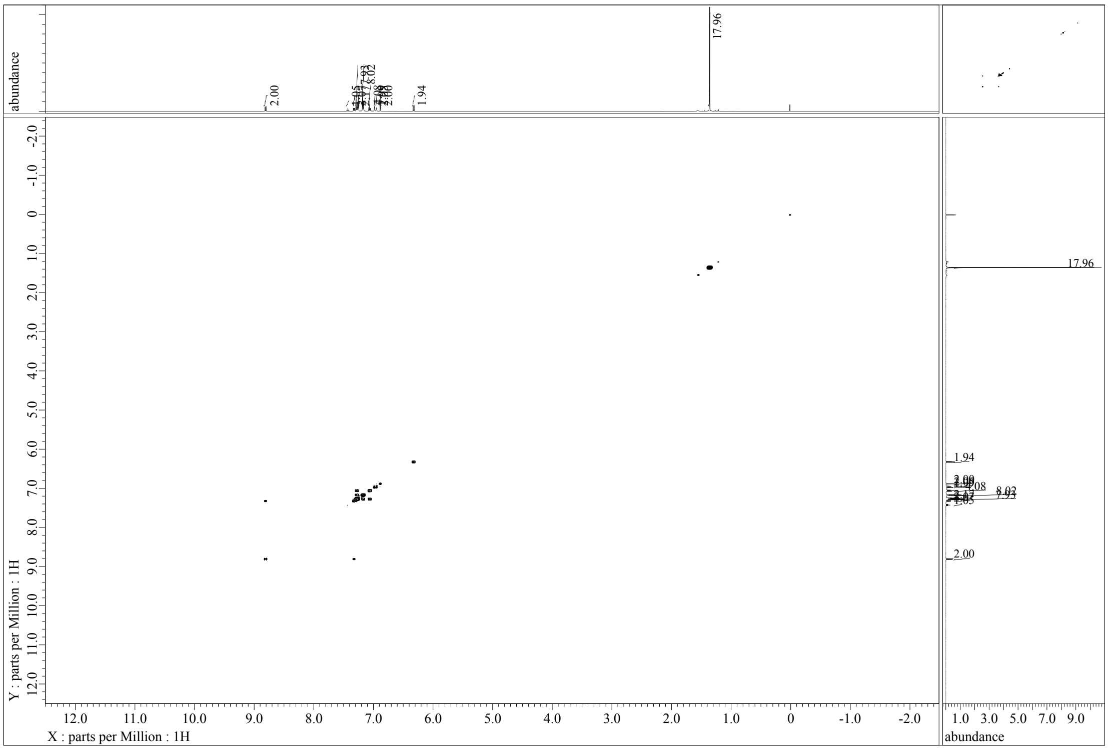

Figure S26. COSY NMR of **b** in $CDCl_3$ at 25 °C.

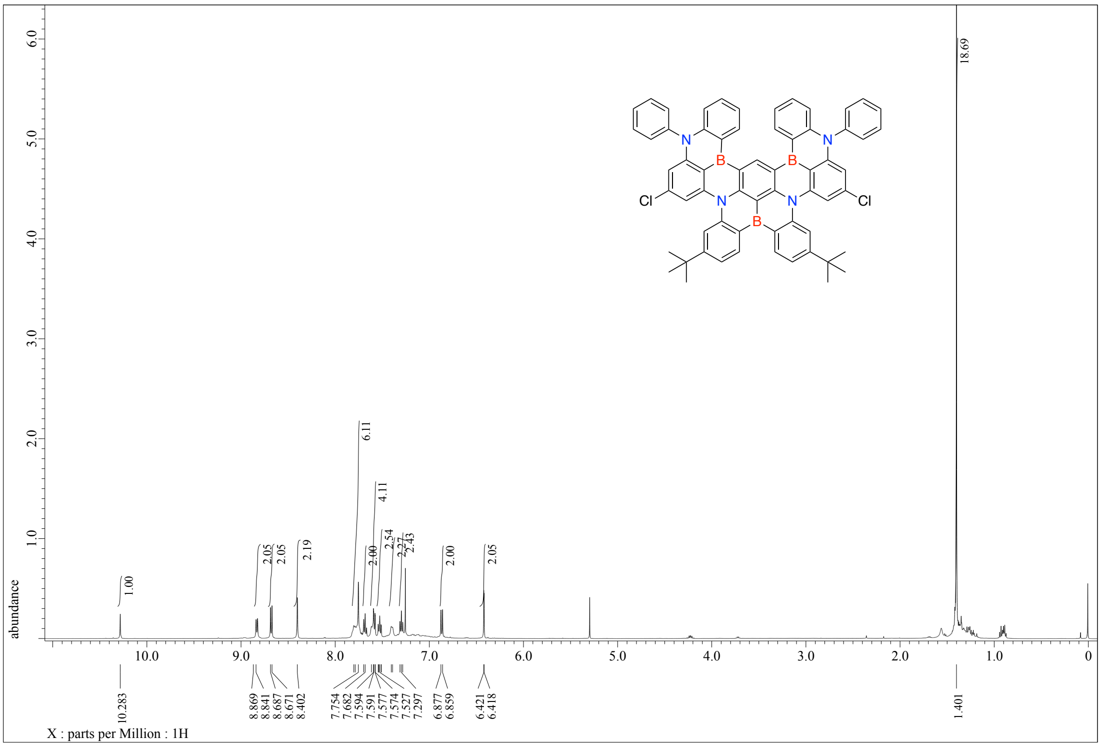

Figure S27. ^{1}H NMR spectrum of **ω-DABNA-Cl** in $CDCl_3$ at 25 °C.

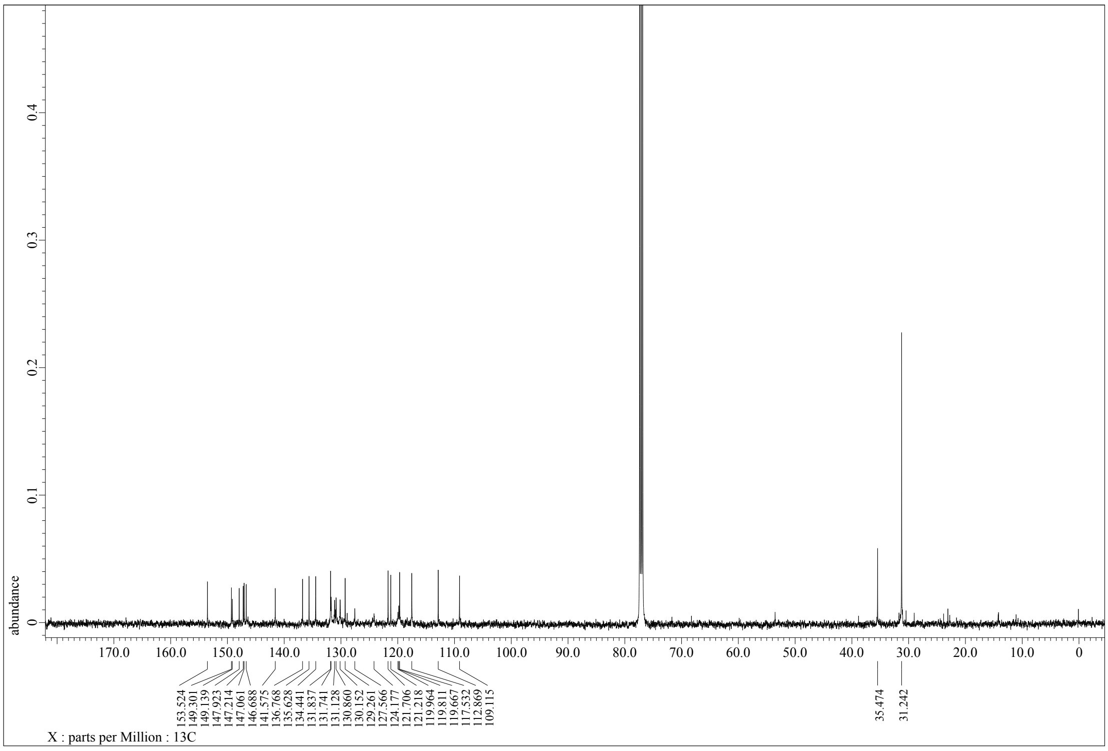

Figure S28. ^{13}C NMR spectrum of **ω-DABNA-Cl** in $CDCl_3$ at 25 °C.

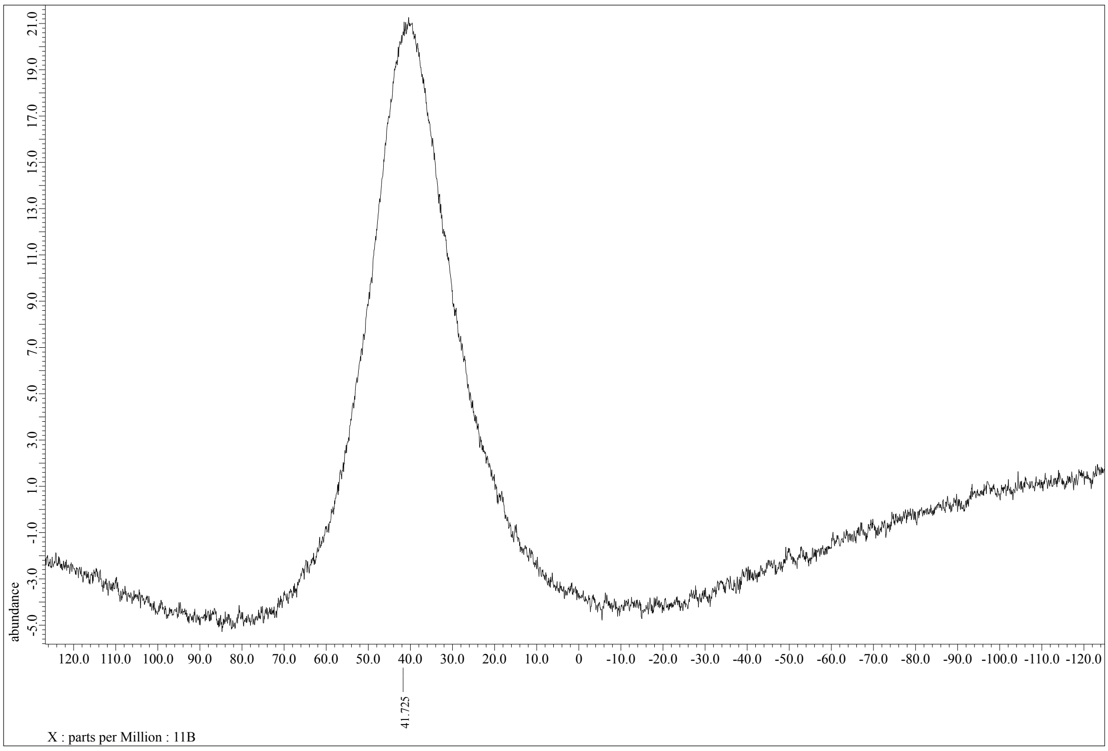

Figure S29. ^{11}B NMR spectrum of **ω-DABNA-Cl** in $CDCl_3$ at 25 °C.

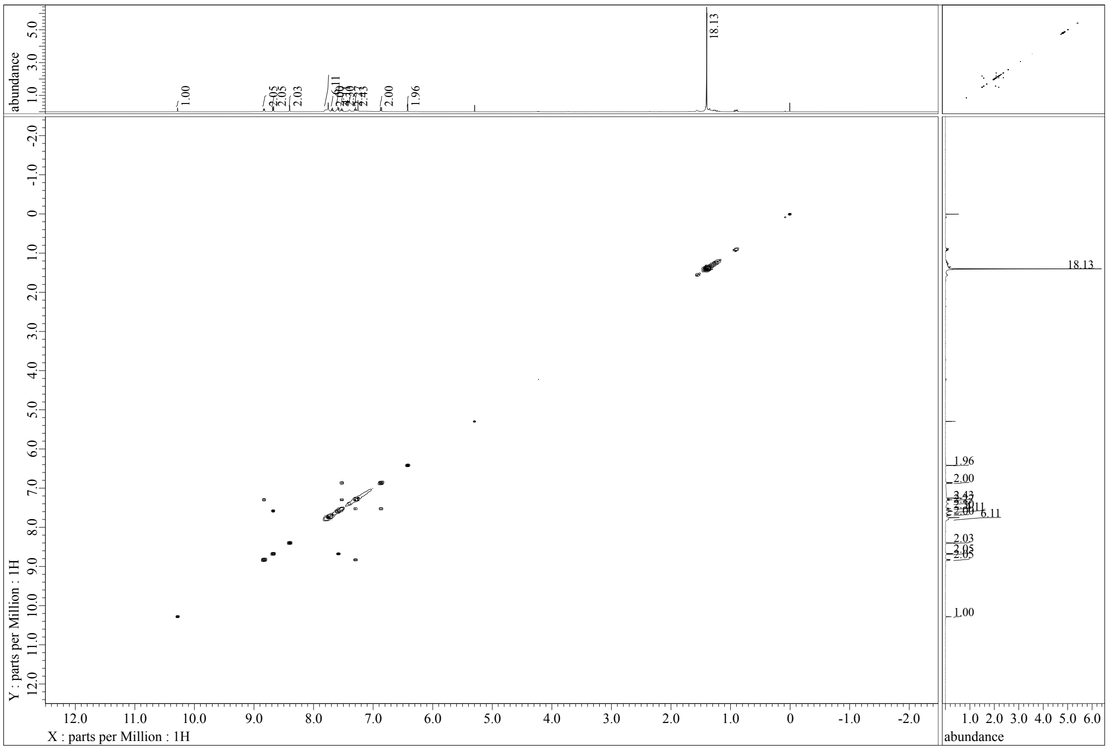

Figure S30. COSY NMR of **ω-DABNA-Cl** in $CDCl_3$ at 25 °C.

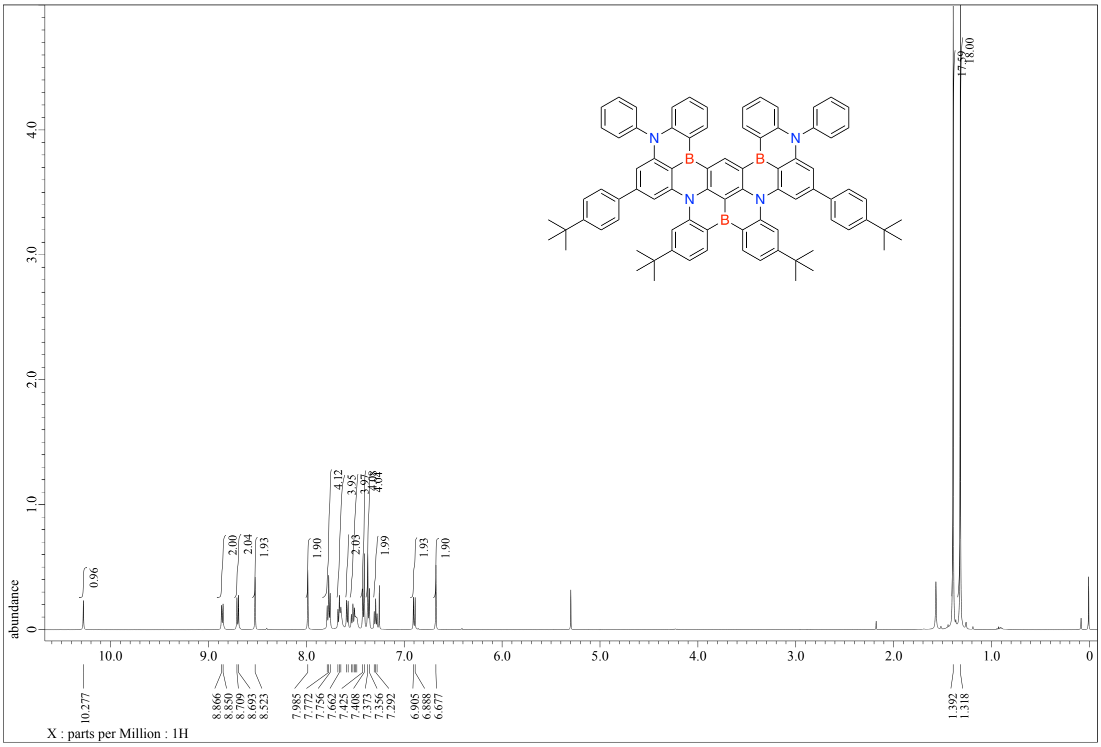

Figure S31. ^{1}H NMR spectrum of **ω-DABNA-4TBP** in $CDCl_3$ at 25 °C.

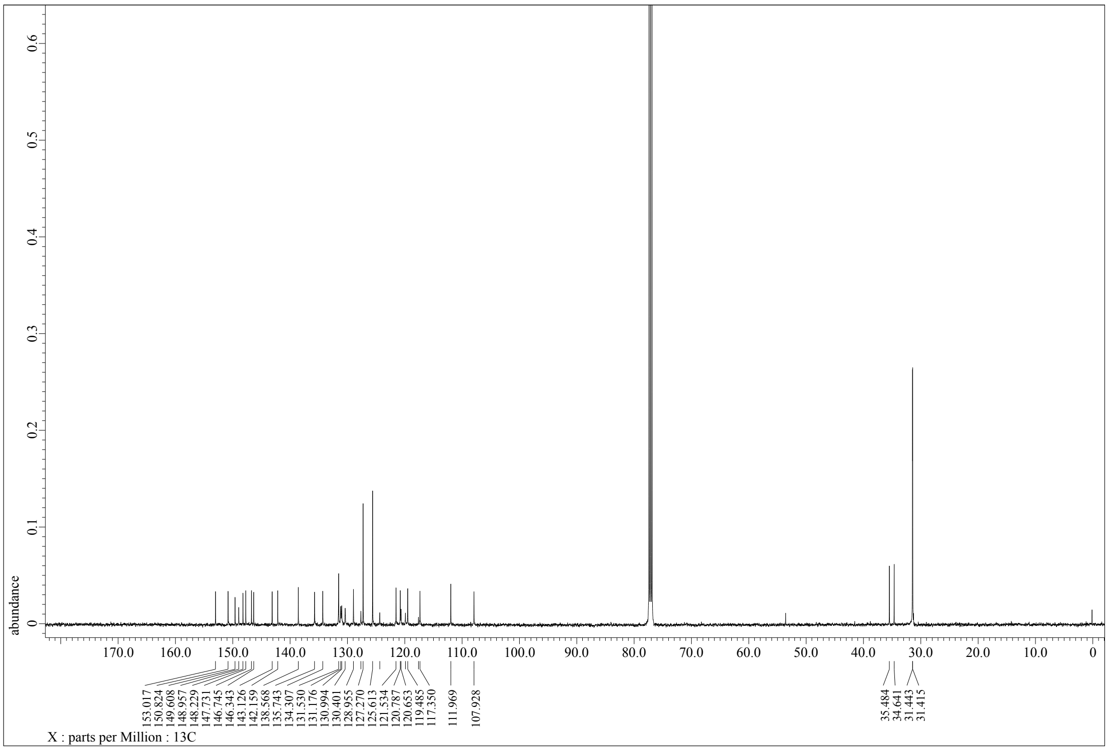

Figure S32. ^{13}C NMR spectrum of **ω-DABNA-4TBP** in $CDCl_3$ at 25 °C.

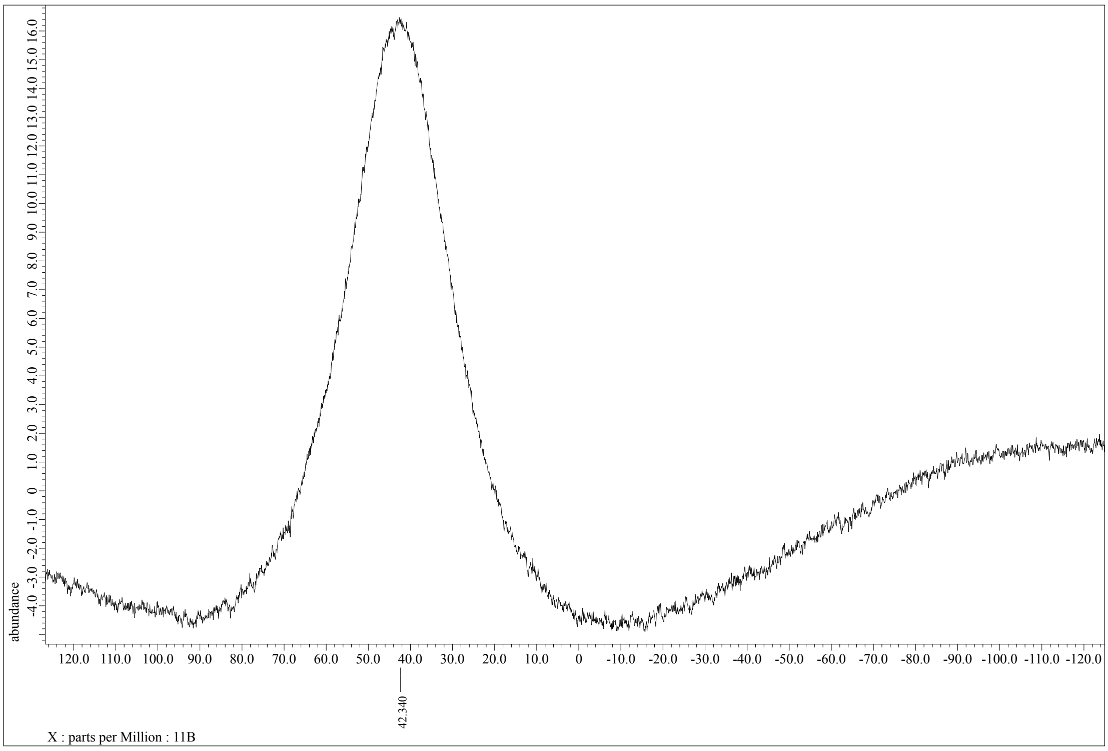

Figure S33. ^{11}B NMR spectrum of **ω-DABNA-4TBP** in $CDCl_3$ at 25 °C.

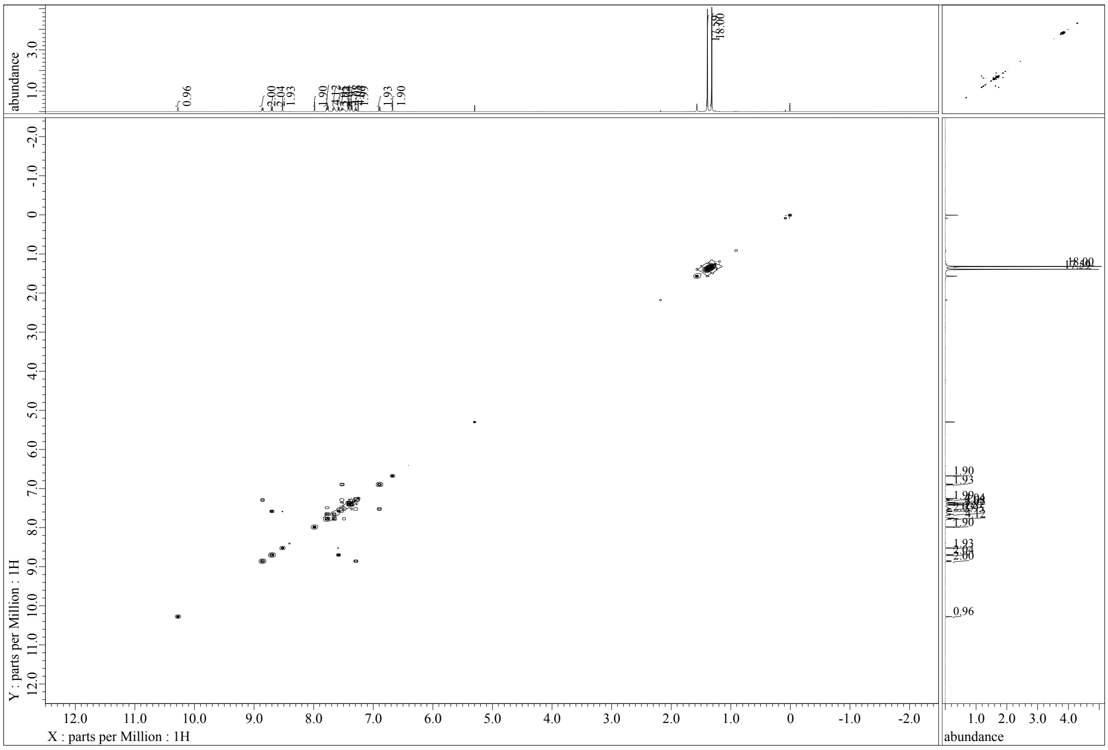

Figure S34. COSY NMR of **ω-DABNA-4TBP** in $CDCl_3$ at 25 °C.

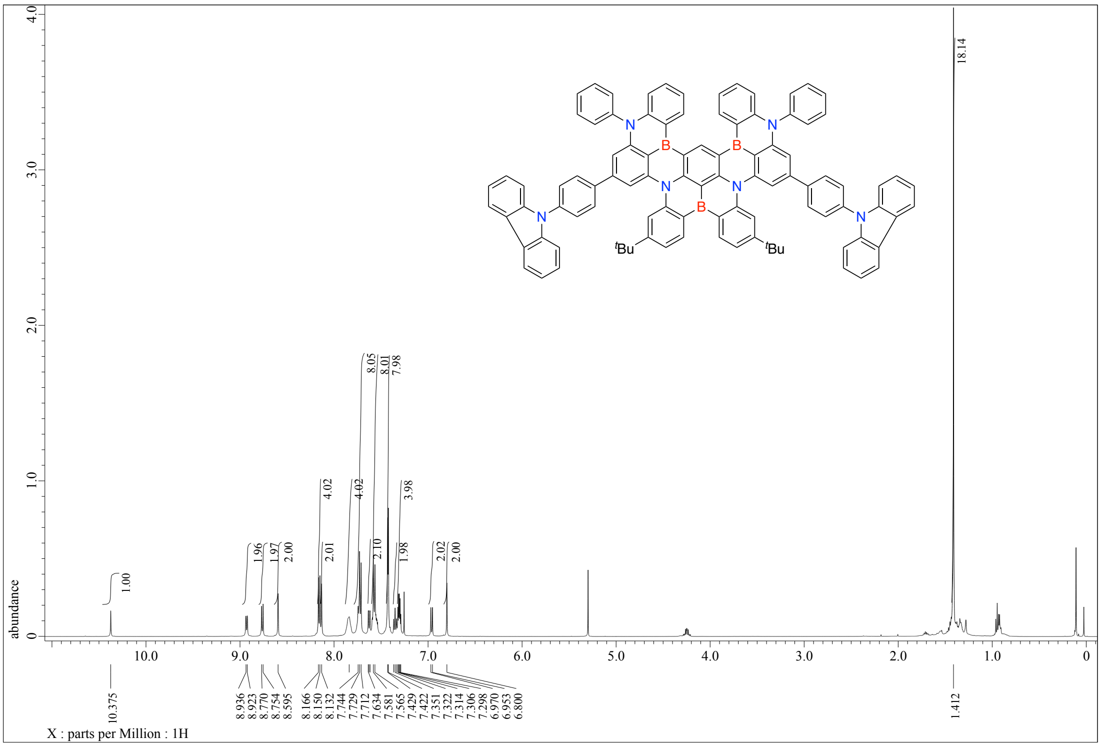

Figure S35. ^{1}H NMR spectrum of ω-**DABNA-4CzP** in $CDCl_3$ at 25 °C.

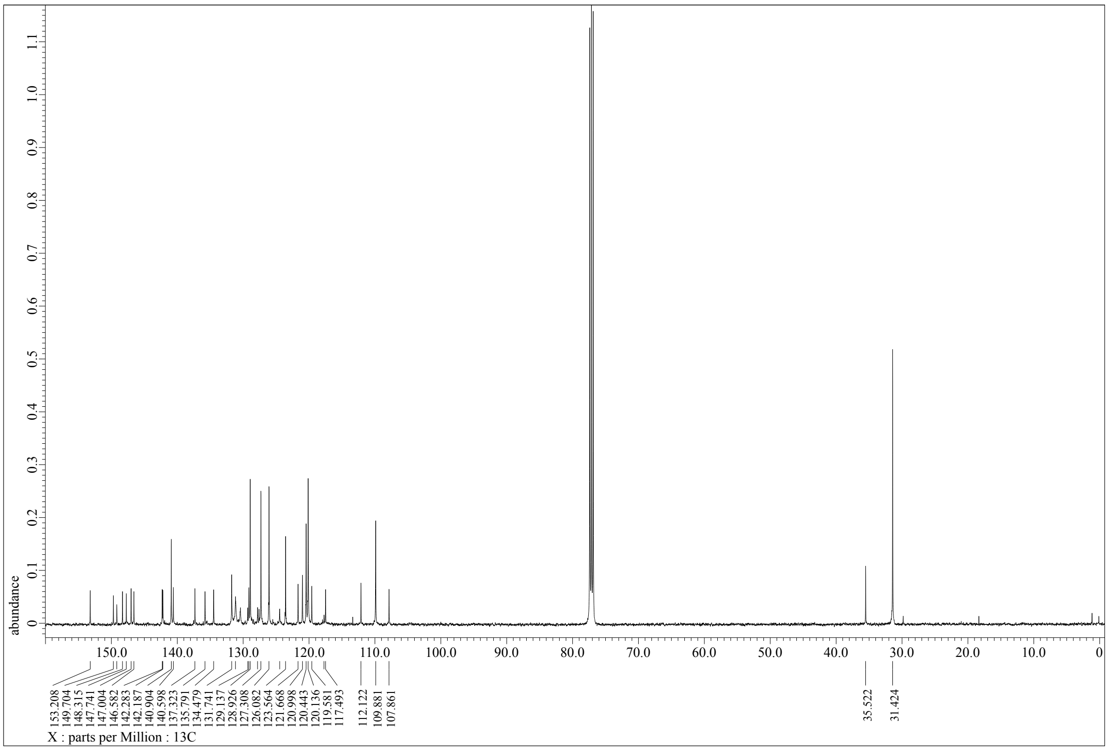

Figure S36. ^{13}C NMR spectrum of **ω-DABNA-4CzP** in $CDCl_3$ at 25 °C.

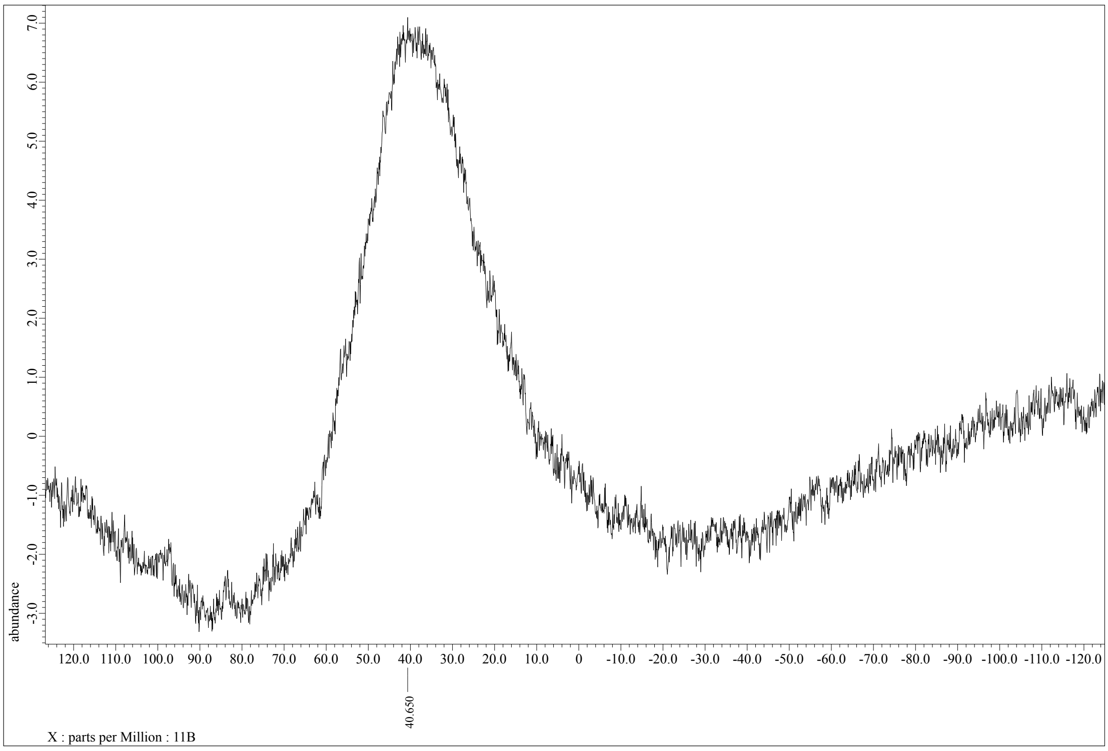

Figure S37. ^{11}B NMR spectrum of **ω-DABNA-4CzP** in $CDCl_3$ at 25 °C.

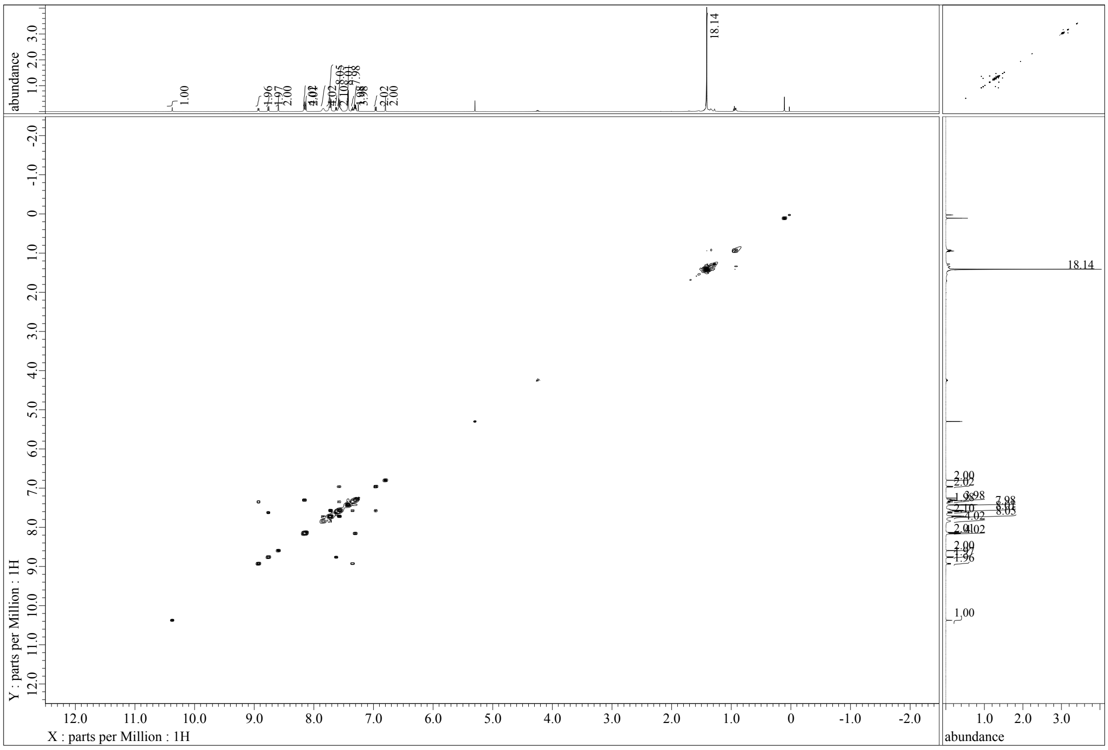

Figure S38. COSY NMR of **ω-DABNA-4CzP** in $CDCl_3$ at 25 °C

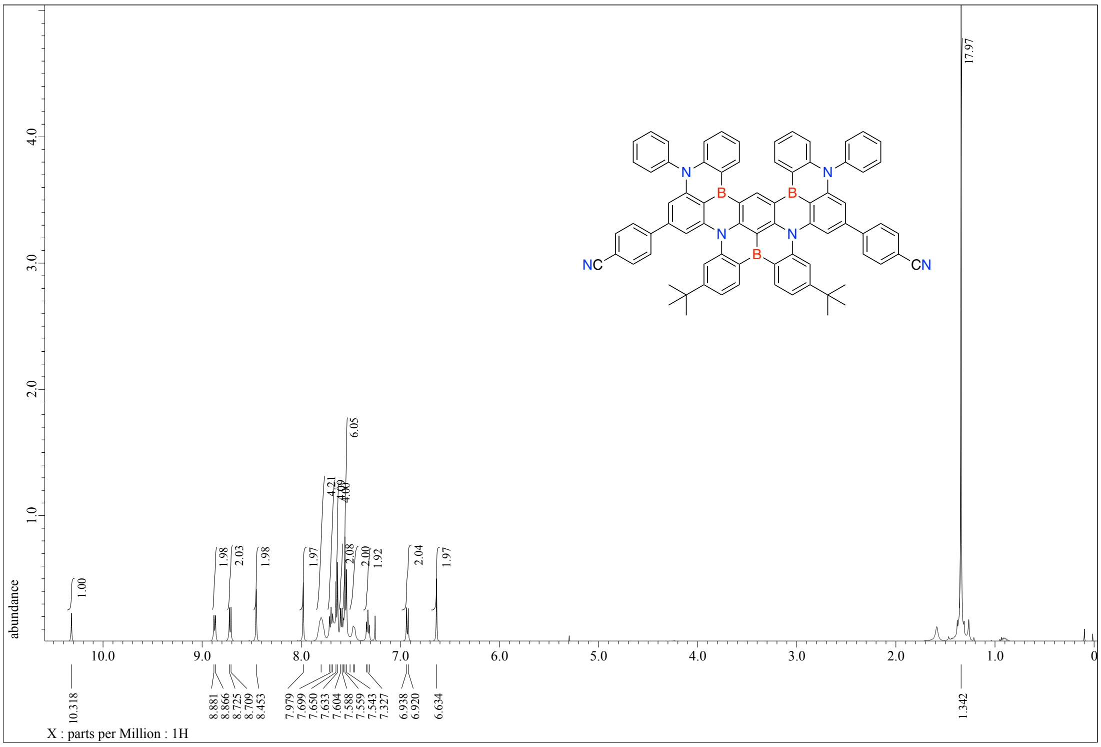

Figure S39. ^{1}H NMR spectrum of ω-**DABNA-4CNP** in $CDCl_3$ at 25 °C.

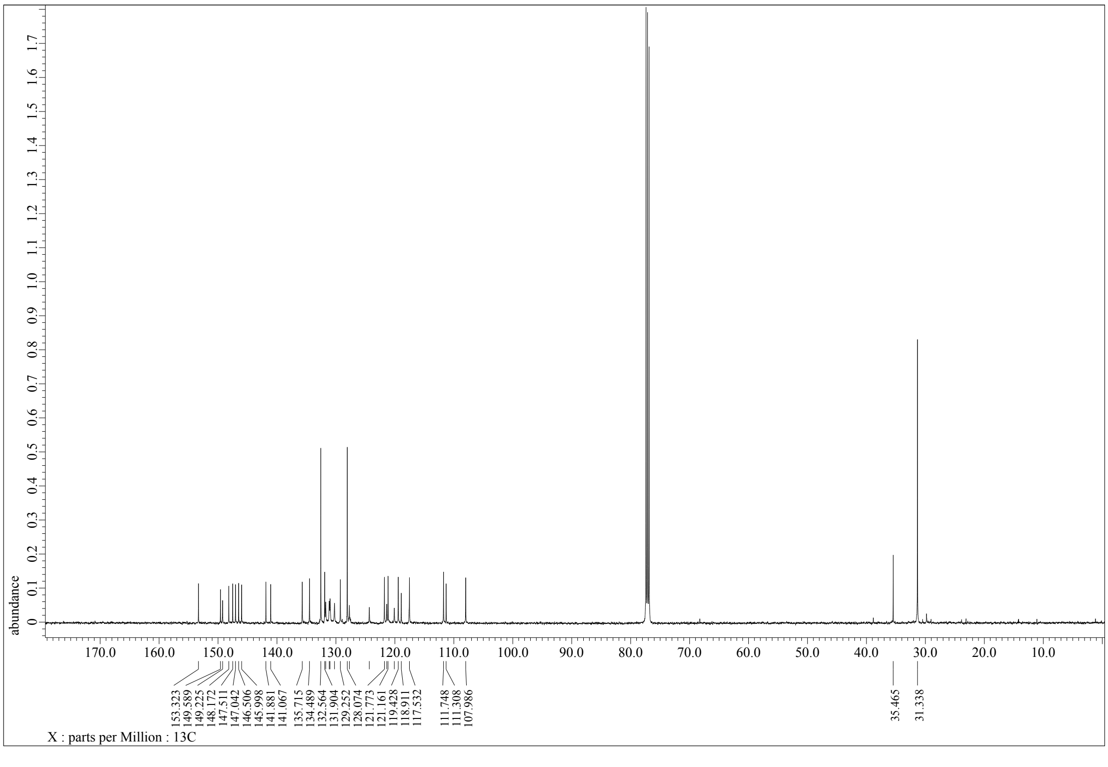

Figure S40. ^{13}C NMR spectrum of **ω-DABNA-4CNP** in $CDCl_3$ at 25 °C.

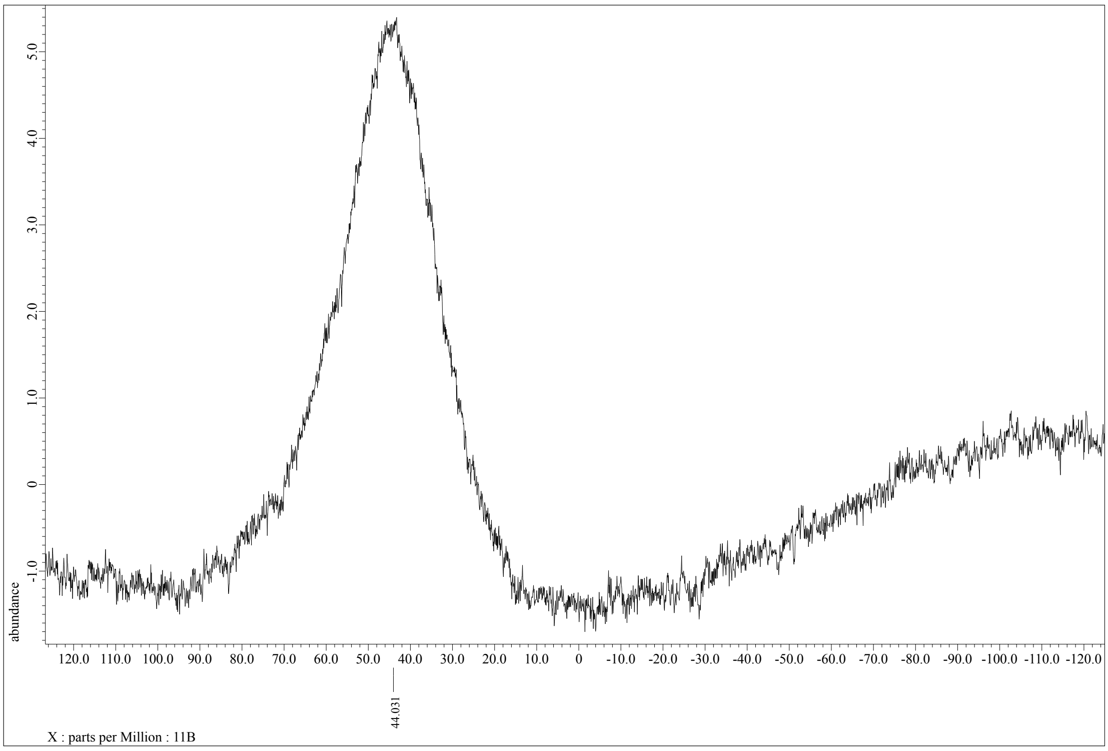

Figure S41. ^{11}B NMR spectrum of **ω-DABNA-4CNP** in $CDCl_3$ at 25 °C.

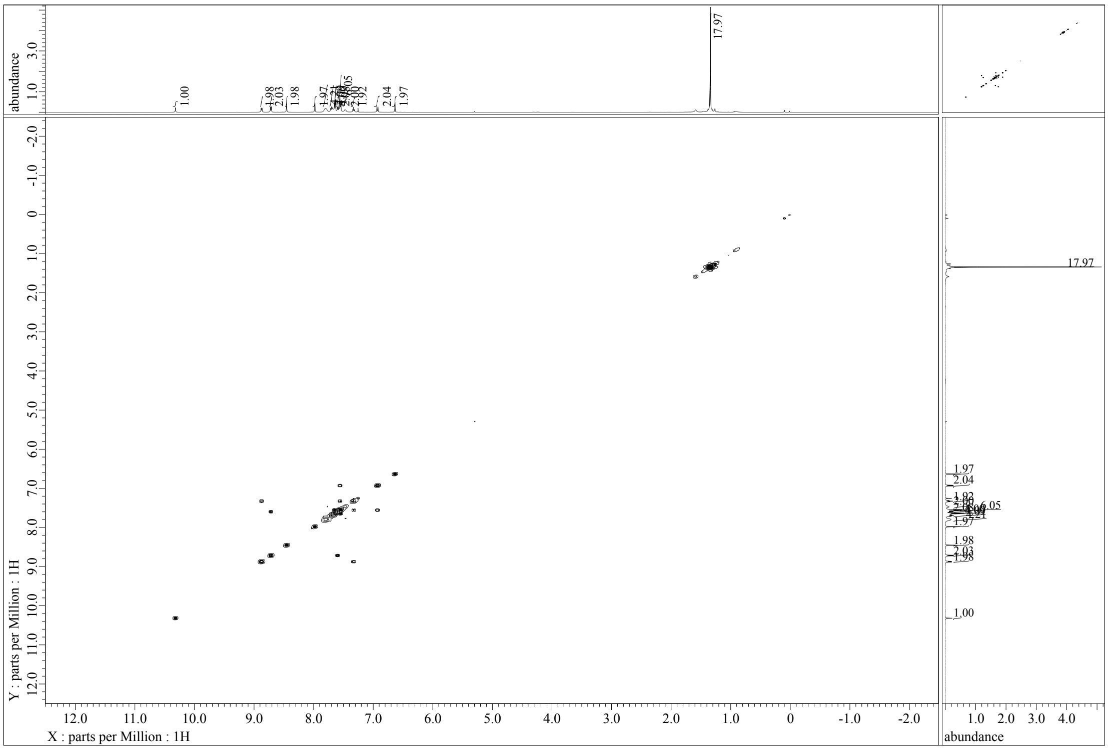

Figure S42. COSY NMR of **ω-DABNA-4CNP** in $CDCl_3$ at 25 °C.
